# Two different jumping mechanisms of water striders are determined by body size

**DOI:** 10.1073/pnas.2219972120

**Published:** 2023-07-18

**Authors:** Woojoo Kim, Juliette Amauger, Jungmoon Ha, Thai Hong Pham, Anh Duc Tran, Jae Hong Lee, Jinseok Park, Piotr G. Jablonski, Ho-Young Kim, Sang-im Lee

**Affiliations:** ^a^Laboratory of Behavioral Ecology and Evolution, School of Biological Sciences, Seoul National University, Seoul 08826, Korea; ^b^Laboratoire d’Hydrodynamique de l’X (LadHyX), UMR CNRS 7646, École Polytechnique, 91128 Palaiseau Cedex, France; ^c^Mientrung Institute for Scientific Research, Vietnam National Museum of Nature, Vietnam Academy of Science and Technology, 49000 Hue, Vietnam; ^d^Graduate University of Science and Technology, Vietnam Academy of Science and Technology, 100000 Hanoi, Vietnam; ^e^Department of Applied Zoology, Faculty of Biology, University of Science, Vietnam National University, 11414 Hanoi, Vietnam; ^f^Department of Mechanical Engineering, Seoul National University, Seoul 08826, Korea; ^g^Museum and Institute of Zoology, Polish Academy of Sciences, 00-679 Warsaw, Poland; ^h^Institute of Advanced Machines and Design, Seoul National University, Seoul 08826, Korea; ^i^Laboratory of Integrative Animal Ecology, Department of New Biology, Daegu Gyeongbuk Institute of Science & Technology, Daegu 42988, Korea; ^j^New Biology Research Center, Daegu Gyeongbuk Institute of Science & Technology, Daegu 42988, Korea

**Keywords:** locomotion, water strider, allometry, jumping, biomechanics

## Abstract

Current theory for surface tension-dominant jumps on water, created for small- and medium-sized water strider species and used in bioinspired engineering, predicts that jumping individuals are able to match their downward leg movement speed to their size and morphology such that they maximize the takeoff speed and minimize the takeoff delay without breaking the water surface. Here, we use empirical observations and theoretical modeling to show that large species (heavier than ~80 mg) could theoretically perform the surface-dominated jumps according to the existing model, but they do not conform to its predictions, and switch to using surface-breaking jumps in order to achieve jumping performance sufficient for evading attacks from underwater predators. This illustrates how natural selection for avoiding predators may break the theoretical scaling relationship between prey size and its jumping performance within one physical mechanism, leading to an evolutionary shift to another mechanism that provides protection from attacking predators. Hence, the results are consistent with a general idea: Natural selection for the maintenance of adaptive function of a specific behavior performed within environmental physical constraints leads to size-specific shift to behaviors that use a new physical mechanism that secure the adaptive function.

## Significance

Water striders live on the water surface and jump to escape from underwater predators. They utilize the surface tension to achieve high jump efficiency from the water surface without breaking it. We report that the world’s largest water striders jump with breaking the water surface. Our theoretical model shows that it is beneficial for larger species to use both drag and surface tension in jumping with water surface breaking, whereas small species only benefit from surface-tension-dominant jump. The simulation results and empirical observations demonstrate that these two behavioral phenotypes based on different physical principles are the outcome of predation pressure toward the water striders of different body sizes who spent life on the surface of water.

Scaling relationships among morphological traits, the biomechanical mechanisms in which they are used, and the adaptive behaviors they serve, are the outcomes of combinations of organism’s biological features, physical constraints from the organism’s environment, and the nature of traits’ functions (1–13). Compared to the allometry among morphology and structural components (4, 6, 8), the allometric relationship between the morphology and behavioral/biomechanical mechanisms is relatively less studied. Surface tension–dominant locomotion of water striders (14–20) provides a unique opportunity to study the relationship between morphology and behavior that clearly serves an antipredatory function under the constraints imposed by the physical properties of water surface.

**Fig. 1.**
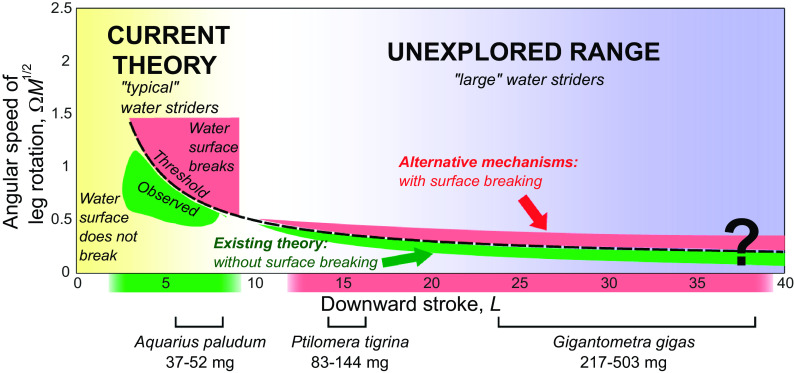
Graphical explanation of the research aims. Theoretical model (19) proposes an optimized surface tension jumping strategy for smaller water strider species weighing up to ~50 mg (indicated by the yellow shaded area on the *Left* side of the panel). These species have leg lengths up to ~3 cm, which corresponds to dimensionless downward strokes of up to ~10 ( $L=\Delta l_{l}/l_{c}$ ; explanations of mathematical symbols are in Table 1) indicated by the green shaded area under the horizontal axis. Angular velocity of leg rotation during a jump, ω , is expressed as a nondimensional variable, $\Omega =\omega {\left(l_{c}/g\right)}^{1/2}$ and is combined with a nondimensional measure of body mass, $M=m/(\rho l_{c}^{2}Cl_{w})$ , into one function, $\Omega M^{1/2}$ . Yang et al. (19) empirically determined that the angular speeds of downward leg rotation by the “typical” water striders locate in the observed green shaded area under the black broken line marking the threshold described by the formula: $\Omega M^{1/2}=4/L+0.1$ . The pink shaded area above the threshold line represents jumps that lead to the breaking of water surface and lower jump performance (19). $\Omega M^{1/2}$ was treated by Yang et al. (19) as an index of angular speed of leg downward movement rotation because an individual water strider has control over their leg speed but not body weight. We asked whether two large subtropical water strider species, *Gigantometra gigas* ( L up to 40; body weight 217 to 503 mg) and *Ptilomera tigrina* ( L between 14 and 16; body weight 83 to 144 mg), use relatively low angular speeds of midleg rotation (green shaded area below the threshold line) and follow the same physical principles for surface tension powered jumps as the small species, or they jump with water surface breaking by using higher angular speeds of midleg rotation resulting in $\Omega M^{1/2}$ value above the threshold line.

Water striders (Gerridae) are true bugs (Insecta: Hemiptera) that live on the surface of water (21). They experience physical constraints on locomotion as water surface can break when the load exceeds the force resulting from surface tension of water (17–19). Studies on several water strider species have shown that they are able to jump up vertically from the unbroken water surface (18, 19, 22, 23) in response to predatory attacks from below (24, 25). These species are known to have a Weber number around 0.1 (16, 26, 27), indicating that their jumping thrust is mainly derived from surface tension rather than drag force. The theoretical model (19) allows us to understand how water striders optimize their jumping performance within the physical constraints of water surface properties. It predicts that water surface breaks during a jump if the value of $\Omega M^{1/2}$ exceeds 4/L+0.1 (a threshold indicated with the black broken line in Fig. 1; mathematical symbols are explained in Table 1 and the basic formulae are explained in the caption of Fig. 1). The function involves three dimensionless variables (19): downward stroke ( L ; an indication of how far the leg can reach downward during a jump), angular leg velocity ( Ω ), and body mass ( M ). Water striders adjust the angular velocity of their downward leg movements ( Ω ) to the species-specific downward stroke, L , that largely depends on the midleg length, and to the species-specific body mass ( M ) such that they maximize the takeoff speed and minimize the takeoff delay without breaking the water surface. This optimal behavior Ω observed in small and medium water strider species is marked as the green-shaded “observed” area of jumps located just under the theoretical threshold in Fig. 1 (19).

**Table 1.** Explanations of the symbols used in the model and present in the main text. Full list of all symbols with descriptions is presented in *SI Appendix*, Table S11

Explanations of the symbols appearing in the main text	
$L=\Delta l_{l}/l_{c}$	Downward stroke: dimensionless maximal reach of the average of two midlegs [scaled by the capillary length, $l_{c}$ (originally used in ref. 19)]
$\Omega =\omega {\left(l_{c}/g\right)}^{1/2}$	Dimensionless angular velocity of the average four legs’ rotation of a jump (originally used in ref. 19)
$M=m/(\rho l_{c}^{2}Cl_{w})$	Dimensionless index of insect body mass with respect to the leg; body mass with respect to maximal water mass can be displaced by the average of four legs (originally used in ref. 19)
$L_{m}=\Delta l_{l}/l_{c}$	Midleg downward stroke; dimensionless maximal reach of the midleg (modified *L* for midleg only)
$\Omega_{m}=\omega_{e}{\left(l_{c}/g\right)}^{1/2}$	Dimensionless angular velocity of midleg rotation of a jump (modified Ω for midleg only)
$M_{m}=m/\left(\rho l_{c}^{2}C_{m0}l_{m}\right)$	Dimensionless index of insect body mass with respect to the midleg; body mass with respect to maximal water mass can be displaced by the midleg (modified *M* for midleg only)
ω	Angular velocity of midleg rotation of a jump
$\omega_{e}$	Derived angular velocity of midleg rotation of the empirical jump
$\omega_{t}$	Hypothetical angular velocity of midleg rotation of the hypothetical jumps (i.e., surface tension jumps of *Gigantometra gigas* and *Ptilomera tigrina*; drag-involving jump of *Aquarius paludum*)
$\omega_{c}$	Critical angular velocity of leg rotation; For a given midleg length and body mass, descending midleg can produce a dimple of the critical dimple depth, $h_{c}$ , with $\omega_{c}$
$D_{b}$	Duration of dimple breaking
$l_{c}={\left[\sigma /(\rho g)\right]}^{1/2}$	Capillary length
$\Delta l_{l}=l_{l}-y_{i}$	Maximal downward reach of the midleg
$l_{w}$	Wetted length of the leg
$l_{l}$	Entire length of the midleg consisting of femur, tibia, and tarsus
$l_{m}$	Constant wetted length of midleg (the length of tibia plus tarsus of the midleg)
$y_{i}$	Initial height of the body center from the undisturbed free surface
σ	Surface tension coefficient of water
ρ	Density of water
g	Gravitational acceleration
m	Mass of the water strider
C	Flexibility factor; function of wetted length of a leg, $l_{w}$ , and its bending rigidity, B
$C_{m0}$	Midleg flexibility factor; function of wetted length of a midleg, $l_{m}$ , and its bending rigidity, B (bending rigidity is explained in *SI Appendix*, Table S11).
E	Young’s modulus of insect cuticle
r	Radius of the wetted midleg as a cylinder
$r_{b}$	Radius of the wetted midleg as a cylinder surrounded by air bubble

The jumping behavior was studied in only several Palearctic/Nearctic water strider species with body weights below 50 mg (18, 19, 22, 23), which corresponds to midleg lengths smaller than L = 10 (referred to as typical mid-size water striders; *SI Appendix*, Fig. S1). They represent a fraction of the morphological diversity among Gerridae including large species in subfamilies Gerrinae and Ptilomerinae (*SI Appendix*, Fig. S1*B*). We were interested in the applicability of this theory to the jumps of the larger sized water striders (“unexplored range” shaded in violet in Fig. 1 and see also *SI Appendix*, Fig. S1). We considered two feasible mechanisms involved in jumps of the large water striders (Fig. 1): a) according to the current theory, the large water striders do not break the water surface when they jump, or b) the large water striders break the water surface resulting in different biomechanics, perhaps similar to the basilisk lizards running on water (28, 29) or fishing spider galloping and jumping on water (30). We suspected that the second mechanism is possible because the large body size may cause a shift in the jumping mechanism toward a relatively high role of drag forces [i.e., mechanisms characterized by the higher Weber number (16)].

**Fig. 2.**
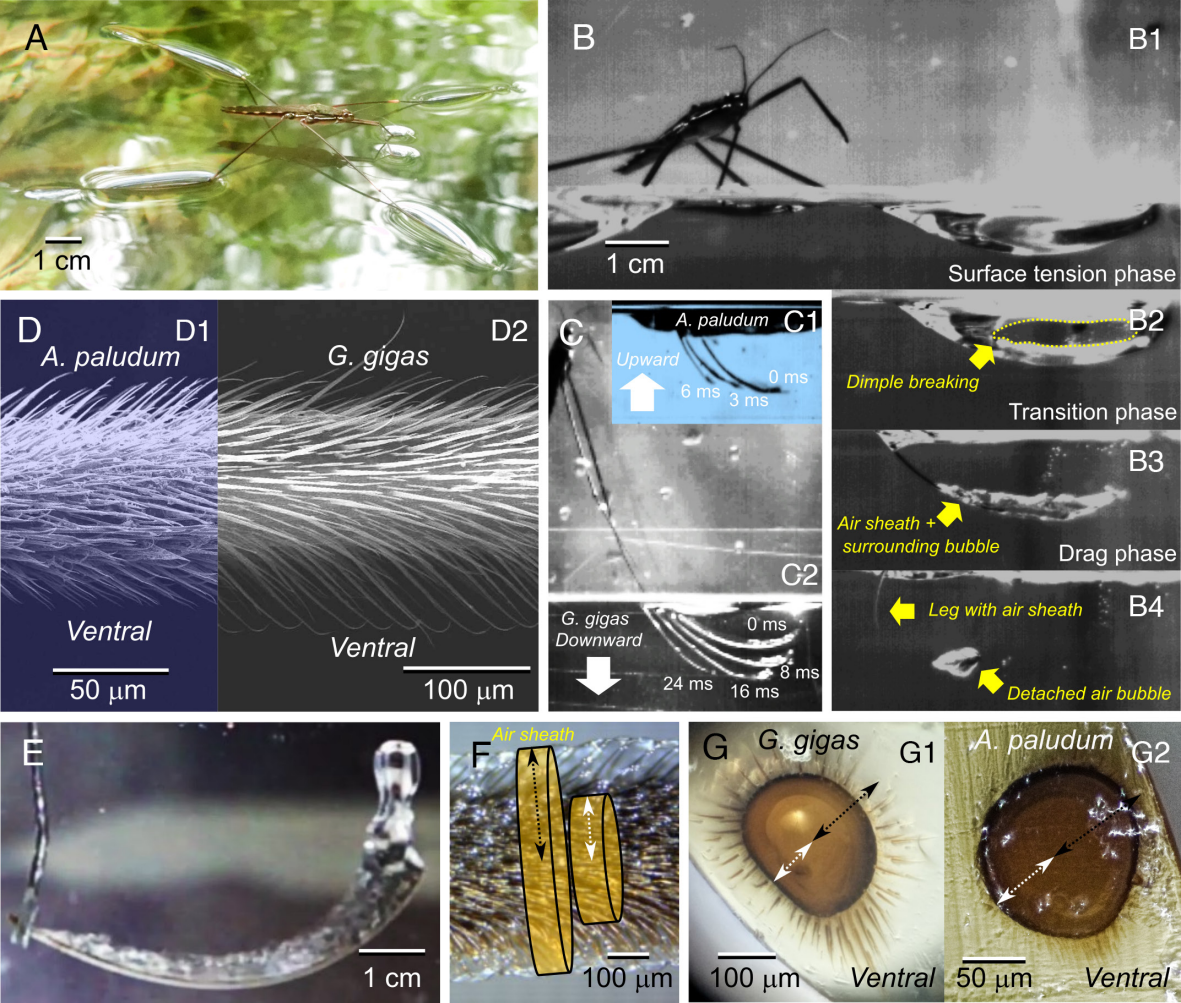
Photographic explanation of how the giant water strider (*G. gigas)* jumps on water, including morphological adaptations on midlegs to capture air during penetration of the water. (*A*) *G. gigas* on the water surface. The hindleg’s tibiae and tarsi press the surface downward and create dimples during jumping; (*B*) The midleg’s femur+tibia+tarsi functional unit moves downward while bending and deforming the surface of water to create a dimple (*B1*), which eventually starts to break (*B2*), and each midleg continues to operate as a bending rod-like functional unit pushing down in the water after complete breaking (*B3*) and creating upward drag force. Air sheath is caught among the long hairs on midleg’s tibia and tarsus (*D2*, *F*, and *G1*) and an additional air bubble surrounds the legs (*B3*), contributing to the drag force. Finally, the midlegs slide out and leave air bubbles (*B4*). (*C*) Stacked frames from a jump, starting with the moment right after surface breaking (0 ms) in *A. paludum* and *G. gigas*; in *A. paludum,* midlegs move upward after breaking (*C1*), the legs of *G. gigas* move downward in the water (here up to 16 ms from the moment of breaking the surface); (*D*) SEM image of midleg tibia of the giant water strider (*D2*) compared with *A. paludum* (*D1*); (*E*) a frame from a high-speed movie (Movie S1) of the midleg experimentally pushed downward in the water to illustrate the presence of air bubble surrounding the fast-moving leg; (*F*) midleg tibia in water in static situation: the layer of air sheath captured in the hairs around the leg increases the effective radius of the leg; (*G*) cross-section of the midleg’s tibia to illustrate the distribution of hairs: relatively shorter hair on *A. paludum* (*G2*), and longer hair on *G. gigas* (*G1*). In (*F*) and (*G*), the white broken line with arrowheads indicates the actual radius of the leg while the black broken line with arrowheads indicates the effective radius that captures air sheath and creates drag force (with additional air bubble caught during leg downward). The radius of leg with hair capturing air sheath is marked as r , and the radius of leg with the surrounding air bubble is marked as $r_{b}$ in the model and in Fig. 5. Photo credits: P. G. Jablonski, J. Ha, W. Kim & S.-i. Lee.

We first focused on the world’s largest water strider, *Gigantometra gigas* [Fig. 2*A*; (31, 32)], to study their jumping in natural habitats and to provide a theoretical model of the biomechanics of jumping on water by these heavy water striders. After confirming that the giant water striders break the water surface during jumping (second mechanism), we built a theoretical model to predict the water strider’s body size at which the allometric switch (from the first mechanism to the second mechanism) is expected, and we tested the predictions using observations of jumps in another previously unstudied large species, *Ptilomera tigrina,* with body mass of 83 to 144 mg, as well as in the previously studied typical medium-sized water strider *Aquarius paludum* with body mass of 37 to 52 mg.

## Results and Discussion

### Empirical Observations and Kinematics of Jumps in *G. gigas*.

**Fig. 3.**
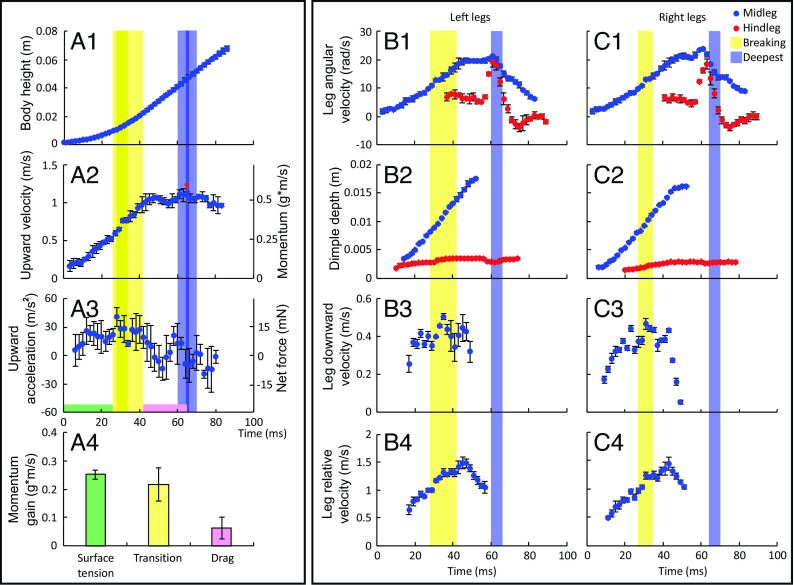
Empirical analysis of the kinematics and dynamics of the jumping on water by the giant water strider, *G. gigas*. (*A*) variables obtained from the body movement: changes in body height above the water surface (*A1*), body velocity (*A2*) and body acceleration (*A3*) during the jump. Right side axes in *A2* and *A3* indicate the changes in body momentum (*A2*) and net force (*A3*) during the jump calculated from the body movement and body mass. *A4* shows the comparison between the values of momentum gained during the three phases of jump: the surface tension phase (green), the transition phase (yellow), and the drag phase (purple). (*B* and *C*) contain variables concerning the *Left* (*B*) and *Right* (*C*) midlegs (blue circles) and hindlegs (red circles in *B1*, *B2*, *C1*, and *C2*): angular downward velocity (*B1* and *C1*), depth (*B2* and *C2*), downward velocity (*B3* and *C3*) and downward velocity relative to the body position (*B4* and *C4*). Yellow band across the panels indicates the transition phase for *Left* and *Right* separately in (*B*) and (*C*), which are overlaid on each other in (*A*). Blue bands across the panels indicate the bubble detaching duration for left and right leg separately. Red arrow in *A2* indicates the moment of maximal body velocity. Filled circles and error bars indicate means and SDs, respectively, from 5 independent runs of frame-by-frame manual analysis of the same clip (EVT16). Leg depth (*B2* and *C2*), leg velocity (*B3* and *C3*), and relative leg velocity (*B4* and *C4*; relative to the body center) were measured only until the moment soon after the deepest point was reached because afterward the detection of the deepest point was unreliable in the video. All the remaining variables are measured until the water strider loses contact with water. Results from analyses of two other jumps are in *SI Appendix*, Figs. S5 and S6.

The detailed research on jumping behavior was carried out on the giant water striders, *G. gigas* (Fig. 2*A*), from the population in Pu Mat National Park, Vietnam (see *SI Appendix*, Tables S1, S2, and S4 for morphological data). We were able to trigger vertical jumps in freely skating giant water striders in their natural habitat (Movie S1 and *SI Appendix*, *Supplementary Materials PARTS 2 and 3*) by imitating predator attacks from under the water surface or by creating quick movements in their visual field. We observed that *G. gigas* as well as the other large-sized water strider, *P. tigrina*, broke the water surface when they jumped on the water surface (*SI Appendix*, Tables S5 and S6). The insects jumped upward to the height of about 10 to 30 cm (2.5 to 10 times their body length). Next, we filmed 57 upward jumps from a stationary position by 17 individuals in an experimental basin setup in the field (example in Movie S1). We analyzed in full detail the three best clips with male water striders (we chose males in order to test the world-largest water striders; males are larger than females, *SI Appendix*, Table S1) facing the camera and performing relatively symmetrical (left and right) coordinated leg movements (Fig. 3 and *SI Appendix*, Figs. S5 and S6). The remaining nondigitized jumps showed generally similar characteristics comprising three phases: surface tension phase, transition phase, and drag phase (see below).

From the detailed analysis of jumps it was evident that a jump starts with the pure surface tension phase (Fig. 2*B1*), which ends at the moment when the surface starts breaking under at least one of the midlegs. The surface tension phase is then followed by a transition phase, during which the midlegs’ tarsi and tibia gradually break the water surface until they are entirely immersed in the water (Fig. 2*B2*; yellow-shaded vertical bands in Fig. 3 and *SI Appendix*, Figs. S5 and S6). After midlegs entirely break the water surface, the drag phase begins. During the drag phase, the midlegs’ tarsi and tibia surrounded by air caught within (air sheath, Fig. 2*F*) and around (air bubble, Fig. 2 *B3* and *E*) the layer of densely packed hairs (Fig. 2 *D2* and *G1*) are moving downward through the water (i.e., providing upward drag; Fig. 2 *B2*, *B3*, and *E*) pushing the body upward until the legs themselves reach the deepest point and start moving upward. The air bubble starts detaching from the midleg usually after the moment when midleg reaches the deepest point (except for only 2 cases in *SI Appendix*, Table S7). The hindlegs usually do not break the water surface, but maintain the dimple and provide the thrusting force stemming from surface tension (*SI Appendix*, Fig. S15).

A volume of air was captured by a midleg during and after surface breaking. We differentiated this volume of air into the portion caught in the “air sheath” and the portion caught in the “air bubble”. The former is the air captured inside the hair layer, which remains attached during the leg movement and the latter is the air surrounding the leg that is detached from the leg and slowly floats upward to the surface (Fig. 2*B4*; see more details in *SI Appendix*, Fig. S10). Based on the size of the detached bubbles, we evaluated that the volume of air bubble around one midleg ranges from 10 to 80 mm^3^ (*SI Appendix*, Table S8).

Finally, after the downward midleg movement stops and the leg reaches the deepest point, an additional small increase in momentum (hence, in body speed) may occur (present in Fig. 3 and *SI Appendix*, Fig. S5 but not in *SI Appendix*, Fig. S6) for several milliseconds (<10 ms). It appears that during this time hindlegs create a dimple of constant depth (*SI Appendix*, Fig. S15 *A* and *C*), and the wetted hindleg length gradually decreases. The abrupt and short increase in the angular downward velocity by hindleg’s femur (Fig. 3 *B1* and *C1* and *SI Appendix*, Fig. S5 *B1* and *C1*) is a consequence of body pitch change (head-upward/abdomen-downward; *SI Appendix*, Fig. S15*C*).

The momentum gained in the surface tension phase was from ~0.12 to ~0.28 g m/s, while the momentum values gained during the transition and drag phases were 0.20 to 0.22 g m/s and 0.04 to 0.12 g m/s, respectively (the means from the five repeated measures in each of 3 jumps/videos; Fig. 3*A4* and *SI Appendix*, Figs. S5*A4* and S6*A4*). Examination of Fig. 3 and *SI Appendix*, Figs. S5 and S6 suggests that after midlegs reached the deepest point, the momentum gain was less than 0.05 g m/s, if noticeable at all. The transition and drag phases together contributed to an increase in body speed by 0.6 to 1.1 m/s, comprising approximately 50% of the speed achieved at the end of the surface tension phase. The maximum body speed near leaving the surface was 1.1 to 1.6 m/s (red arrows in Fig. 3*A2* and *SI Appendix*, Figs. S5 and S6).

### Theoretical Model Based on the Empirical Observations.

Inspired by the observations of jumps in *G. gigas*, we created a theoretical model of water strider’s upward jumping. We modified the previous model (19) by a) considering midlegs and hindlegs separately, b) introducing transition and drag phases, in which midlegs are surrounded by air sheath and capture air bubbles, c) allowing midlegs to reach deeper dimple depths before the water surface breaks depending on their length, d) assuming that the hindlegs create only the capillary force without breaking the surface. Therefore, our model calculates upward thrust from surface tension (capillary force before breaking the surface) or/and upward drag (after completely breaking the surface) of descending midlegs while adding the surface tension from hindlegs. In the transition phase (during breaking), midlegs provide both capillary and drag force.

We assumed that the air bubble is detached from the leg after it reaches the deepest depth (*SI Appendix*, Table S7). Additionally, we assumed that the left and right legs move in a synchronized manner (this synchronization makes shorter transition phase than empirically observed) with an angular velocity of leg rotation ( ω ) calculated according to the assumptions and formulae explained by Yang et al. (19). The surface tension phase was modeled according to the existing model (19) with an addition of the role of surface tension applied on hindlegs by assuming that their dimple depth grows in the same way as the dimple of the midlegs until it reaches its constant depth specific for hindlegs (constant dimple depth, $h_{\mathrm{hm}}$ , empirically derived in *SI Appendix*, *Supplementary Materials PARTS 9 and 10*). We also permitted deeper maximum dimples for both midlegs and hindlegs (see page 38 in Supplementary Materials) owing to longer and more elastic legs in the giant water strider compared to the typical water striders (based on the empirical observations and measurements in *SI Appendix*, Figs. S12 and S13).

For a given midleg length and body mass, if the angular velocity of leg downward rotation is lower than the critical angular velocity of leg rotation, $\omega_{c}$ , the descending midleg produces a dimple that is shallower than the critical dimple depth, $h_{c}$ , at which water surface breaks. In contrast, the midleg with the angular velocity of leg rotation higher than $\omega_{c}$ , breaks the water surface because the dimple exceeds the critical depth, $h_{c}$ , at the critical moment, $t_{c}$ . The value of $h_{c}$ used in the model was determined empirically and found to depend on the size of the water strider, specifically the length of the midleg tibia and tarsus (as shown in *SI Appendix*, Fig S13*A*). When the midleg reaches the depth of $h_{c}$ , the transition phase begins. In the transition phase, the water breaking happens over the duration, $D_{b}$ , and midlegs experience both capillary and drag forces. The value of $D_{b}$ used in the model was also determined empirically to depend on the water strider size (length of midleg tibia + tarsus; *SI Appendix*, Fig. S14). After the surface is completely broken (drag phase begins), the legs are fully immersed in the water and are bent such that a large portion of midleg tarsus and tibia is roughly horizontal (Fig. 2*C2*) while descending in the water and creating upward drag force for the jump. The drag phase was modeled assuming a rod, with the length equal to the vertical downward projection of the immersed section of a bent midleg and the radius equal to either the radius of midleg’s tarsus and tibia covered with air sheath and with or without air bubble (*SI Appendix*, *Supplementary Materials PART 7*), moving downward with the speed that is a by-product of midleg’s angular velocity and the ascending water strider’s body velocity. In the drag phase, the role of surface tension on hindlegs was modeled by using the empirically derived constant dimple depth, $h_{\mathrm{hm}}$ , during the jump after the constant depth is reached, and wetted leg length, which was calculated at each moment during a jump from femur leg length and body height above the water (*SI Appendix*, Figs. S16 and S30–S32).

### Model Validation.

**Fig. 4.**
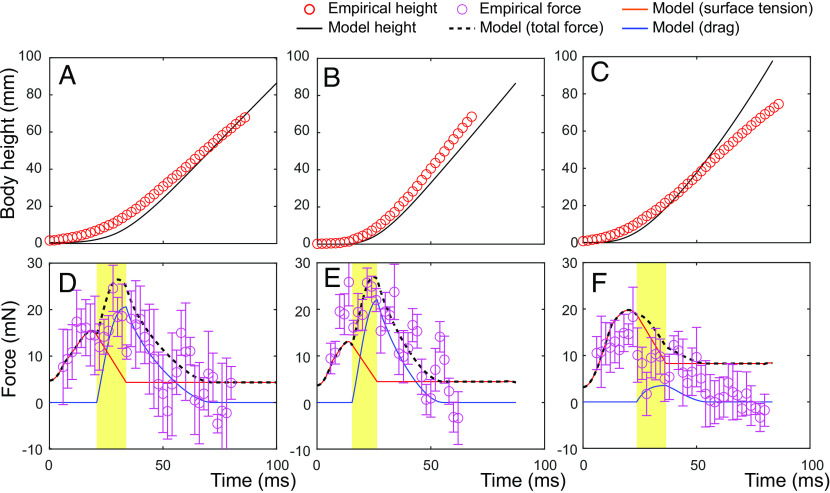
Comparison of the theoretical model predictions (lines) of body height trajectory and thrust force from theoretical simulations with empirically derived values (circles) from the three specific jumps of *G. gigas*. Theoretically calculated height (black solid line) and the empirically measured height (red circles) in the jumps of *G. gigas* are represented in *A*–*C* for the three analyzed videos: EVT16, EVT05 (2), and EVT41, respectively (shown in Fig. 3 and *SI Appendix*, Figs. S5 and S6). Calculated forces from the model for each video are represented in *D*–*F* for EVT16, EVT05 (2), and EVT41, respectively. The black dashed lines show the total generated force from two midlegs and two hindlegs. The orange and blue solid lines represent surface tension and drag, respectively. The purple circles represent the empirical force calculated from each movie by using body mass and acceleration with gravitational force added. The yellow shades represent the transition phase.

Using empirically derived values of the angular velocity of midleg rotation ( $\omega_{e}$ ), the model reasonably predicted the insect trajectories in the specific videos of jumping *G. gigas* males (Fig. 4 *A*–*C*). The model also provided a reasonable fit with empirically estimated upward force (Fig. 4 *D*–*F*), including the contribution of the air bubble around midleg’s tibia and tarsus during the transition and drag phases. To expand the model for the smaller species, we tested the model predictions using an extra assumption that regardless of the species/body size, the ratio of wetted midleg radius with air bubble to the radius without air bubble is equal to the average value of these ratios from the fourteen individuals of *G. gigas* analyzed in detail (*SI Appendix*, Table S8). The model simulations correctly predicted body center height trajectories during empirically described jumps of *G. gigas* females and *P. tigrina* individuals (*SI Appendix*, Fig. S19). The angular velocities of midleg rotation ( $\omega_{e}$ ) for these individuals were derived from empirical observations of midleg coordinates and velocities for *G. gigas*, *P. tigrina*, and also *A. paludum* (*SI Appendix*, Figs. S20–S23).

### Model Simulations of Jumps for Four Size Classes.

**Fig. 5.**
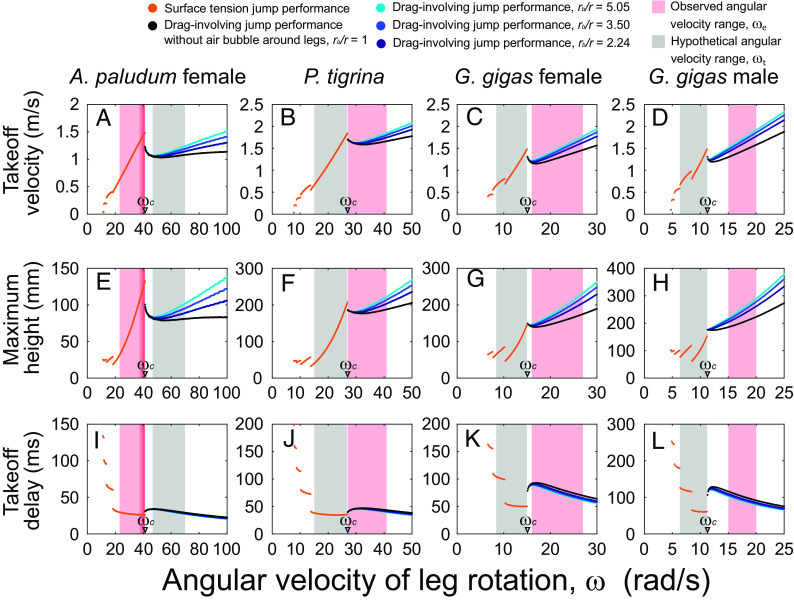
Theoretically predicted jump performance as a function of midleg angular velocity for four classes of water striders’ body size based on *A. paludum* females, *P. tigrinaG. gigas* females, and *G. gigas* males when *E* = 10 GPa. Jump performance measured by three variables calculated by the model: takeoff velocity (*A*–*D*), maximum jump height (*E*–*H*), takeoff delay (*I*–*L*). Average empirical values (mass, leg length for each leg section, leg radius, initial height of the body) for each body size class were used to simulate the jumps for each body size class across a wide range of angular velocity of leg rotation (*x* axis). Orange dots represent surface tension jumps, and other dots represent drag-involving jumps. The radius ratio of 5.05, 3.5, 2.24, and 1 (i.e., no bubble situation) are represented as light blue, blue, dark blue, and black dots, respectively. The red-shaded vertical bars represent the ranges of the observed leg angular velocity values ( $\omega_{e}$ ). For smaller species, known to be able to precisely adjust their leg angular velocity in order to perform just under the threshold line (19, 22), a narrow band is additionally marked with darker shade for the range of $\omega_{e}$ values that represent jumps in this optimal situation. The gray-shaded vertical bars represent the range of the hypothetical leg angular velocity ( $\omega_{t}$ ) for *A. paludum* using drag in their jumps, and for the other large species using surface tension jumps. The angular velocity of leg rotation, $\omega_{e}$ , values were determined from slow motion jumping videos as explained in the *SI Appendix*, *Supplementary Materials PART 14* and shown in *SI Appendix*, Table S9. The performance of drag-involving jumps was calculated for three sizes of air bubble surrounding the leg: minimal, maximal, and average. Similar figures for Young’s modulus of 5 and 15 GPa are shown in *SI Appendix*, Figs. S24 and S25.

We used the model to predict jump outcomes (Fig. 5) for body weights and leg lengths corresponding to four size classes of three species of water striders (from the smallest to the largest, consistent with Fig.1): *A. paludum* female, *P. tigrina, G. gigas* females, *G. gigas* males. We used males and females of *G. gigas* separately due to the strong body size dimorphism in this species. *P. tigrina* does not show strong body size dimorphism. We observed females of *A. paludum* as the largest size class among the “typical-sized” water striders. Those predictions were calculated for a wide range of values of the angular velocity of midleg rotation ( ω ; on the horizontal axes in Fig. 5 and See *SI Appendix*, Table S13 for the specific values of parameters used in each simulation), and are shown as either orange dots or dots in one of the four colors (black, dark blue, blue, and light blue) in Fig. 5 representing performance during surface tension and drag-involving jumps, respectively.

These results allow us to compare the theoretically predicted jumping performance (takeoff velocity, takeoff delay, and maximum height) of each size class of water striders (represented by average body size for each class) for various angular midleg velocities, including the velocities actually used by the water striders ( $\omega_{e}$ , observed in precisely digitized jumps of multiple water striders of each species/sex classes, *SI Appendix*, Table S9; marked by vertical red shades in Fig. 5) and those that are only hypothetical/theoretical ( $\omega_{t}$ , marked by vertical gray shades in Fig. 5). This hypothetical angular velocity ( $\omega_{t}$ ) is the one that results in the absence of drag force in large species and results in existence of drag force in *A. paludum*. The ranges of hypothetical angular leg velocities for each of the three large classes (who perform drag-involving jumps) were determined by using the ratio $\omega_{e}/\omega_{c}$ of *A. paludum*, while those for *A. paludum* (who performs surface tension jumps mostly) were determined by using the average ratio $\omega_{e}/\omega_{c}$ of the three large classes (see details in *SI Appendix*, *Supplementary Material PART 14*).

We also calculated predictions using a range of values for Young’s modulus of insect cuticle, E , (Fig. 5 and *SI Appendix*, Figs. S24 and S25), as well as a range of the ratio of the wetted midleg radius with air bubble to the radius without air bubble (black, dark blue, blue, and light blue dots in Fig. 5 and *SI Appendix*, Figs. S24 and S25). Young’s modulus affects the critical angular velocity of the leg rotation, $\omega_{c}$ , but not the general results from the model (compare Fig. 5 and *SI Appendix*, Figs. S24 and S25). The presence and increased size of the air bubble generally improve the performance of drag-involving jumps (as shown in Fig. 5 and *SI Appendix*, Figs. S24 and S25).

### Simulation Predictions for the Larger Water Striders.

**Fig. 6.**
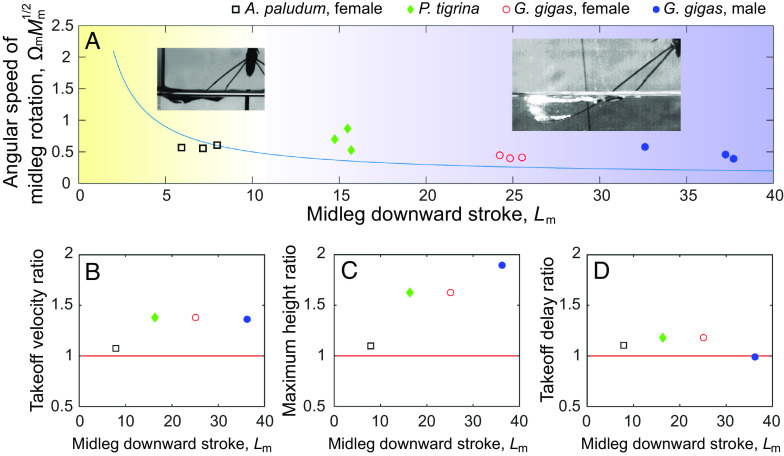
Summary of the results. (*A*) empirically observed jumps in the four classes of water striders (from the smaller to the larger), *A. paludum* females*, P. tigrina*, *G. gigas* females, *G. gigas* males, in the simplified phase diagram based on the original model of surface tension jumping [Yang et al. (19); see Fig. 1] with the theoretical water surface breaking threshold line (blue solid line) to illustrate that large water striders use water surface breaking jumps that involve drag (inset photos). (*B*–*D*) comparison of the theoretically calculated performance in drag involving and surface tension dominant jumps for the four size classes of water striders: the points indicate the estimated ratios calculated by dividing the midpoint of performance (takeoff velocity in *B*, maximum height in *C*, and takeoff delay in *D*; all calculated based on Fig. 5) in the drag-involving jumps by the analogical midpoint of performance in the surface tension jumps. The black empty squares, green filled diamonds, red empty circles, and blue filled circles represent *A. paludum* females, *P. tigrina*, *G. gigas* females, and *G. gigas* males, respectively. In *B*–*D*, the red lines represent a ratio of 1, where the performances of drag-involving jumps and surface tension jumps are equal.

For consistency among Figs. 1, 5, and 6, the model results are arranged from the smallest to the largest body size class in Fig. 5. As we built the model based on the largest water striders, we present the results for *Gigantometra* and *Ptilomera* first, before comparing them with the smaller species (*A. paludum*). The results demonstrate that if the large water striders had used the hypothetical lower angular velocities of midlegs ( $\omega_{t}$ ) than the critical surface-breaking velocity ( $\omega_{c}$ , ~11.3 rad/s, ~15.1 rad/s, and ~27 rad/s for *G. gigas* male and *G. gigas* female, and *P. tigrina*, respectively; marked on *x* axis of Fig. 5 for *E* = 10 GPa) their jumping performance would have been lower than their actual jumping performance involving $\omega_{e}$ . Relatively higher takeoff velocity (Fig. 5 *B*–*D*) and greater jumping height (Fig. 5 *F*–*H*) are likely to contribute to the success in avoiding attacks by underwater predators such as fish that snatch prey from the water surface. While, on average, the predicted takeoff delay across the gray shade appears not that different from the average predicted takeoff delay across the red-shaded band of $\omega_{e}$ (Fig. 5 *J*–*L*), the hypothetical jumps by large water striders just below the critical value, $\omega_{c}$ , may perform better in terms of shorter takeoff delay but then the body velocity and jump height would be lower than in drag-involving jumps.

Fish, in general, can reach speeds of about 1.4 m/s [median for maximal speed from 45 studies on 14 species (33), *SI Appendix*, Fig. S27]. Based on these data, we theoretically estimated that the maximum height of the hypothetical upward “jumps” (into air) by fish in pursuit of escaping (jumping) water strider would range from approximately 50 to 150 mm (lower and upper quartiles in *SI Appendix*, Fig. S27 *B* and *C*). Hence, the large water striders performing surface-breaking (i.e., drag-involving) jumps would be able to jump equal to or faster and/or higher than the fish within a presumably sufficiently short time (takeoff delay approximately up to 100 ms; Fig. 5 *J*–*L*) to escape capture. However, if they had performed surface tension jumps, the takeoff velocities and jump heights would not likely have been sufficient to escape from the fish, especially for the heaviest class (*G. gigas* males; Fig. 5 *D* and *H*). Therefore, we hypothesize that the jumps observed in large water striders produced by midlegs’ angular velocities that lead to surface breaking should help the insects to escape predatory attacks, while the hypothetical surface tension jumps produced by hypothetical (not observed in nature) lower angular velocities of midlegs might put large water striders under more serious risk of predation due to relatively slow speed and low jump height. In the simulation of *P. tigrina*, we found that within a narrow range of ω , the optimal performance for surface tension jumps was equivalent to the performance of drag-involving jumps. However, since *P. tigrina* prefers fast-flowing habitats (34) where the maximum depth of dimple is expected to be shallower than in stagnant water (30), we hypothesize that this peak performance for surface tension jumps may not be achievable in their natural environment.

Finally, the results show that the presence of air bubbles around midlegs improves the performance of drag-involving jumps by enlargement of projected areas of thrusting legs (Fig. 5 and *SI Appendix*, Figs. S24 and S25). In our study, we assumed that this layer of air bubble enhances the drag because it increases the radius of a solid cylinder imitating the midleg in the model. However, the observed air bubble was dragged by the midleg while changing its shape (Fig. 2*B*), and we hypothesize that the air bubble of constantly changing shape may change the leg’s drag coefficient and potentially enhance the drag more than just enlarging the projected area of the thrusting leg.

### Simulation Predictions for the Smaller Water Striders.

Unlike the larger water striders, the smaller water strider species such as *A. paludum* can achieve efficient escape without surface breaking (Fig. 5 *A*, *E*, and *I*; for *E* = 10 GPa), if they are able to precisely adjust the leg velocity to their individual body mass (as suggested earlier in refs. 19 and 22) such that their $\omega_{e}$ values lie just below the critical value, $\omega_{c}$ (dark red shades on the right side of red-shaded vertical band in Fig. 5 *A*, *E*, and *I*). If they used leg angular velocity higher than the body size–specific critical value, the jump performance would become dramatically worse as already described by Yang et al. (19), and confirmed by us via considering drag calculations. This performance decrease cannot be recovered within the expected hypothetical range of ( $\omega_{t}$ , gray vertical shade) by faster leg velocity (even with the maximum volume of air bubble; Fig. 5 *A* and *E*). In order to achieve a performance comparable to the best performance in the surface tension jumps, this smaller species would need to use extremely fast angular leg velocities of 70 to 100 rad/s (Fig. 5 *A*, *E*, and *I*), which may not be easily achievable, or if achievable it may require more energy than the surface tension jumps. Even if they were achievable, they would not provide more protection from predatory attacks because the achievable performance of the hypothetical drag-involving jumps of *A. paludum* (Fig. 5 *A*, *E*, and *I*) is predicted to be lower or not higher than the best performance in the surface tension jumps (i.e., those jumps with the observed leg velocity, $\omega_{e}$ ) that is closer to the critical value, $\omega_{c}$ (dark red shades in Fig. 5 *A*, *E*, and *I*).

We also observed a range of various values of $\omega_{e}$ in individuals of various body sizes (*SI Appendix*, Table S9). Using the lower values of $\omega_{e}$ within this range to theoretically predict jumping performance of a female with an average body mass (48 mg; average mass for *A. paludum* females, *SI Appendix*, Table S3) resulted in a relatively poor performance (left side of the red-shaded vertical band in Fig. 5 *A*, *E*, and *I*) compared to the performance for larger $\omega_{e}$ values, highlighting the importance of leg rotation adjustment to body size for these water striders in performing surface-dominant jumps near the critical value, $\omega_{c}$.

These model predictions allow us to understand why smaller species, who are known to perform near the threshold (19), would not use the drag-involving jumps. Direct empirical observations provide further explanations. In our previous empirical studies (18, 19, 22), we have occasionally observed surface breaking in the smaller species jumping in the laboratory conditions. The breaking occurred in the final moments of jump when the tibia-tarsi section was more-or-less vertically oriented (>45° to horizontal; example in Fig. 2*C1*), and the insect moves upward (Fig. 2*C1*) preventing the immerged leg, including its leg tips, moving downward (i.e., the leg could not create upward drag force, or even might provide downward drag force). Additionally, we noticed that the midlegs of the typical smaller water strider species, such as *A. paludum*, seem not to create pronounced air sheaths in the water presumably due to shorter hairs on the legs (Fig. 2 *D* and *G*), further diminishing the role of drag for powering the jump in these water striders.

### Comparison of the Larger and Smaller Water Striders.

Taken together, our results provide an understanding of why jumping behaviors of the three classes of large water striders with body mass ranging from ~80 to ~500 mg and midleg downstroke ( $L_{m}$ ) ranging from ~15 to ~38 (*G. gigas* males, *G. gigas* females, and *P. tigrina*) do not conform to the relationship between size and leg angular velocity within the surface-tension-dominated mechanism of jumping (Fig. 6*A*), while jumping of *A. paludum* females with body mass of ~40 to ~50 mg and $L_{m}$ of ~6 to ~8 occurs in accordance with the theory of surface tension jumping. According to calculations based on the theoretical model (19), it is possible for large water striders to jump without breaking the water surface if they rotate their legs by 38 to 67% of their current angular velocity (*SI Appendix*, Table S5; using threshold line in Fig. 6*A*). However, the performance of their surface tension jumps would be worse than that of drag-involving jumps (Fig. 6 *B* and *C*), and it would not protect them from attacking predators.

In contrast, one of the largest classes of typical water striders, *A. paludum* females, does not achieve noticeably better jump performance with drag-involving jumps than with surface tension jumps (black square in Fig. 6 *B* and *C* is located near the ratio value of 1). Hence, the shift from surface tension jumps to drag-involving jumps is predicted to occur in the species whose size lies between *A. paludum* and *P. tigrina*, (*SI Appendix*, Fig. S1), i.e., within the range of dimensionless midleg length ( $L_{m}$ ) from ~8 to ~15, corresponding to the midleg length between 26 and 44 mm and body mass between ~50 and ~80 mg (maximal *A. paludum* = 54 mg, minimal *P. tigrina* = 83 mg).

Previous studies (16, 26, 27) have determined that water strider locomotion is characterized by the Weber number of about 0.1, and our data of *A. paludum* female confirm this knowledge (an average value of 0.17 among individuals in Fig. 6*A*). However, our results demonstrate that Weber number can be around 2 for jumps of the large water striders (1.75, 2.91, and 1.55 for *G. gigas* male, *G. gigas* female, and *P.tigrina*, respectively; average values among individuals in Fig. 6*A* and *SI Appendix*, Table S10) indicating that drag plays an important role, similar to fishing spiders galloping and jumping on water (30). Unlike the basilisks (28, 29), this locomotion of large water striders does not include the fast slapping of the water surface, but it includes fast downward expansion of an already existing dimple beyond the point of breaking, leading the capture of air bubbles. Published data on several small water strider species (18, 19, 22, 23), combined with our observations of *A. paludum*, *P. tigrina*, and *G. gigas*, match the model predictions but currently there is not enough information on jumping behavior of a variety of species within Gerridae to fully evaluate the central prediction of the model: Evolutionary transitions from smaller to larger body size along branches of Gerridae phylogenetic tree will be associated with transitions from surface-tension to drag-involving jumps, especially in habitats of high predation risk where achieving sufficiently high jumping performance is important to evade predatory attacks. Future comparative studies of a variety of small and large water strider species should be able to more precisely determine the body size and midleg length at which the transitions occur. The two subfamilies of water striders, Gerrinae and Ptilomerinae, are promising study taxa because of their wide range of species body sizes (*SI Appendix*, Fig. S1*B*) and a variety of the habitats that they use.

### General Conclusions.

In summary, drag-involving jumps allow large water striders to achieve performance that is comparable to the surface tension jumps of the smaller typical water striders, and appears sufficient to evade predatory attacks. Hence, the results suggest that selection for sufficiently fast jumping might have led to a change in the mechanisms of jumping in the large and heavy water striders, leading to evolution of specialized hairs on their midlegs’ tibiae and tarsi that capture air and enhance the drag, which is important for their jumps. The results illustrate a general idea that natural selection for a specific outcome of behavior is influenced by physical constraints in certain habitats, which can break the theoretically expected scaling relationships predicted from the specific biomechanics of the behavior. As a result, a shift to a new mechanism may occur to ensure similar or better behavioral outcomes, such as escape performance from predators, and this mechanism may cause new morphological adaptations and different scaling relationships.

Many of the water strider robots developed thus far are relatively heavy [~0.5 to ~10 g (35–45); except for the ~70-mg jumping robot (18) inspired by the theory for surface tension-dominant jumping (19)] compared to the size range of water striders studied in nature [~10 to ~50 mg (17, 19, 46)]. In a recent study (27), it was shown that utilizing drag can be beneficial for large jumping robots. However, we illustrate here that in nature, adaptive pressure has already optimized the jumping behavior of large-sized water striders by shifting their behavior toward drag utilizing jumps. This highlights the importance of understanding the proximate physical mechanisms and natural selection pressures associated with animal locomotion in designing water walking robots.

## Methods

### Study Species and Locations.

The experiments on *G. gigas* were carried out in Pu Mat National Park, Vietnam. *P. tigrina* jumps were studied at two sites: near the Me Linh Station for Biodiversity (21°23′01.9″N 105°42′44.2″E = Google map: 21.383870, 105.712264;), Vinh Phuc Province, Vietnam, and at the “May waterfalls” (Thac May; 20°21′51.4″N 105°26′51.6″E = Google map: 20.364275, 105.447665), in the vicinity of the Cuc Phuong National Park, Vietnam. *A. paludum* individuals used in research came from water bodies located in and near Seoul, S. Korea.

### Experiments.

Water striders were filmed using Trouble Shooter camera (TS 1000 set to 500 fps) in a 30 × 30 cm^2^ Plexiglas box filled with water. A second camera (Sony SR11) recorded from above simultaneously (*SI Appendix*, *Supplementary Material PART 3*: *SI Appendix*, Fig. S3). Each individual was photographed and weighed immediately after a test (with few exceptions of individuals that escaped before measurements). Additional colored movies were filmed using a Sony RX-III camera. The photos included a ruler and were taken in a manner that allowed for body and length measurements from the photos.

### Digitizing and Analysis.

We chose three best-quality videos of male *G. gigas* for detailed digitization. The videos were digitized manually using MAXTRAQ program (see details in *SI Appendix*, *Supplementary Material PART 6*). Digitization and calculation were repeated 5 times to minimize potential human error and resolution noise. The velocities of the body and legs were based on the differences in positions of digitized points between consecutive frames. As the raw coordinates showed random fluctuations due to the errors in tracking, we used the moving average of three values of three consecutive frames: the preceding frame, the focal frame, and the following frame. The acceleration values were obtained in the same way from the velocity values (moving average of three consecutive values of acceleration). The momentum and force applied to the body were calculated from the velocity, acceleration, and the body mass according to standard formulas.

The jumping of *G. gigas* was divided into three phases. The surface tension phase lasted until the frame when the water surface started breaking under the midleg. The transition phase (marked with the yellow vertical band in Fig. 3 and *SI Appendix*, Figs. S5 and S6) lasted from the first frame with water breaking until the frame before the first frame when midlegs were entirely immersed (and surface tension did not contribute to the jump). The drag phase lasted from the first frame when midlegs were entirely immersed until the body center reached the maximum velocity. Cumulative momentum gained during each of the three jump phases was calculated in each jump. We also determined the moment when the air bubble formed and detached from the midleg. For each frame, we determined the angle between vertical line and hindleg’s as well as midleg’s (left and right leg separately) femur and used these values to extract angular velocity of legs (see details in *SI Appendix*, *Supplementary Materials PART 6*). The hindleg’s maximum dimple depth was also digitized (see details in *SI Appendix*, Fig. S9) because it is crucial in the empirical analyses and in the mathematical model (*SI Appendix*, *Supplementary Materials PARTS 9 and 10*).

The total volume of air bubbles captured around the midleg during the drag phase was calculated by adding the volumes of all air bubbles formed by air detached from the leg during the last stages of the drag phase (n = 14, *SI Appendix*, Table S8). In volume calculations, we used the vertical diameter of each air bubble after its shape stabilized and approximated a sphere.

The dimensionless indices crucial in the mathematical model, the maximal downward reach of legs ( L ) and the combination of leg downward angular speed with the insect mass ( $\Omega M^{1/2}$ ) were calculated for Fig. 6 based on the previous study (19). However, unlike in the original model (19) that used average leg length (from four legs: two hindlegs and two midlegs), we followed the reasoning introduced in the recent model correction (22), which we further modified: we used only the empirically established midleg length (*SI Appendix*, Tables S1–S4) in calculations of those indices (*SI Appendix*, Tables S12 and S13). We did not use hindleg length in the determination of L because their push downward is shallower even in the surface tension jumps (19, 22), and they do not enter deeper into the water in drag-involving jumps (i.e., do not break surface; see *SI Appendix*, Fig. S15).

### Theoretical Model and Simulations of Jumps.

*SI Appendix*, *Supplementary Materials PART 7–11 and 19* contain the detailed presentation of the core mathematical part of the model, and additional details concerning assumptions and parameters based on empirical observations. We assumed that the cuticle of water striders has Young’s modulus similar to that of locusts, reported to be up to ~10 GPa (47, 48). As the modulus of insect cuticles can vary widely (49, 50), We additionally run the model using values of 5 and 15 GPa.

We used the model to theoretically simulate jumps and to predict jump outcomes for body masses and leg lengths corresponding to four size classes based on real water striders from the three study species: (from the largest to the smallest): *G. gigas* males, *G. gigas* females, *P. tigrina, A. paludum* females. We used males and females of *G. gigas* separately due to the strong body size dimorphism in this species, and we used females of *A. paludum* because they represent the largest size from among the typical-sized water striders. Those predictions were calculated for wide ranges of values of the midleg angular velocity (*ω*) covering the surface tension-based and drag-involving jumps and were expressed as three measures of jump performance: takeoff velocity, maximum jump height, and takeoff delay. *SI Appendix*, Table S13 contains the specific values of parameters used in each simulation.

## Supplementary Material

Appendix 01 (PDF)

Dataset S01 (XLSX)

Movie S1. ***Gigantometra gigas* jumping in the natural habitat and the water container and the bubble sheath around the leg**. Movie timeline: 1-3 s. – normal speed (1x); 4-10 s. – slowed down 8x; the clip shows two examples of upward jumps by the giant water strider and landing on the water surface (C0143). The water strider leaves the field of view in the video that was filmed at a closer distance (C0153). Movie timeline: 11-12 s. – normal speed (1x); 13-16 s. – slowed down 10x; the clip shows two examples of two different upward jumps by the giant water strider in the water container in the front (EVT16) and side view (EVT19). Movie timeline: 17-22 s. – slowed down 20x; the clip shows two examples around the leg of jumping water strider (EVT22 (2)) and dead leg striking into the water surface (C0143 dead leg).

Movie S2. ***Ptilomera tigrina* jumping in the water container and the bubble sheath around the leg**. Movie timeline: 1-3 s. – slowed down 4x; 4-6 s. – slowed down 16x; the clip shows two examples of two different upward jumps by the water strider in the water container in the front (C0046) and side view (C0049).

Movie S3. ***Aquarius paludum* jumping in the water container without breaking water surface**. Movie timeline: 1-3 s. – slowed down 4x; 4-6 s. – slowed down 20x; the clip shows an example of an upward jump by the water strider in the water container in the front (P_female_evt26) and side view (P_female_evt25).

## Data Availability

Datasets associated with analyses/figures are located in the supporting information. The Matlab code for the theoretical model is available at Zenodo https://doi.org/10.5281/zenodo.7847879 (51).
