# Supplementary material for: Two different jumping mechanisms of water striders are determined by body size
Source: Proc Natl Acad Sci U S A. 2023 Jul 18;120(30):e2219972120. doi: 10.1073/pnas.2219972120 (PMC10372557; doi:10.1073/pnas.2219972120)
Supplement: Supplementary file 1 — Appendix 01 (PDF) [file pnas.2219972120.sapp.pdf]

PNAS

Supporting Information for

Two different jumping mechanisms of water striders are determined by body size

Woojoo Kim[a1], Juliette Amauger[b1], Jungmoon Ha[a], Thai Pham Hong[c,d], Anh Duc Tran[e], Jae Hong Lee[f], Jinseok Park[a], Piotr G. Jablonski[a,g2], Ho-Young Kim[f,h2], Sang-im Lee[i,j2]

[2] Correspondence to (alphabetically): piotrjab@behecolpiotrsangim.org (P. G. J.), hyk@snu.ac.kr (H.-Y. K.), and sangim@dgist.ac.kr (S.-i.L.)
[1] W.K. and J.A. contributed equally to this work.

This PDF file includes:

Supporting text

Figures S1 to S33

Tables S1 to S13

Legends for Movies S1 to S3

SI References

Affiliations:

[a]Laboratory of Behavioral Ecology and Evolution, School of Biological Sciences, Seoul National University, Seoul 08826, Korea
[b]Laboratoire d'Hydrodynamique de l'X (LadHyX), UMR CNRS 7646, École Polytechnique, 91128 Palaiseau Cedex, France
[c]Mientrung Institute for Scientific Research, Vietnam National Museum of Nature, Vietnam Academy of Science and Technology, 49000 Hue, Vietnam
[d]Graduate University of Science and Technology, Vietnam Academy of Science and Technology, 100000 Hanoi, Vietnam
[e]Department of Applied Zoology, Faculty of Biology, University of Science, Vietnam National University, 100000 Hanoi, Vietnam
[f]Department of Mechanical Engineering, Seoul National University, Seoul 08826, Korea
[g]Museum and Institute of Zoology, Polish Academy of Sciences, 00-679 Warsaw, Poland
[h]Institute of Advanced Machines and Design, Seoul National University, Seoul 08826, Korea
[i]Laboratory of Integrative Animal Ecology, Department of New Biology, Daegu Gyeongbuk Institute of Science & Technology, Daegu 42988, Korea
[j]New Biology Research Center, Daegu Gyeongbuk Institute of Science & Technology, Daegu 42988, Korea

The supplementary material is listed in the order of the contents.

Supplementary Materials PART 1. Morphology of the study species: *Gigantometra gigas*, *Ptilomera tigrina*, and *Aquarius paludum*.
p. 3-6
Supplementary Materials PART 2: Description of the Supplementary Movies and links to additional movies deposited to Wikimedia.
p. 7-8
Supplementary Materials PART 3: Jumps of large-sized water striders.
p. 9-10
Supplementary Materials PART 4: Duration of jump phases in *Gigantometra gigas.*
p. 11
Supplementary Materials PART 5: Additional Results from the detailed analyses of jumps.
p. 12-13
Supplementary Materials PART 6: Assumptions and methods of digitizing.
p. 14-16
Supplementary Materials PART 7: Extraction of information from empirical measurements for the model's assumptions about the functional/effective radius of the wetted leg.
p. 17-18
Supplementary Materials PART 8: Observations of midleg dimple breaking and the role of the breaking process in the simulation model.
p. 19-20
Supplementary Materials PART 9: Observations of hindlegs in the jumps of *Gigantometra gigas*.
p. 21
Supplementary Materials PART 10: Hindleg's constant depth, h_{hm}.
p. 22
Supplementary Materials PART 11: Maximum jumping height calculation.
p. 22
Supplementary Materials PART 12: Additional empirical results for *G. gigas* females and *P. tigrina.*
p. 23-24
Supplementary Materials PART 13: Model validation - comparison with empirical observations.
p. 25
Supplementary Materials PART 14: Validation of the use of empirical constant angular velocity of the middle leg rotation, ω_e.
p. 26-29
Supplementary Materials PART 15: Additional simulation results for different Young's modulus of insect cuticle, E.
p. 30-31
Supplementary Materials PART 16: Examples from the model simulations of the repeated "cycle" of dimple depth and body velocity fluctuations.
p. 32
Supplementary Materials PART 17: Maximum jumping performance of fish.
p. 33
Supplementary Materials PART 18: Weber number of the study species: *Gigantometra gigas*, *Ptilomera tigrina*, and *Aquarius paludum.*
p. 33
Supplementary Materials PART 19: **Detailed description of the mathematical model of jumping.**
p. 34-44

Supplementary Materials PART 1. Morphology of the study species: *Gigantometra gigas*, *Ptilomera tigrina*, and *Aquarius paludum*.

Table S1. Morphology of the giant water striders, *Gigantometra gigas*, from the study site in Pu Mat National Park, Vietnam.

Variable	Males	Females
Sample size (nr of individuals)	16	9
Mass (mean ± SD, min-max; mg)	414 ± 59, 316-511	265 ± 40, 217-318
Body length (cm)	3.52 ± 0.20	3.39 ± 0.32
FRONT LEGS:		
Tibia Thickness (mm)	0.41 ± 0.05	0.39 ± 0.04
Femur length (cm)	1.11 ± 0.10	1.00 ± 0.10
Tibia length (cm)	0.89 ± 0.07	0.75 ± 0.06
Tarsus length (cm)	0.27 ± 0.03	0.23 ± 0.03
Total leg length (cm)	2.27 ± 0.17	1.99 ± 0.15
MIDLEGS:		
Tibia Thickness (mm)	0.46 ± 0.08	0.39 ± 0.04
Femur length (cm)	4.84 ± 0.45	3.32 ± 0.28
Maximum wetted leg length (Tibia + tarsus length; cm)	5.35 ± 0.60	3.85 ± 0.24
Total leg length (cm)	10.19 ± 1.04	7.17 ± 0.43
HINDLEGS:		
Tibia Thickness (mm)	0.56 ± 0.09	0.40 ± 0.05
Femur length (cm)	4.95 ± 0.69	3.21 ± 0.11
Maximum wetted leg length (Tibia + tarsus length; cm)	7.35 ± 1.16	4.06 ± 0.50
Total leg length (cm)	12.30 ± 1.25	7.26 ± 0.57

Table S2. Morphology of the three individuals of *Gigantometra gigas* for whom the jumps were fully analyzed. Thickness of the tibia was measured near the femur/tibia joint as a diameter of leg measured along the horizontal line (parallel to the water surface in the normal position of a leg of a water strider standing on the water surface). The thickness is used in the model to calculate drag force after correction for the presence of air bubble that surrounds the leg moving in the water (see calculations in Supplementary Materials PART 7)

Clip name	Mass (mg)	Middle leg			Hind leg	
		Basal tibia thickness (mm)	Femur length (cm)	Constant wetted length: *tibia* + *tarsus* (cm)	Femur length (cm)	Constant wetted length *tibia* + *tarsus* (cm)
EVT16	483	0.455	4.809	5.460	4.742	7.948
EVT05 (2)	375	0.390	4.286	4.578	4.274	6.336
EVT41	325	0.475	4.913	5.405	4.824	7.717

Table S3. Morphology of *Ptilomera tigrina* from two study sites: the Melinh Station for Biodiversity, Vinh Phuc Province, Vietnam, and at the “May waterfalls” (Thac May) of the Cuc Phuong National Park, Vietnam; and morphology of *Aquarius paludum* females from Seoul, South Korea.

Variable	*Ptilomera tigrina*	*Aquarius paludum*
Sample size (nr of individuals)	18	8
Mass (mean ± SD, min-max; mg)	115 ± 22, 83-144	48 ± 4, 43-54
Body length (cm)	1.72 ± 0.08	1.55 ± 0.06
MIDLEGS:		
Tibia thickness (mm)	0.31 ± 0.03	0.18 ± 0.02
Femur length (cm)	2.40 ± 0.16	1.15 ± 0.10
Maximum wetted leg length (Tibia + tarsus length; cm)	2.39 ± 0.16	1.34 ± 0.07
Total leg length (cm)	4.79 ± 0.30	2.50 ± 0.16
HINDLEGS:		
Femur length (cm)	2.76 ± 0.23	1.21 ± 0.08
Maximum wetted leg length (Tibia + tarsus length; cm)	1.91 ± 0.19	0.95 ± 0.07
Total leg length (cm)	4.66 ± 0.42	2.16 ± 0.14

Table S4. The midleg downward stroke, L_m, the dimensionless angular velocity of middle leg rotation of a jump, Ω_m, and the dimensionless index of insect body mass with respect to the middle leg, M_m, were calculated according to the following formula from Yang et al. (1), but modified to focus on the midleg, as $L_m = \Delta l_l/l_c$, $\Omega_\mathrm{m} = \omega_e(l_c/g)^{1/2}$, $M_m = m/(\rho l_c^2 C_{m0} l_m)$, because we observed that hindleg does not penetrate the water surface (see details in Supplementary Material PART 8, 19). The summary of these data is shown in Fig. 6.

Parameter/ variable (unit)	*G. gigas* male			*G. gigas* female			*P. tigrina*			*A. paludum* female		
	EVT05 (2)	EVT16	EVT41	EVT28	EVT33	EVT35	C0046	C0049	C0066	P_Female _evt25	P_Female _evt32	P_Female _evt33
m (kg)	374.76e-6	483.23e-6	325.41e-6	305.67e-6	226.81e-6	226.81e-6	134e-6	134e-6	123e-6	48.5e-06	42.6e-06	42.6e-06
y_i (m)	0.00017	0.00165	0.00088	0.00333	0.00435	0.00274	0.00271	0.00473	0.00806	0.00271	0.00473	0.00806
l_l (m)	88.64e-3	102.69e-3	103.17e-3	72.59e-3	70.13e-3	70.13e-3	44.72e-3	44.72e-3	50.63e-3	0.02434	0.02413	0.02413
l_m (m)	45.78e-3	54.60e-3	54.05e-3	39.80e-3	38.87e-3	38.87e-3	22.70e-3	22.70e-3	25.56e-3	13.56e-3	13.06e-3	13.06e-3
ω_e	20	15	16	16	19	17	41	33	29	39	40	41
L_m	32.604	37.240	37.701	25.527	24.240	24.836	15.481	14.740	15.688	7.970	7.151	5.921
Ω_m	0.333	0.250	0.266	0.266	0.316	0.283	0.682	0.549	0.483	0.649	0.666	0.682
M_m	3.029	3.369	2.169	2.388	1.993	1.993	1.624	1.624	1.190	0.874	0.696	0.696
$\Omega_\mathrm{m} M_m^{1/2}$	0.579	0.458	0.392	0.411	0.446	0.399	0.869	0.700	0.526	0.607	0.555	0.569

Table S5. Calculations of theoretical threshold conditions for the large water striders, assuming that the surface-tension-dominant mechanism applies to the jumping by the large water striders (in Table S4). The table shows the predictions of the theoretical critical angular leg velocity values for the empirically observed body masses.

Species	Observed mass (mg)	Observed angular velocity (rad/s)	Theoretical critical angular velocity (rad/s)	Angular velocity ratio (theoretical /observed)
G. gigas male	483	15	6.79	0.45
G. gigas male	375	20	7.69	0.38
G. gigas male	325	16	8.41	0.53
G. gigas female	306	16	9.98	0.62
G. gigas female	227	19	11.28	0.59
G. gigas female	227	17	11.11	0.65
P. tigrina	134	41	16.90	0.41
P. tigrina	134	33	17.51	0.53
P. tigrina	123	29	19.56	0.67

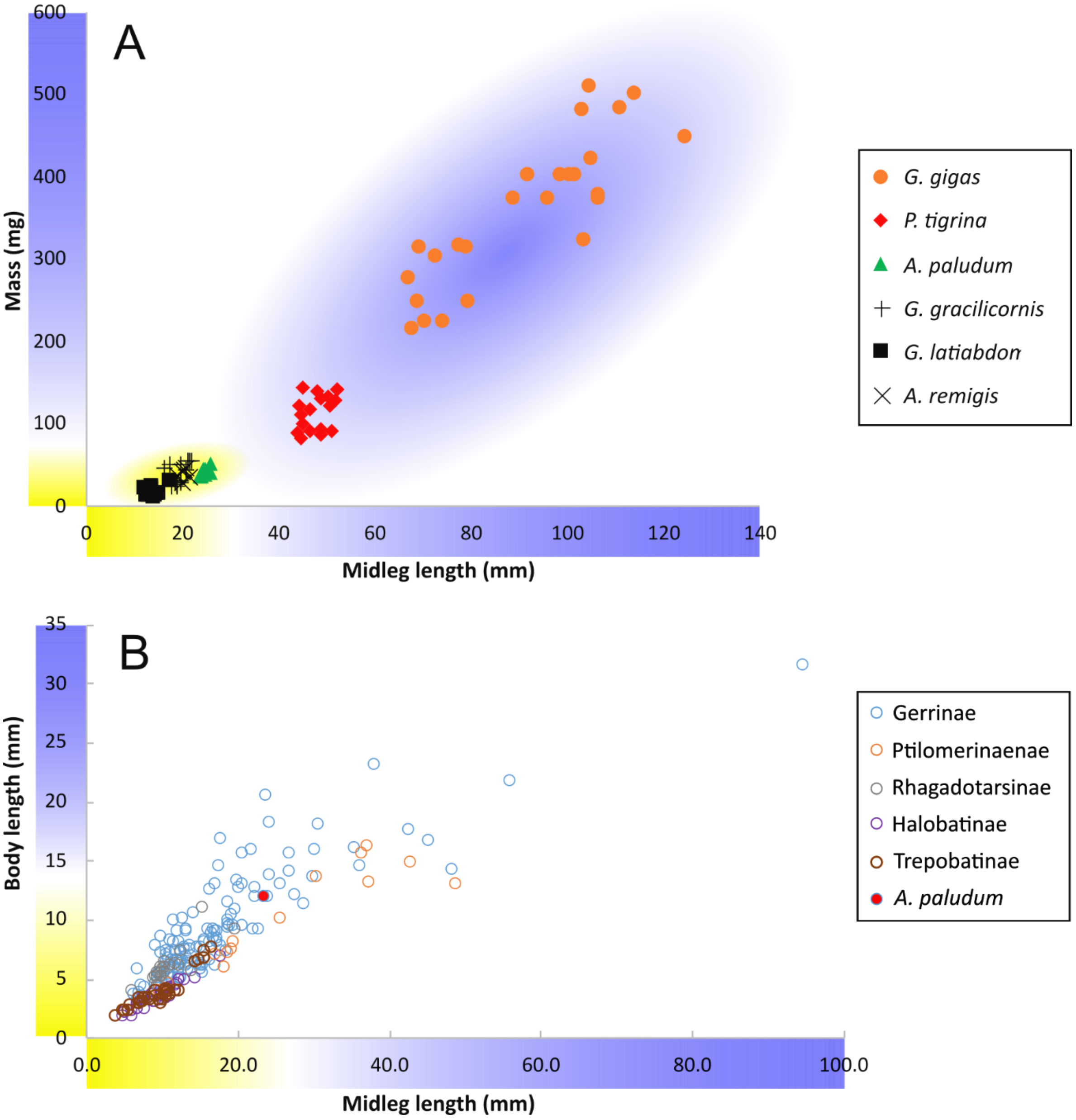

Fig. S1. Relationship between body size and midleg length in the previously studied (yellow ranges on axes) and the unstudied (blue ranges on axes) species of Gerridae divided into 5 subfamilies. (*A*) the absolute midleg length (mm; the variable crucial for empirical biologists and directly related to the dimensionless downward stroke L, the key variable crucial in the theoretical hydrodynamic model of jumping and shown on the horizontal axis in Fig. 1) and the species body mass (empirical variable related to the dimensionless body mass *M*, contributing to the index of angular velocity of midleg rotation, $\Omega M^{1/2}$, which is the key variable the theoretical hydrodynamic model of jumping and is shown on the vertical axis in Fig. 1) for several species from the "typical" water striders (measured in this study: *Gerris latiabdominis, G. gracilicornis, Aquarius remigis, A. paludum*), and from the two large species that have rarely been studied before and were measured here (*Ptilomera tigrina* and *Gigantometra gigas*). (*B*) Reconstructed data from Table 16 in Matsuda 1960 (2). Matsuda states the unit converting rule on page 32: "In table 16, 82 units are equal to 10 mm. For those values with asterisks, 173.7 units are equal to 10 mm." However, in comparison with our measurement data, the rule seems clearly to be a mistake. Therefore, we used the converting rule where '173.7 units are equal to 10 mm', which leads to results consistent with our data on leg and body lengths directly measured by us from specimens. Yellow-shaded area indicates the range of body masses and leg lengths of small and medium sized Palearctic and Nearctic water striders that have typically been studied in the past. Blue-shaded area indicates body and leg lengths that have not been studied earlier. The data point for *A. paludum* is highlighted to indicate the largest of the species (belongs to Gerrinae) studied that uses surface-tension jump.

Supplementary Materials PART 2: Description of the Supplementary Movies and links to additional movies deposited to Wikimedia.

Description of the content of the supplementary video clips with the article:

Supplementary Movie 1 – ***Gigantometra gigas*** **jumping in the natural habitat and the water container and the bubble sheath around the leg.** Movie timeline: 1-3 s. – normal speed (1x); 4-10 s. – slowed down 8x; the clip shows two examples of upward jumps by the giant water strider and landing on the water surface (C0143). The water strider leaves the field of view in the video that was filmed at a closer distance (C0153). Movie timeline: 11-12 s. – normal speed (1x); 13-16 s. – slowed down 10x; the clip shows two examples of two different upward jumps by the giant water strider in the water container in the front (EVT16) and side view (EVT19). Movie timeline: 17-22 s. – slowed down 20x; the clip shows two examples around the leg of jumping water strider (EVT22 (2)) and dead leg striking into the water surface (C0143 dead leg).

Supplementary Movie 2 – ***Ptilomera tigrina*** **jumping in the water container and the bubble sheath around the leg.** Movie timeline: 1-3 s. – slowed down 4x; 4-6 s. – slowed down 16x; the clip shows two examples of two different upward jumps by the water strider in the water container in the front (C0046) and side view (C0049).

Supplementary Movie 3 – ***Aquarius paludum*** **jumping in the water container without breaking water surface.** Movie timeline: 1-3 s. – slowed down 4x; 4-6 s. – slowed down 20x; the clip shows an example of an upward jump by the water strider in the water container in the front (P_female_evt26) and side view (P_female_evt25).

Description of the content of the additional video clips available at Wikimedia and You tube (with links):

NATURAL HABITAT JUMP1.mp4. The clip shows an example of an upward jump by the giant water strider. The movements are slowed down (0.0375 normal speed). The second smaller water strider jumping belongs to the genus *Ptilomera*. *The movie (C0143) was captured in the field at 239.76fps and saved in the standard format of 29.97fps, which was additionally slowed down to 30% of playback speed.*
Wikimedia:
https://commons.wikimedia.org/wiki/File:Gigantometra_gigas_upward_jump_in_Natural_Habitat_Pumat_National_Park_Jump_1.webm
Youtube: https://youtu.be/2EuG5vT4YHs

NATURAL HABITAT JUMP 2.mp4. The clip shows an example of an upward jump by the giant water strider. The movements are slowed down (0.125 normal speed). The giant water strider enters the field of view from the left. The smaller water strider on the right belongs to the genus *Ptilomera*. *The movie (C0153) was captured in the field at 239.76fps and saved in the standard format of 29.97fps, resulting in the slowdown of 0.125 relative to the normal speed.*
Wikimedia: https://commons.wikimedia.org/wiki/File:The_giant_water_strider_(Gigantometra_gigas;_Gerridae)_Natural_Habitat_Jump_2.webm
Youtube: https://youtu.be/jGN1gJBlk5k

NATURAL HABITAT JUMP3.mp4. The clip shows an example of an upward jump by the giant water strider. The movements are slowed down (0.0375 normal speed). The second smaller water strider jumping belongs to the genus *Ptilomera*. *The movie (C0027) was captured in the field at 479.52 fps and saved in the standard format of 29.97fps, resulting in the slowdown of 0.0625 relative to the normal speed.*
Wikimedia: https://commons.wikimedia.org/wiki/File:Gigantometra_gigas_(Gerridae)_Natural_Habitat_Jump_3.webm
Youtube: https://youtu.be/zyW-eV9kxs8

NATURAL HABITAT JUMP4.mp4. The clip shows an example of an upward jump by the giant water strider filmed at a closer distance. The water strider leaves the field of view. The movements are slowed down (0.125 normal speed). The giant water strider enters the field of view from the left. *The movie (C0148) was captured in the field at 239.76fps and saved in the standard format of 29.97fps, resulting in the slowdown of 0.125 relative to the normal speed.*
Wikimedia: https://commons.wikimedia.org/wiki/File:Gigantometra_gigas_(Gerridae)_Natural_Habitat_Jump_4.webm
Youtube: https://youtu.be/tCtFqlHHisU

NATURAL HABITAT JUMP5.mp4. The clip shows a close-up on the water surface under the water strider legs. The movements are slowed down (0.01875 normal speed). At the end of the clip, several small bubbles of air, which was originally wrapped around midlegs during the drag phase of the jump, “pop-up” on the water surface after being dis-attached from the legs (visible in the supplementary video “JUMP IN THE BOX.mp4”). *The movie (C0086) was captured in the field at 959.04 fps and saved in the format of 59.94 fps, which was additionally slowed down to 30%.*
Wikimedia: https://commons.wikimedia.org/wiki/File:Gigantometra_gigas_(Gerridae)_Natural_Habitat_Jump_5.webm
Youtube: https://youtu.be/bbwkCEwDtrA

JUMP IN THE TANK 1.AVI. The clip shows an example of an upward jump by the giant water strider filmed by Trouble Shooter camera (TS1000) with 500 fps. This is one of the three movies analyzed (*EVT16*).

Wikimedia: *https://commons.wikimedia.org/wiki/File:Gigantometra_gigas_(Gerridae)_JUMP_IN_THE_TANK_1.webm*
https://commons.wikimedia.org/wiki/File:Gigantometra_gigas_(Gerridae)_Jump_in_Tank_1_annotated_EVT16_50%25.webm
Youtube: *https://youtu.be/wSd5EKYdPi8*

JUMP IN THE TANK 2.mp4. This clip shows a close up of the midlegs moving downward and surrounded by air bubble caught in the hair around the leg. Eventually, the legs are quickly mowing upward and leave the air bubble, which forms air bubbles that travel slowly upwards towards the surface. This clip (*EVT22 (2) ind20*) was filmed by Trouble Shooter camera (TS1000) at 500 fps.
Wikimedia: *https://commons.wikimedia.org/wiki/File:Gigantometra_gigas_(Gerridae)_JUMP_IN_THE_TANK_2_70PERC.webm*
Youtube: *https://youtu.be/ReE8NQhjo_4*

JUMP IN THE TANK 3.mp4. This clip shows nearly symmetrical movements downwards of midlegs. The movements are slowed down (0.03125 normal speed). The air released from the legs under water creates small air bubbles that travel slowly upwards towards the surface. *The movie (C0041) was captured with Sony RX10-III at 959.04 fps and saved in the format of 59.94 fps, which was additionally slowed down to 50%.*
Wikimedia: *https://commons.wikimedia.org/wiki/File:Gigantometra_gigas_(Gerridae)_JUMP_IN_THE_TANK_3.webm*
Youtube: *https://youtu.be/DagN4SMZOgc*

JUMP IN THE TANK 4.mp4. This clip shows a close-up on legs seen approximately from the side to illustrate that during fast leg downward movements the air bubble around the legs is extended along the direction of leg movements (also seen in the two other clips: JUMP IN THE TANK 2 and DEAD LEG DOWNWARD MOVE. Therefore we could imagine that a cross-section of leg including the air bubble may resemble an irregular ellipse rather than a circle. The drag force is proportional to the effective leg diameter, which is a function of the diameter of the leg plus the thickness of the air measured during downward movement in the plane perpendicular to the leg downward movement (see Supplementary Materials PART 7 for more details on how this aspect was simplified in the theoretical model). The movements are slowed down (0.015625 normal speed). *The movie (C0046) was captured with Sony RX10-III at 959.04 fps and saved in the format of 59.94 fps, which was additionally slowed down to 25%.*
Wikimedia: *https://commons.wikimedia.org/wiki/File:Gigantometra_gigas_(Gerridae)_JUMP_IN_THE_TANK_4.webm*
Youtube: *https://youtu.be/DagN4SMZOgc*

DEAD LEG DOWNWARD MOVE.mp4. This clip shows how the air bubble is created around the midleg during fast downward movements in the water. The movements are slowed down (0.03125 normal speed). *The movie (C0143) was captured in the field at 959.04 fps and saved in the format of 59.94 fps, which was additionally slowed down to 50%.*
Wikimedia: *https://commons.wikimedia.org/wiki/File:Gigantometra_gigas_(Gerridae)_DEAD_LEG_DOWNWARD_MOVE.webm*
Youtube: *https://youtube.com/shorts/-f6E80VricM?feature=share*

JUMPING OF *AQUARIUS PALUDUM*:
Near-vertical jumps of water strider (*A. paludum*) slow motion. - The video clip shows a slow motion of a water strider jumping upwards. Note the “dimples” under the legs and how they increase in depth while the legs push down and “bend” the water surface without breaking it. The model in *Nat Comm* **7,** 13698 (2016) https://doi.org/10.1038/ncomms13698 focuses on this ability of insects to jump upward without breaking of the water surface. It shows that this ability results in maximization of the jump speed and minimization of the latency between the jump initiation and leaving the water surface in the response to attacking predators.
Wikimedia: *Water-striders-adjust-leg-movement-speed-to-optimize-takeoff-velocity-for-their-morphology-ncomms13698-s2.ogv*
Youtube: *https://youtu.be/8sjSmX5pNw8*

Water strider *A. paludum* jump side view. - Slow motion video of the water strider *Aquarius paludum* jumping on the water surface. Side view reveals the backward leg movements. The speed is approximately 0.006 of normal speed.
Wikimedia: *https://commons.wikimedia.org/wiki/File:Water_strider_A_paludum_jump_side_view.webm*
Youtube: *https://youtu.be/cDwGRyFiNoM*

Waterstrider *A. paludum* jump frontal view. - Slow motion video of the water strider *Aquarius paludum* jumping on the water surface. Frontal view reveals the inward leg movements. The speed is approximately 0.006 of normal speed.
Wikimedia: *https://commons.wikimedia.org/wiki/File:Waterstrider_A_paludum_jump_frontal_view.webm*
Youtube: *https://youtu.be/GLy7Obl6jLc*

Water strider *A. paludum* jump on solid substrate. - This video of a water strider jumping on a solid substrate illustrates that the leg movements are composed of two phases. First phase comprises downward movement against the substrate surface (normally surface of water) dominates. The second phase comprises mostly horizontal movements: first backwards then inwards. When this happens on water, where each leg creates a dimple, the leg movements are associated with the dimple shifts backwards and then inwards. The speed is approximately 0.006 of normal speed.
Wikimedia: *https://commons.wikimedia.org/wiki/File:Water_strider_A_paludum_jump_on_solid_substrate.webm*
Youtube: *https://youtu.be/4Sr0im-umSU*

Supplementary Materials PART 3: Jumps of large-sized water striders.

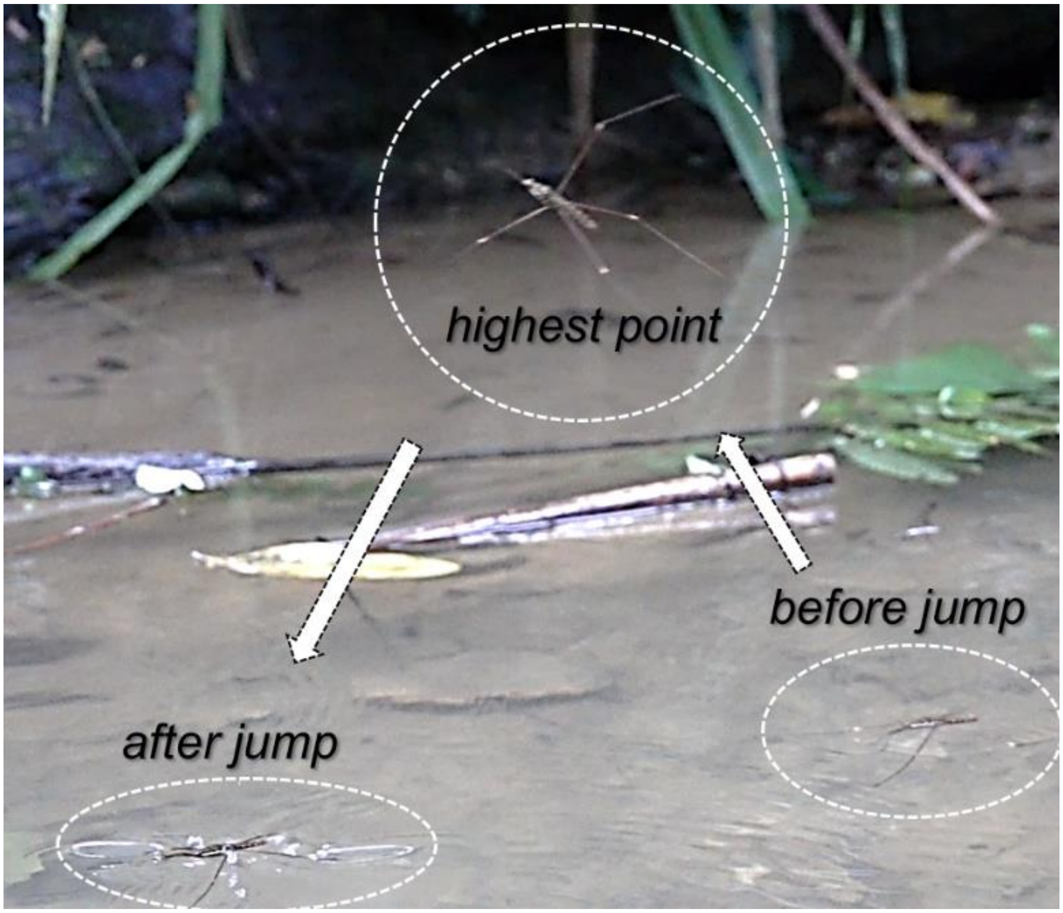

Fig. S2. Jumping in the natural habitat. An example of a record of one jump in the natural habitat. Three frames are put together here and photo-edited to represent the moment just before jump, the moment of reaching the highest point, and the moment right after landing on the water surface again. In the natural habitat, jumps of the giant water strider were triggered by imitating attacks from under the water surface using long bent sticks, or by creating a very fast visual stimulus by waving a sheet of paper in the visual field of the water strider. High speed movies were shot using Sony RX10-III. See details in Supplementary Movie 1.

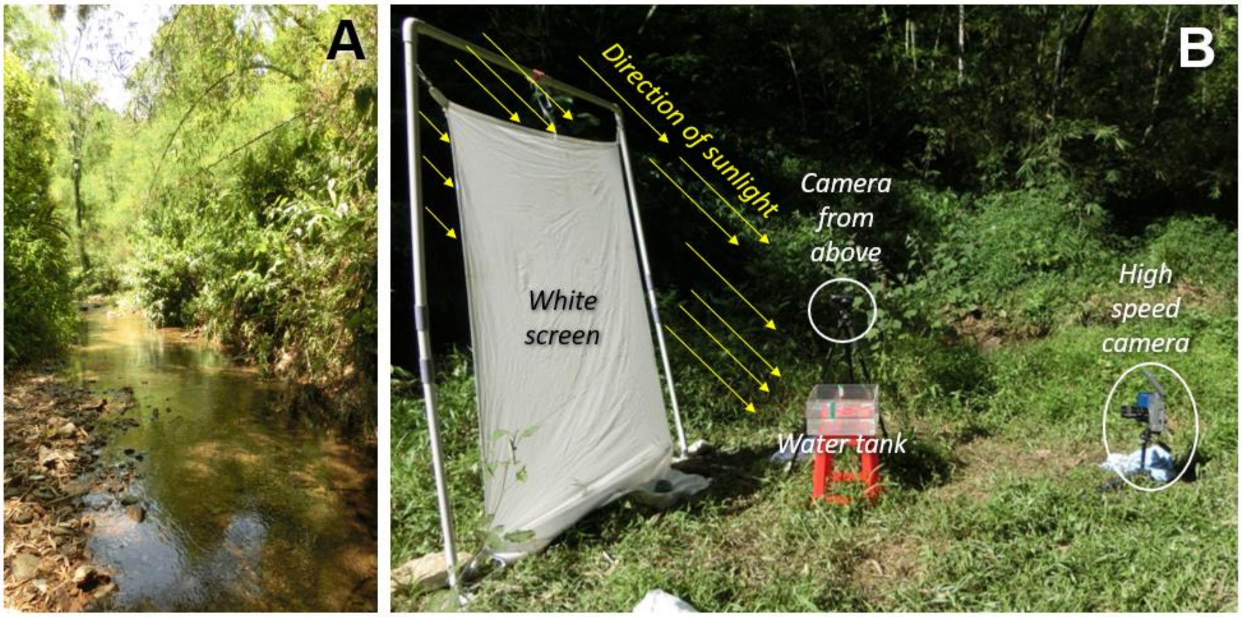

Fig. S3. Field methods. (*A*) Study site in the natural habitat of the giant water strider in the Pumat National Park area. (*B*) Experimental setup used in the field to obtain high-speed movies of jumping giant water striders. A water strider was put in a transparent water tank. Jumping was triggered by poking the insect gently from under water using a hook-shaped wire. Battery-operated high-speed camera (TS 1000) was used to film the jump, while standard camcorder filmed it from above to provide information about distance of the insect to the front wall of the tank (this distance was crucial to translate pixel coordinates into centimeters). A white sheet of fabric spread behind the water tank appropriately located relative to the direction of sun light was used as a background. For some jumps, Sony RX10-III was used instead of the TS 1000.

In the water container, the slow-motion movies used for the detailed analyses have been shot using Trouble Shooter camera (TS 1000) set to film at 500 fps. The set-up is shown in Fig. S3. This setup requires two people for efficient work: one operates the high-speed camera, and the second person handles the water striders and triggers the jumps. Sometimes the water striders used their wings to escape from the water tank and these escapes were not analyzed.

Table S6. Observed number of water surface breaking by midlegs during jumping by the large-sized water striders: *G. gigas* and *P. tigrina*. Breaking was defined as breaking with at least one leg by assuming that in asymmetric jumps the other has lighter load.

	G. gigas	*P. tigrina*	sum
Breaking occurred	57	21	78
No breaking	0	2	2
sum	57	23	80

Supplementary Materials PART 4: Duration of jump phases in *Gigantometra gigas*

The surface tension phase lasts for ~12-44 ms (27±7 ms for a single leg, n = 15, mean±SD, Table S7; ~ 26 ms in Fig. 3; ~22 ms in Fig. S5; ~12 ms in Fig. S6 where the legs are relatively non-synchronized and the second leg starts breaking the surface after ~22 ms). The transition phase lasts ~4-28 ms (12±5 ms, for a single leg, n = 15, Table S7; ~16 ms in Fig. 3; ~18 ms in Fig. S5; ~28 ms in Fig. S6, where it is relatively long because the two legs are not well synchronized). The drag phase lasts ~14-22 ms (15±6, for a single leg, n = 15, Table S7; ~22 ms in Fig. 3; ~14 ms in Fig. S5; ~22 ms in Fig. S6). The duration from the breaking to the detachment of the air bubble lasts 10-32 ms (23±7, for a single leg, n = 15, Table S7; marked with blue-shaded vertical bands in Fig. 3; Fig. S5, S6).

Table S7. Duration of jump phases of *G. gigas* recorded in the water container at the field site. "Surface tension phase" lasts from the start of the jump until surface breaking starts. "Transition phase" lasts from the moment when the first point of surface breaking is detected along one of the two midlegs until the moment when both midlegs fully break the surface. "Drag phase" lasts from the end of the "transition phase" until both midlegs have reached or passed through the deepest point (i.e., none of the two midlegs moved downward anymore). Bubble attached phase is from the start of surface breaking until the air bubble is completely detached from the leg and floats upwards in the form of bubbles. Bubble attached phase is usually included in the "After breaking phase". "After breaking phase" is from the start of surface breaking until the midleg reaches the deepest point (hence it is a sum of "Transition phase + Drag phase"). See also Figure S4 based on these data.

Video (leg)	Surface tension phase (ms)	Transition phase (ms)	Drag phase (ms)	Bubble attached phase (ms)	After breaking (ms)
EVT05 (2) left	22	12	16	32	28
EVT05 (2) right	30	10	12	10	22
EVT16 left	28	14	14	24	28
EVT16 right	26	8	14	36	22
EVT41 left	22	18	16	22	34
EVT41 right	12	14	32	28	46
EVT12	26	20	20	28	40
EVT14	26	10	16	22	26
EVT28	30	8	6	16	14
EVT33	24	14	12	20	26
EVT35	32	6	12	18	18
EVT45	28	6	18	28	24
EVT47	28	16	12	16	28
EVT65	44	4	12	18	16
EVT75	28	14	18	24	32
**Mean (±s.d.)**	**27.1 (±6.7)**	**11.6 (±4.7)**	**15.3 (±5.7)**	**22.8 (±6.8)**	**26.9 (±8.6)**

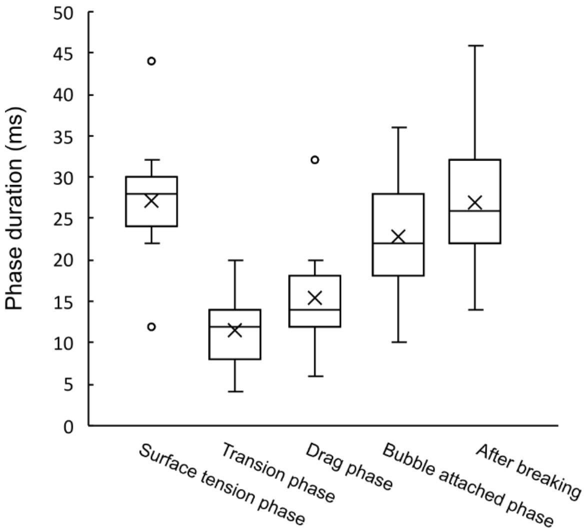

Fig. S4. Box-whisker plots of phase durations (ms). Duration of each phase of the jumps of *G. gigas* in the water container. Surface tension phase is from start of the jump until surface breaking starts. Transition phase is from the start of surface breaking until end of surface breaking. Drag phase is from end of surface breaking until leg reaches the deepest point (i.e., leg stops moving downward). Bubble attached phase is from the start of surface breaking until the air bubble is completely detached from the leg and floats upwards in the form of bubbles. Bubble attached phase is usually included in After breaking phase. After breaking phase is from the start of surface breaking until leg reaches the deepest point (hence it is a sum of "Transition phase + Drag phase"). The data are in Table S7.

Supplementary Materials PART 5: Additional Results from the detailed analyses of jumps

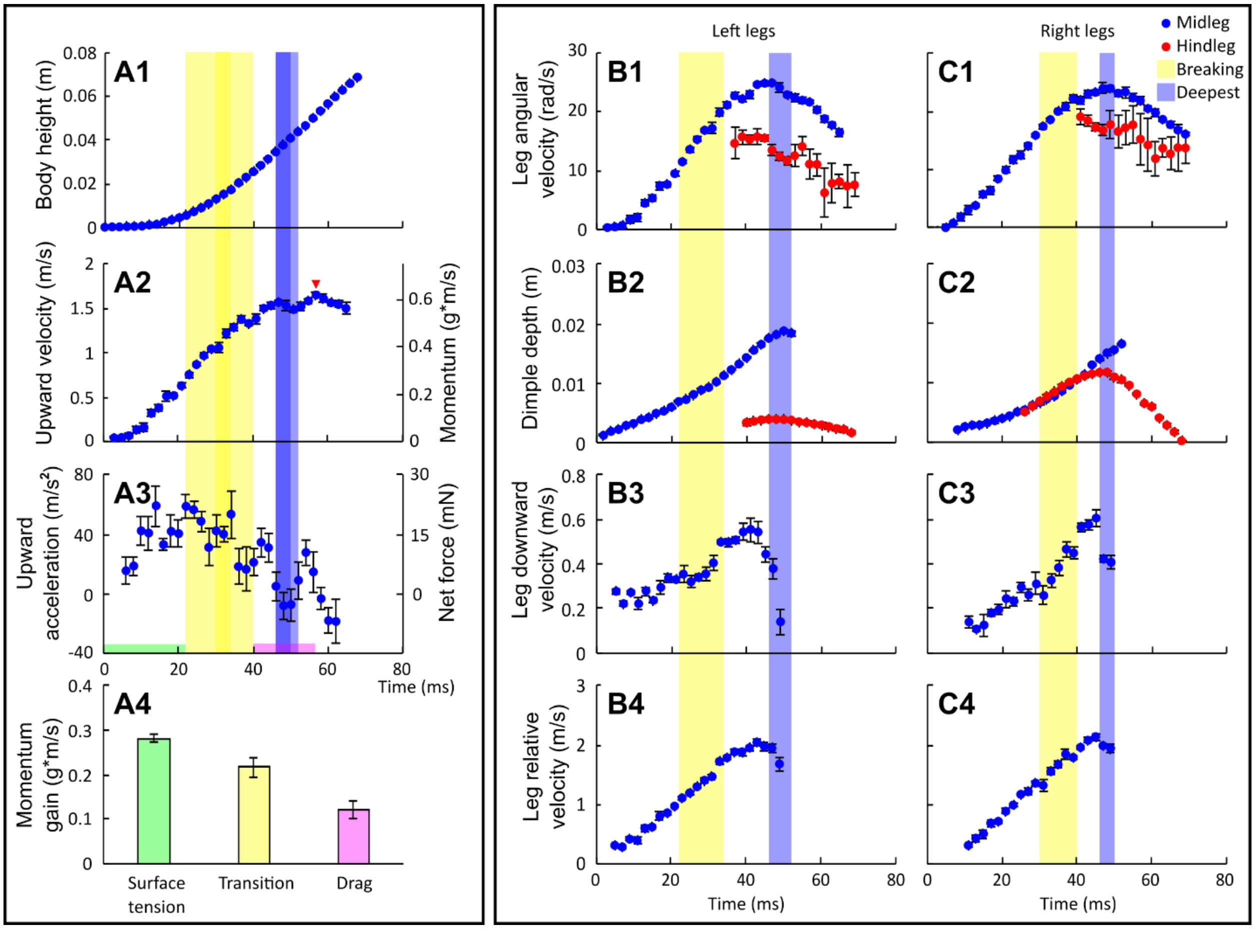

Fig. S5. Kinematics and dynamics of the jumping on water by the giant water strider, *Gigantometra gigas*. Results from analysis of a jump “EVT05 (2)”. (*A*) variables obtained from the body movement: changes of body height above the water surface (*A*1), body velocity (*A*2) and body acceleration (*A*3) during the jump. Right side axes in *A*2 and *A*3 indicated the changes of body momentum (*A*2) and net force (*A*3) during the jump calculated from the body movement and body mass. (*A*4) shows the comparison between the values of momentum gained during the three phases of jump: the surface tension phase (green), the transition phase (yellow) and the drag phase (purple). (*B*) and (*C*) contain variables concerning movements of the left (*B*) and right (*C*) midlegs (blue circles) and hindlegs (red circles in *B*1, *B*2, *C*1, *C*2): angular downward velocity (*B*1, *C*1), depth (*B*2, *C*2), downward velocity (*B*3, *C*3) and downward velocity relative to the body position (*B*4, *C*4). Yellow background indicates the transition phase when surface is breaking. Blue background indicates the bubble detaching phase duration of each leg. Red triangle in *A*2 indicates the moment of maximal body velocity.

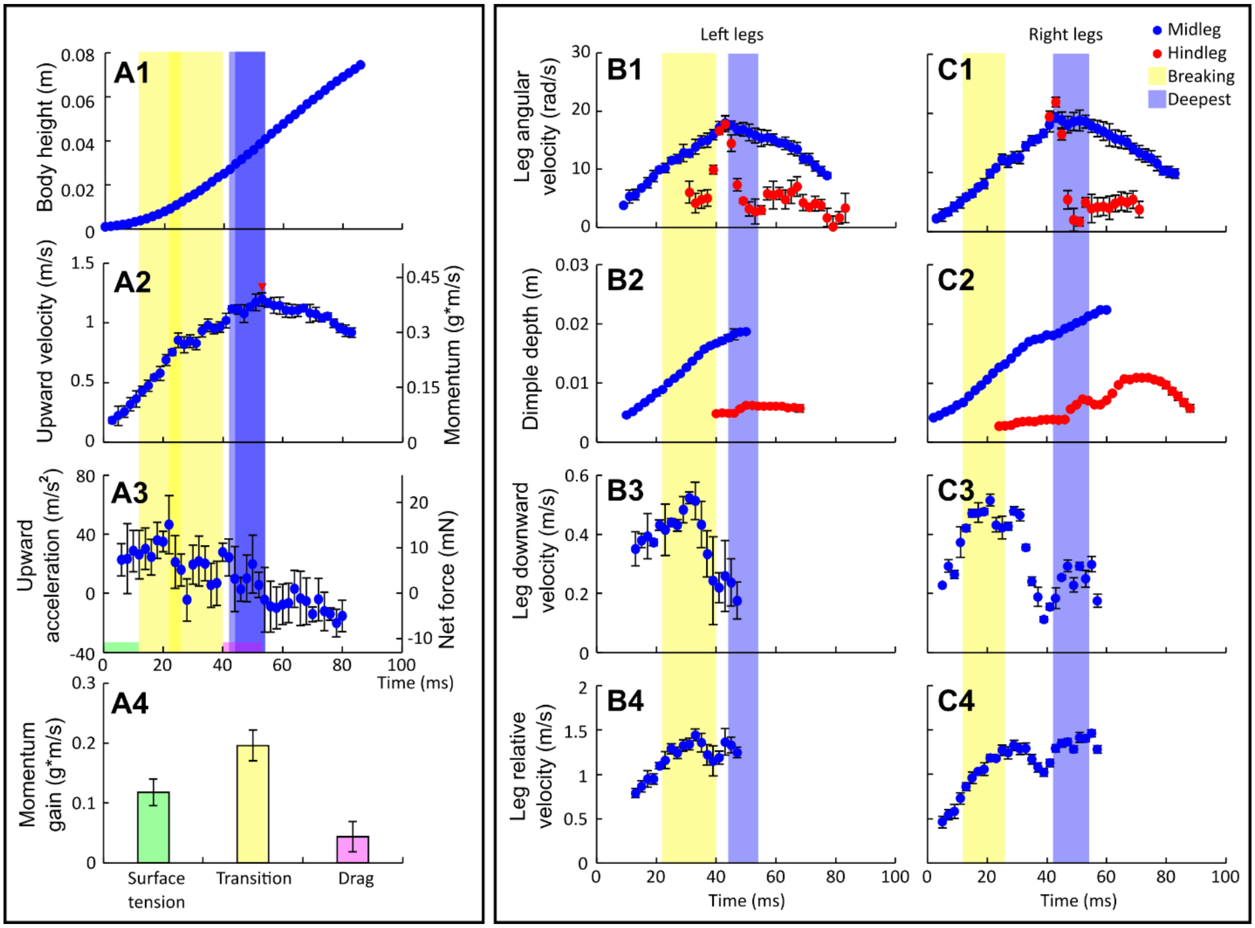

Fig. S6. Kinematics and dynamics of the jumping on water by the giant water strider, *Gigantometra gigas*. Results from analysis of a jump "EVT41". (*A*) variables obtained from the body movement: changes of body height above the water surface (*A*1), body velocity (*A*2) and body acceleration (*A*3) during the jump. Right side axes in *A*2 and *A*3 indicated the changes of body momentum (*A*2) and net force (*A*3) during the jump calculated from the body movement and body mass. (*A*4) shows the comparison between the values of momentum gained during the three phases of jump: the surface tension phase (green), the transition phase (yellow) and the drag phase (purple). (*B*) and (*C*) contain variables concerning movements of the left (*B*) and right (*C*) midlegs (blue circles) and hindlegs (red circles in *B*1, *B*2, *C*1, *C*2): angular downward velocity (*B*1, *C*1), depth (*B*2, *C*2), downward velocity (*B*3, *C*3) and downward velocity relative to the body position (*B*4, *C*4). Yellow background indicates the transition phase when surface is breaking. Blue background indicates the bubble detaching phase duration of each leg. Red triangle in *A*2 indicates the moment of maximal body velocity.

Fig. 3 of the main text, and Fig. S5, S6 present the details extracted from the three best movies. In total we observed 65 jumps (51 in males and 14 in females) by 17 individuals (12 males and 5 females). We also recorded 43 jumps by 5 individuals of *Ptilomera tigrina*. In all observed jumps the water striders broke the water surface and the jump was produced by a mixture of two types of forces: surface tension followed by drag. In all 65 jumps the legs moving in the water were surrounded by the layer of air captured within the long hairs of tibia and tarsi. Also, in all jumps the midlegs moving upward eventually left some of the air in the form of bubbles. In all jumps, we observed the three main phases: surface tension, transition, and drag phase. See table S7 and Fig. S4 for timing of each phase based on timing recorded in 15 leg movement events from 12 videos.

Supplementary Materials PART 6: Assumptions and methods of digitizing

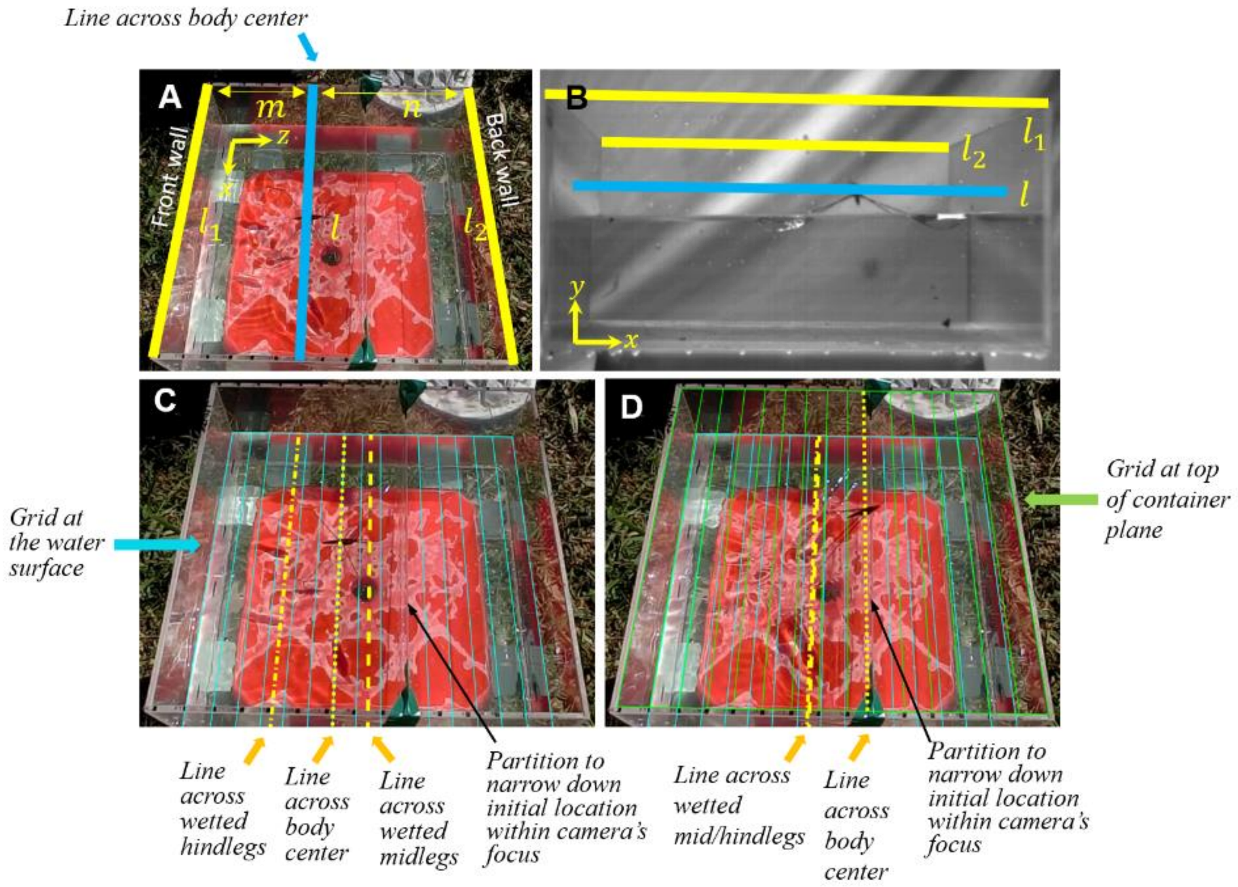

Fig. S7. The method of re-calculation from pixel-based to absolute (cm/mm) coordinates. The jumping of the water strider was recorded with a high-speed camera through the front wall (left side wall in a) of the 30 cm by 30 cm transparent water tank, i.e., the high-speed camera was facing the front wall marked with l_1, and its view is shown in *B*. The views in *A*, *C*, *D* are from a standard camcorder that recorded the jump from above. The thin blue lines at the level of water surface are 2cm apart. The real size of a pixel at any specific distance to the front wall (example of such a point is the water strider's body center through which a thick blue line crosses in a and b) was deduced from the following formula: $30cm/l = 30cm/(\frac{nl_1+ml_2}{m+n})$, where m and n are horizontal relative distances along the *z*-axis from the line across the digitized point (e.g., thick blue line across body center in (*A*) to the front and back wall respectively, and l_1 and l_2 are lengths in pixels of the front and back wall in the view of the slow motion camera. This principle was directly applied to convert pixels to centimeters for the body center digitized (from the high-speed camera view) at the start of a jump (*C*) and at the takeoff (*D*). The body center z-axis at the takeoff was measured by using a grid in the plane corresponding to the upper part of the container (green lines), because the y axis coordinates of the body center at the takeoff were approaching this upper plane and were distant from the plane of the water surface (*D*). Hence for these two points of time, the m and n values were directly measured from an image from the standard camera view from above at two different planes: the water level plane for the start of jump (blue grid at the water surface) and the top-of-container plane for the takeoff moment (when legs still left on water surface; blue grid in *C*, *D*). The conversion from pixels to centimeters for frames located between these two points of time used m and n values calculated assuming a linear change of m (and n) during the jump duration between the initial (*C*; start of jump) and final (*D*; moment of takeoff) values of m and n. The same procedure was applied to the legs. For the two points digitized on legs (point of contact with water and the deepest point of tibia/tarsus for midlegs, as well as femur/tibia joint and the deepest point for hindlegs), we used an approximate distance to the front wall, m (and the corresponding distance to the back wall, n). It was approximately assumed to be the distance between the line going through the midrange of the wetted midleg/hindleg (marked as broken yellow lines in *C*, *D*).

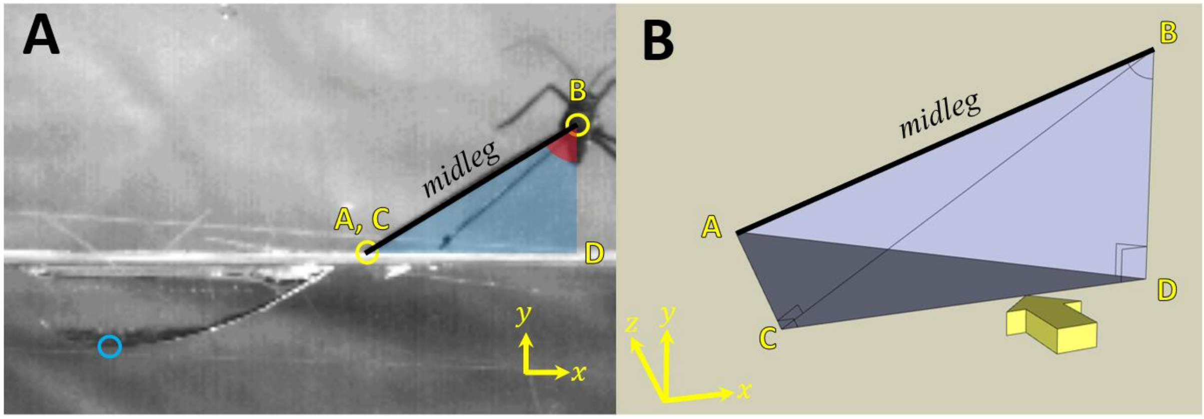

Fig. S8. **Calculations of midleg-to-vertical angles for each frame**. Points on the water surface (A, C), the deepest point (blue circle) of the middle leg, and the body center (B) were tracked in every frame using MaxTRAQ program. The vertical angle of a midleg (ABD) was defined as angle between section AB (along the leg) and vertical line (section BD). However, the camera (3D arrow along the lens's axis indicates the direction to where the camera was pointing) provided direct measure of distances and angles within the plane marked by the triangle CBD (i.e., plane perpendicular to the lens axis). Using this information together with the inferred distance AB (from insect empirical measurements of leg length and proportion of leg above water measured from the video) we estimated (by trigonometry) the angle ABD in each frame in movies with insects facing the camera while jumping. The formulas are explained below:

The angle between the midleg (section AB) and the vertical line (red shaded angle in Fig. S8*A*; angle ABD in b) was calculated in each frame using the trigonometric functions and coordinates of the body center and the water surface contact point of the middle leg. The middle leg angle is expressed as below.

$$\angle \text{Middle leg} = \angle \text{ABD} = \tan^{-1}\frac{\overline{AD}}{\overline{BD}} = \tan^{-1}\frac{(\overline{AC}^2 + \overline{CD}^2)^{1/2}}{\overline{BD}}$$

$$= \tan^{-1}\frac{\sqrt{(Body_z - Surface_z)^2 + (Body_x - Surface_x)^2}}{Body_y - Surface_y}$$

$$= \tan^{-1}\frac{\sqrt{(B_z - A_z)^2 + (B_x - A_x)^2}}{B_y - A_y}$$

The angle between the hind leg's femur and the vertical line was calculated in each frame by the three-dimensional approximation using the second law of the cosines and using the known length of femur (Fig S9). The angle was calculated as below.

$$\angle \text{Hind leg} = \angle \text{ABD} = \cos^{-1}\left(\frac{\overline{AB}^2 + \overline{BD}^2 - \overline{AD}^2}{2 \cdot \overline{AB} \cdot \overline{BD}}\right)$$

$$= \cos^{-1}\left(\frac{\overline{AB}^2 + \overline{BD}^2 - (\overline{AC}^2 + \overline{CD}^2)}{2 \cdot \overline{AB} \cdot \overline{BD}}\right)$$

$$= \cos^{-1}\left(\frac{\overline{AB}^2 + \overline{BD}^2 - [(\overline{AB}^2 - \overline{BC}^2) + \overline{CD}^2]}{2 \cdot \overline{AB} \cdot \overline{BD}}\right)$$

$$= \cos^{-1}\left(\frac{\overline{BD}^2 + \overline{BC}^2 - \overline{CD}^2}{2 \cdot \overline{AB} \cdot \overline{BD}}\right)$$

$$= \cos^{-1}\left(\frac{(B_y - D_y)^2 + \left[\left(B_y - C_y\right)^2 + (B_x - C_x)^2\right] - (D_x - C_x)^2}{2 \cdot \overline{AB} \cdot (B_y - D_y)}\right)$$

$$= \cos^{-1}\left(\frac{(B_y - C_y)^2 + \left[\left(B_y - C_y\right)^2 + (B_x - C_x)^2\right] - (B_x - C_x)^2}{2 \cdot \overline{AB} \cdot (B_y - C_y)}\right)$$

$$= \cos^{-1}\left(\frac{B_y - C_y}{\overline{AB}}\right)$$

The angle was calculated since the length of the femur ($\overline{AB}$) was already known from empirical measurements.

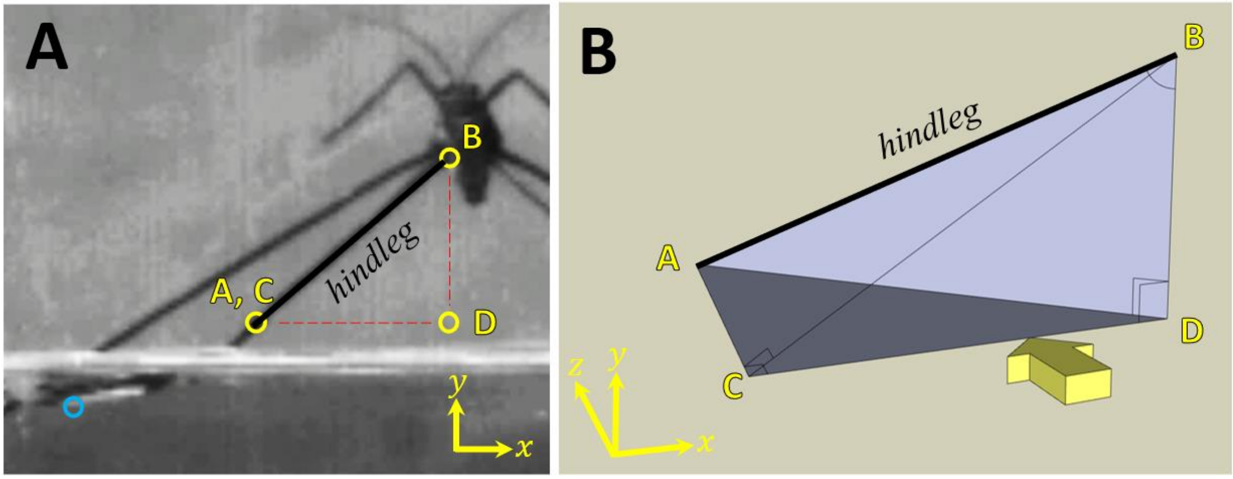

Fig. S9. **Calculations of hindleg-to-vertical angles for each frame**. Hind femur angle was calculated for each frame from the empirically measured femur length and information extracted from the video. As we knew the real femur length of the hind leg ($\overline{AB}$), the angle $\angle ABD = \cos^{-1}(\frac{B_y - C_y}{\overline{AB}})$. Femur tip (yellow) and the deepest point (blue) of the hind leg were also digitized.

Supplementary Materials PART 7: Extraction of information from empirical measurements for the model's assumptions about the functional/effective radius of the wetted leg.

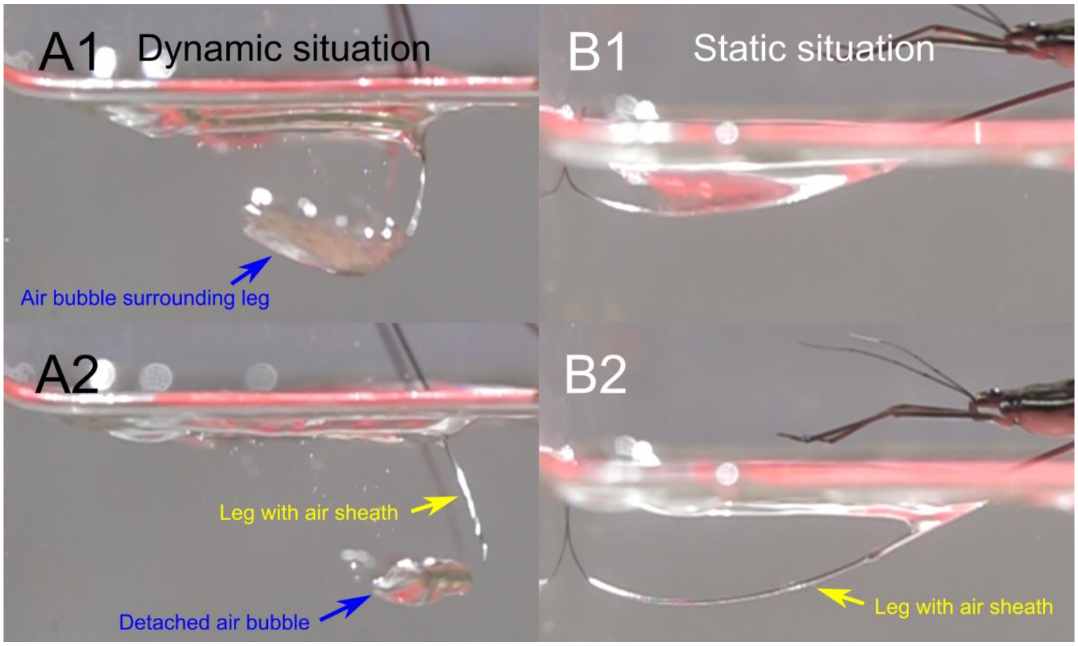

Fig. S10. Air sheath and air bubble exist in both dynamic and static situations. In the dynamic situation (*A*), the wetted leg captures both air sheath and air bubble. The high-speed video frames show that the leg slips out from the air bubble but still keeps the air sheath (*A*2). In the static situation, when the insect accidentally breaks the water surface, the leg only captures and keeps the air sheath (*B*2).

The model dramatically simplifies some of the aspects of reality. One of the simplifications is an assumption about midleg shape that is used to estimate the drag force in the drag phase of a jump. The model assumes that the two midlegs are cylinders (or rods)

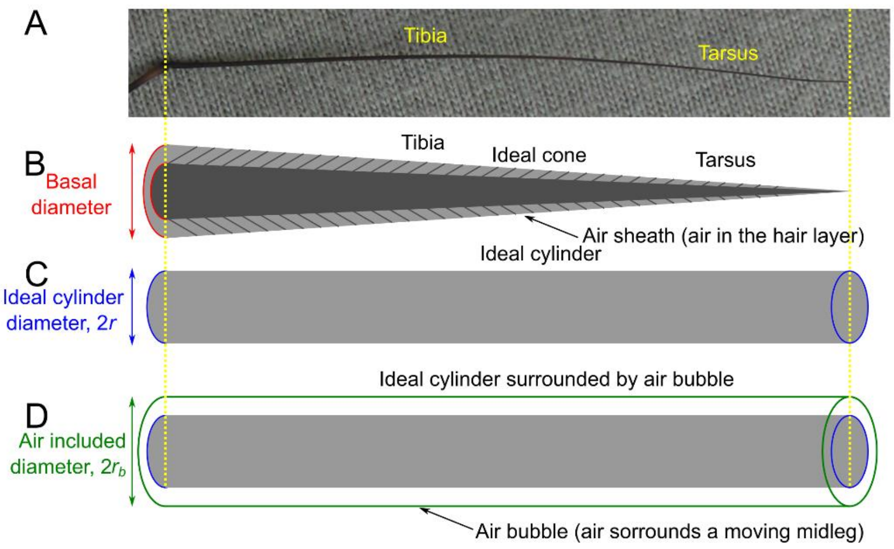

Fig. S11. Tibia and tarsus of the real water strider leg (*A*) considered for calculations. At the first approximation, tibia and tarsus were assumed to form an ideal cone (*B*) of the length corresponding to the total length of tibia and tarsus, and the diameter (including hairs) ranging from the thickness of the proximal tibia at the femur/tibia joint to zero (at the tip). At the next step of approximation, we assumed the leg is a cylinder/rod with the diameter that results in the cylinder's volume in (*C*) equal to the volume of the cone in (*B*). Air-bubble-including radius was calculated by assuming that the volume of the air bubble covers the ideal cylinder evenly (*D*): i.e., knowing the physical length and basal diameter of a wetted leg (*A*, *B*), we estimated the thickness of the leg assuming that air surrounds the leg in a uniform symmetrical manner (*D*).

of a diameter based on the empirical measurements of legs of water striders and on the empirical estimates of the air volume captured around the leg during a jump. The functional (effectively working for drag force) leg diameter during the initial moments of the drag phase, when air bubble surrounds the leg, is calculated assuming that the air bubble surrounds the cylinder as a uniform layer (Fig. S11*D*) of a thickness calculated from the empirically derived measurements of the volume of air caught around the leg (Table S8). We measured the basal diameter of the legs, including their hair layer, as shown in Fig. S11*B*, based on empirical data. This is because the air sheath captured by the hairs always remains attached to the legs in both dynamic and static situations, as depicted in Fig. S10. As a result, we were able to estimate the volume of the air bubble (excluding the air sheath) by measuring the detached air bubbles (Fig. S10*A*).

Table S8. Calculated ideal radius of a cylinder imitating the midleg (Fig. 11*C*), and the ideal radius of a cylinder imitating the midleg surrounded by air bubble (Fig. 11*D*), as well as the ratio between these two radii (radius with bubble to radius without bubble). The observed minimum value is colored blue, and the maximum value is colored red in the table. Data derived from slow motion movies of *G. gigas*.

Videos	Ideal radius (mm)	Observed bubble volume for a leg (mm^3)	Ideal bubble included radius (mm)	Radius ratio
EVT16	0.131	43.4	0.520	3.96
EVT05 (2)	0.113	9.9	0.285	2.54
EVT41	0.137	36.3	0.482	3.52
EVT75	0.149	31.3	0.461	3.10
EVT45	0.137	26.9	0.421	3.07
EVT47	0.137	78.2	0.692	5.05
EVT63	0.117	7.8	0.261	2.24
EVT65	0.117	16.4	0.358	3.06
EVT67	0.117	23.1	0.419	3.58
EVT00	0.113	23.1	0.416	3.70
EVT03 (2)	0.113	18.2	0.373	3.31
EVT12	0.131	56.6	0.589	4.49
EVT14	0.131	61.1	0.611	4.65
EVT28	0.117	11.3	0.323	2.76
Mean (S.D.)	0.126 (0.012)	31.7 (21.2)	0.444 (0.126)	**3.50** (0.81)

The model simulated several situations of different radius of leg cylinder (or rod) to imitate the leg with the air bubble around it assuming the “radius ratio” (Table S8) of 2.24, **3.50**, and 5.05 to simulate the situation of the minimum, average, and maximal air volume of the air bubble trapped around the moving leg. We also imitated that ratio of 1 to simulate the leg without any additional air bubble trapped around the moving leg with air sheath in the hair layer.

Supplementary Materials PART 8: Observations of midleg dimple breaking and the role of the breaking process in the simulation model.

The model is a simplified representation that does not directly simulate a leg breaking the surface at a different point along the

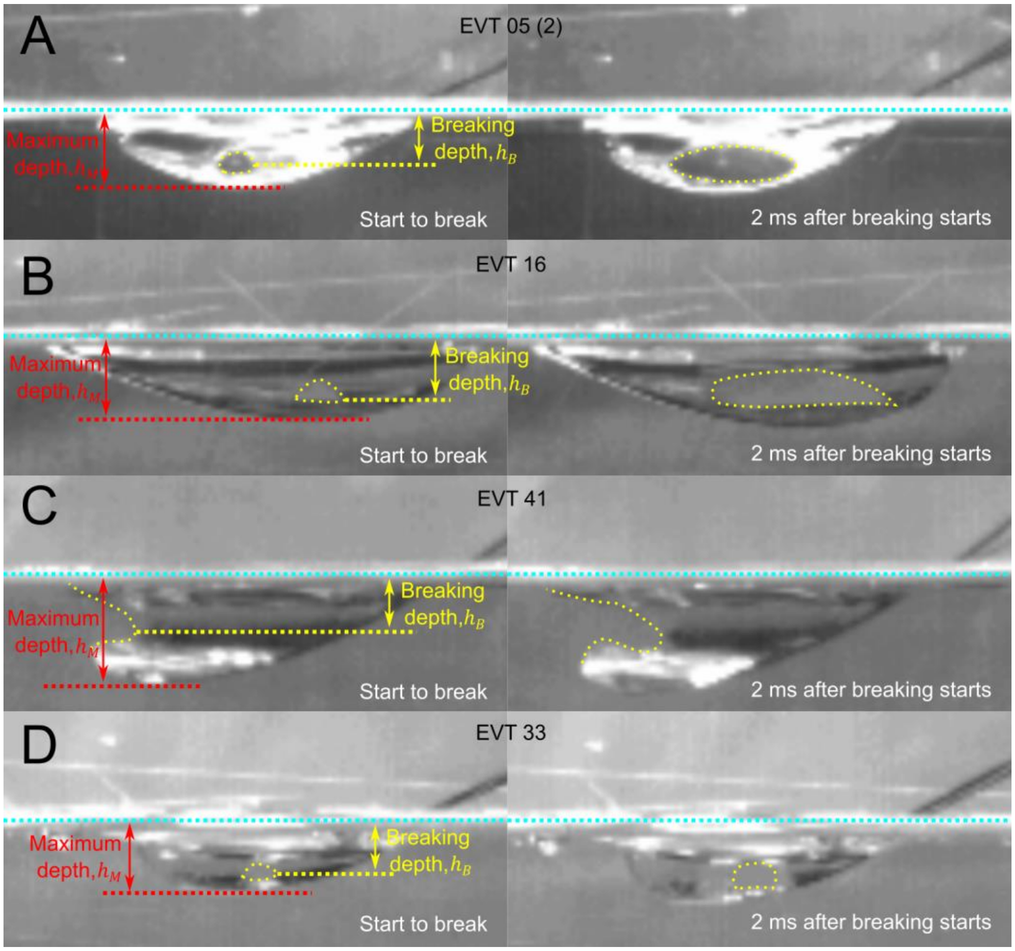

Fig. S12. Examples of dimple breaking by the midleg of the giant water strider during jumps. The dimple starts to break when the leg reaches the maximum depth of the dimple ($\boldsymbol{h_M}$, red arrows). However, the breaking of the dimple does not occur at the deepest point (i.e., not at the maximum depth, $\boldsymbol{h_M}$), but rather at a certain depth (yellow arrows) that is shallower than the maximum depth: the depth of breaking initiation, $\boldsymbol{h_B}$. The breaking initiation point varies and the breaking can start either at the middle of the dimple (*A*, *B*, *D*) or at the end of the dimple in the longitudinal direction (*C*). The breaking of the dimple expands along the leg in both longitudinal directions (*A*, *B*, *D*) or in a single direction (*C*), which can potentially affect the duration of the breaking process. The right panels of the figure depict this phenomenon 2 ms after the start of breaking.

wetted leg than the point of maximum dimple depth (Fig. S12). In the previous model by Yang et al. (1), a leg was modeled as a horizontal cylinder (or rod) based on a theoretical model of a cylinder by Vella (3), and it was assumed that water surface breaks at a critical dimple depth in a single moment. However, our model attempts to imitate an additional transition phase during which a combination of surface tension and drag contribute to the jump. We assume that the transition phase begins at the critical time, t_c, when the theoretically modeled dimple depth of the two horizontal cylinders (representing the two symmetrically moving midlegs in the model) reaches the critical depth, h_c. This critical depth is calculated in the model from the regression formula of h_c, (defined as the average between the breaking point depth, h_B, and the maximum dimple depth at the breaking moment, h_M) which was measured in videos of water striders of different sizes (Fig. S12), on the wetted midleg length (Fig. S13*A*). In addition, the model uses an empirical relationship shown in Fig. S13*B* to calculate an index of maximum dimple depth, $i = h_M/l_c$, for water striders of different sizes. This index modifies Yang's (1) formula, where the original $2l_c$ part (denominator) of the surface tension formula is replaced by "il_c" (Formula 6). This modification allows the deeper dimple depth for theoretical simulation of surface tension than the original Vella's model of a rigid cylinder (3) used by Yang et al. (1).

Once the transition phase starts at t_c, it lasts for a duration of D_b, which is calculated in the model from the empirically derived regression in Fig. S14. During this time, the surface tension from the two midlegs is calculated based on the assumed dimple depth of h_c and the wetted length of the horizontal rod/cylinder (representing the midleg) that gradually decreases from 100% to 0% of the midleg tibia+tarsus length over the period D_b. Simultaneously, the drag force gradually increases over the same period, as the length of the horizontal cylinder (rod) moving downward in the water increases from 0% to 100% of the midleg tibia+tarsus length. The horizontal rod's downward velocity, which also contributes to the drag, is calculated in the model, considering the angular velocity of midleg rotation, ω, the height of the insect body above the surface, and the body's upward velocity, in accordance with Yang et al. (1).

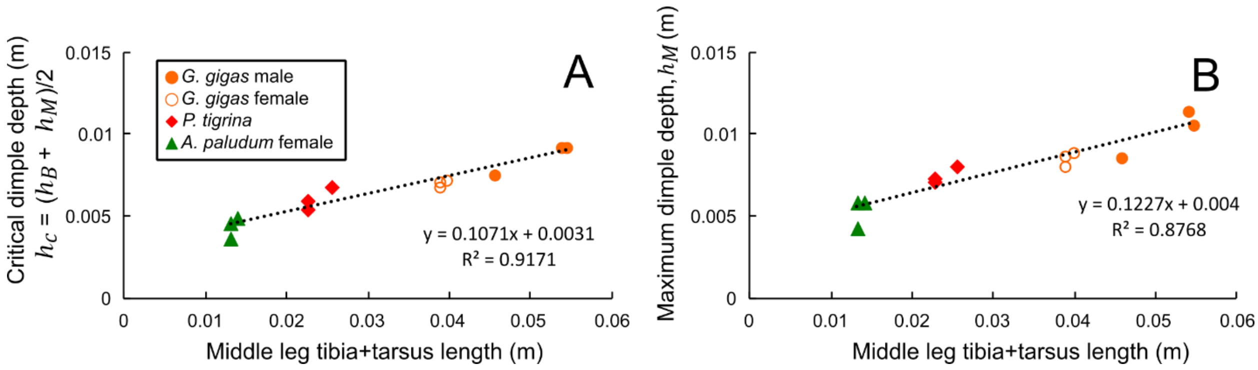

Fig. S13. Relationship between midleg tibia+tarsus length (constant wetted midleg length) and dimple depth measures derived from the videos of jumping water striders of different sizes.
The breaking point depth, $\boldsymbol{h_B}$, and the maximum dimple depth at breaking moment, $\boldsymbol{h_M}$, defined in Fig. S12, were found to be linearly related to the wetted length of the middle leg among the studied species who use surface breaking jumps. The average of $\boldsymbol{h_B}$ and $\boldsymbol{h_M}$ was used to determine $\boldsymbol{h_c}$ for water striders with various sizes using linear regression of depth on middle leg constant wetted length (*A*). The $\boldsymbol{h_M}$ was used to determine index of dimple depth, $\boldsymbol{i}$, in the model for water striders with various sizes using linear regression of depth on middle leg constant wetted length (*B*).

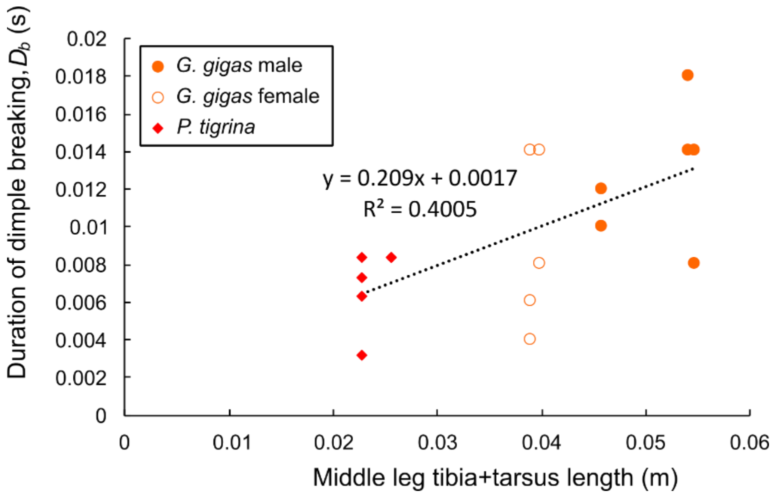

Fig. S14. Relationship between midleg tibia + tarsus length (constant wetted midleg length) and the duration of dimple breaking phase of a midleg, $\boldsymbol{D_b}$, which is also the duration of the transition phase if both midlegs act in an entirely symmetrical manner (assumed in the model). Several factors can affect the duration of dimple breaking, including the wetted length, leg downward velocity, and breaking point (whether at the center or end of the dimple in the leg's longitudinal direction). For simplicity, the regression of the empirically observed duration of breaking, $\boldsymbol{D_b}$, on the midleg tibia + tarsus length (constant wetted midleg length) was used in the model to predict the duration of dimple breaking, $\boldsymbol{D_b}$, for water striders of different sizes (midleg tibia + tarsus length). However, we were unable to include data from *A. paludum* in the regression because their complete breaking of the dimple was not observed under natural conditions.

Supplementary Materials PART 9: Observations of hindlegs in the jumps of *Gigantometra gigas*.

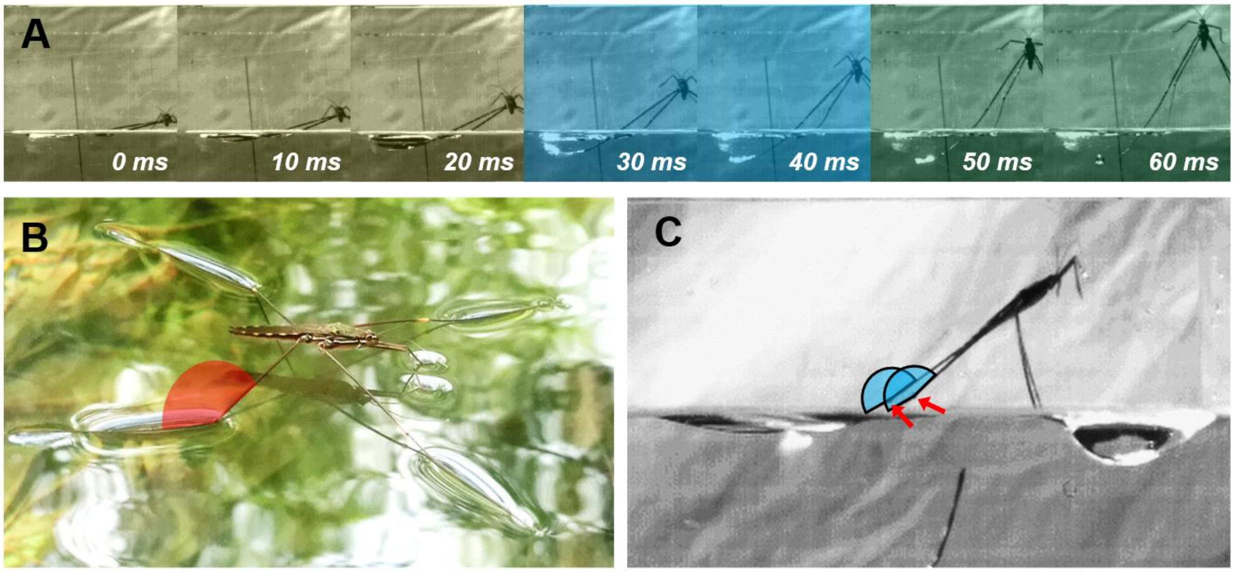

Fig. S15. Hindlegs in the jump of the giant water strider. Changes of body pitch during the jump analyzed in Fig. 3 are the most pronounced in the final phase (40-60 ms in a), when a sudden short-lasting increase in the downward angular velocity of hindlegs occurs (in Fig. 3, S5, S6). Hindleg's dimples depth remain relatively stable at the initial phases (0-20 ms of surface tension phase in a) and subsequently during the transition and drag phases (30-40 ms in *A*), and they are generally similar to the dimples in resting position (b). During the jump, the angle between femur and tibia at the femur/tibia joint appears to remain roughly similar for extensive portion of the jump. The resulting dimples during jump may deepen slightly in the final stages of jump when the body pitch changes, and when the femur/tibia angle (marked red) becomes wider and approaches 180° in the final stages of the hindlegs' leaving the water surface. However, this typically this does not lead to breaking the water surface (*A*, *C*) because the hindlegs bend quite extensively (*C*) and because the water strider's legs at this stage are already moving upwards and do not push the surface (green shaded frames in a). Red arrows in (*C*) mark the femur-tibia joints for each hindlegs.

Supplementary Materials PART 10: Hindleg's constant depth, h_{hm}.

To calculate for each movie and species the theoretically assumed constant depth of a hindleg (h_{hm}; see page 36 in Supplementary Materials PART 19), we used empirical maximum depth from high-speed videos. The constant depth used in the model (h_{hm}) was calculated from the empirical maximum depth, h_{hE}, and wetted leg length, l_h, assuming the wetted length as a half of an arc. The average depth of the arc was used as the constant depth.

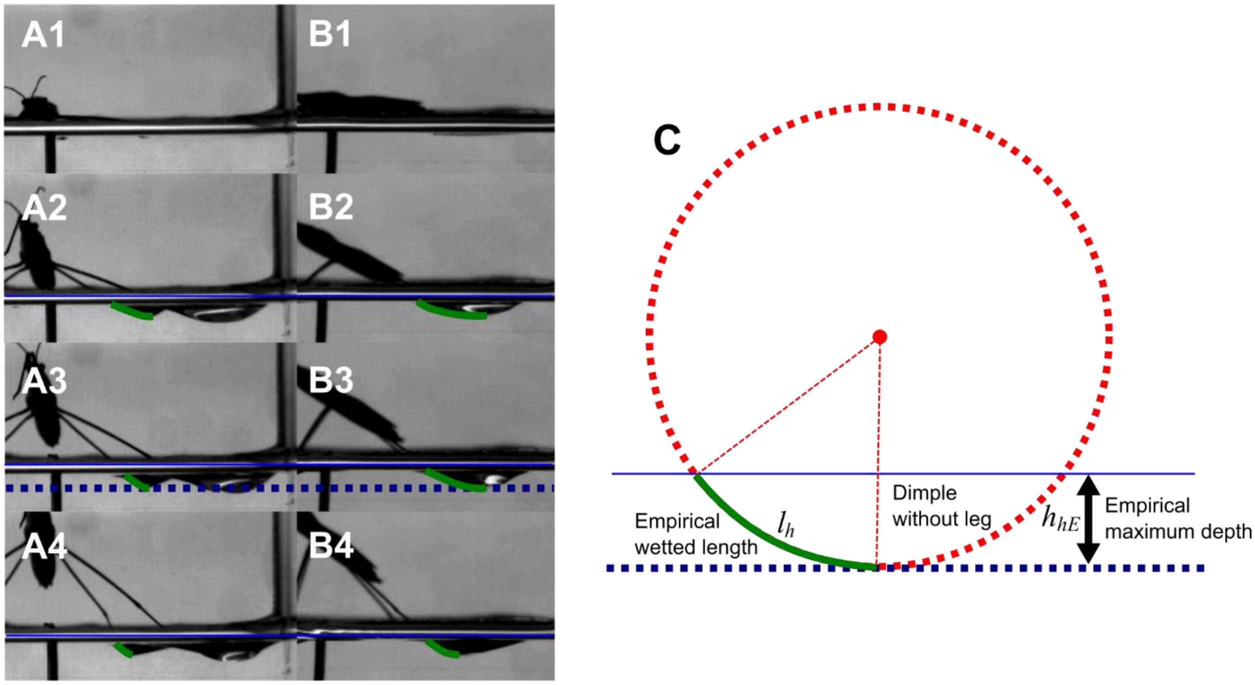

Fig. S16. The constant depth of hind leg. The constant depth, $\boldsymbol{h_{hm}}$, was calculated as an average depth of a half of an arc that has same maximum depth, $\boldsymbol{h_{hE}}$ and wetted length, $\boldsymbol{l_h}$, of empirical measurements of species.

Supplementary Materials PART 11: Maximum jumping height calculation.

If an object takes off vertically at the moment of takeoff, t_f, with the takeoff velocity, v_f, from initial height, H_0, the object will be at the maximum height, H_m, when the total kinetic energy transferred to potential energy. Hence, the maximum jumping height, H_m, can be calculated as follows:

$$E_{potential} = mgh = mg(H_m - H_0) = E_{kinetic} = \frac{1}{2}mv_f^2$$

$$H_m = H_0 + \frac{v_f^2}{2g}$$

Supplementary Materials PART 12: Additional empirical results for *G. gigas* females and *P. tigrina*

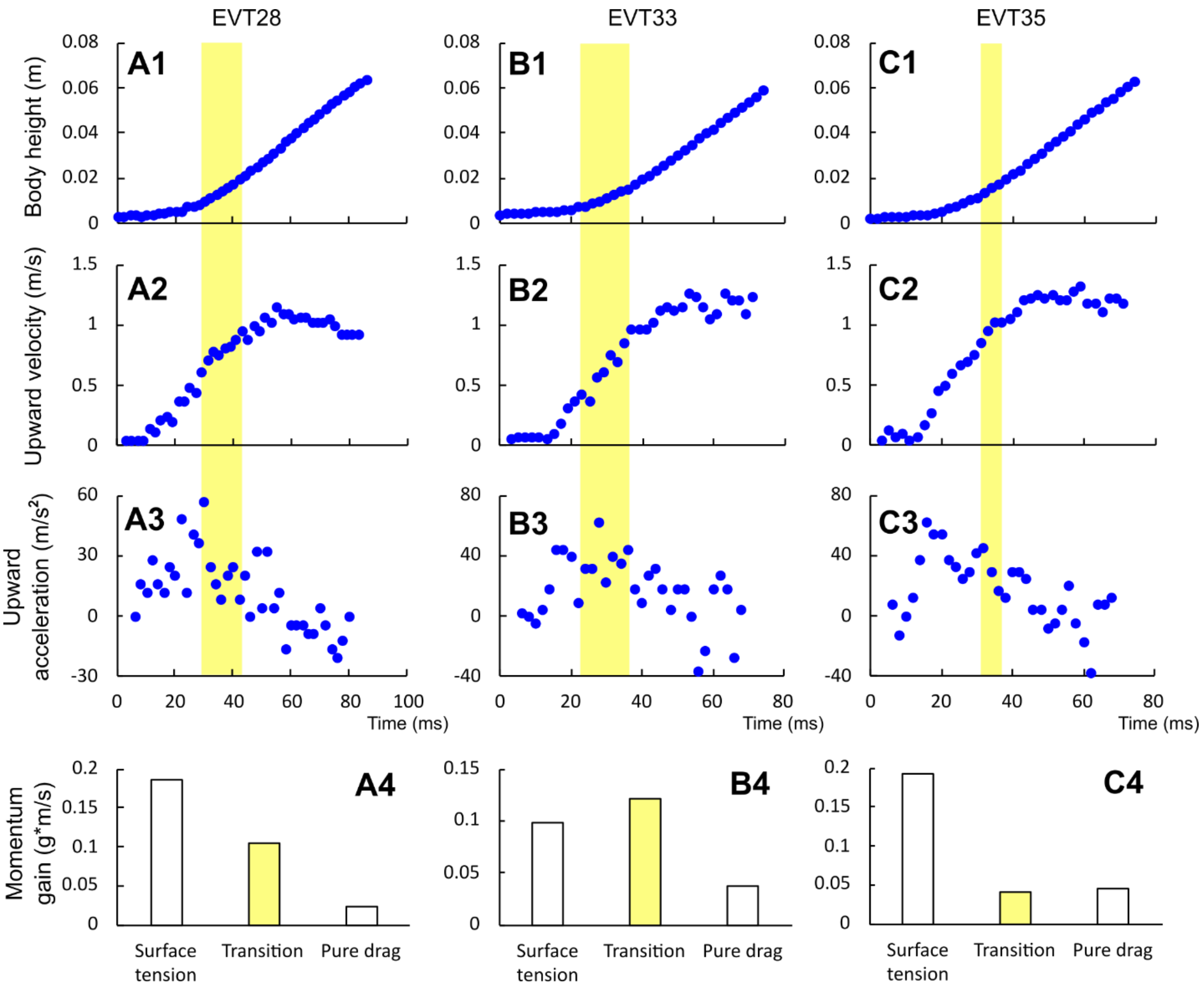

Fig. S17. Kinematics of the jumping on water by the giant water strider (*Gigantometra gigas*) females. Extra results from analyses of jumps “EVT28”, “EVT33”, “EVT35”. *A*1, *B*1, *C*1 show body height; *A*2, *B*2, *C*2 show body velocity; *A*3, *B*3, *C*3 show body acceleration during the jump. *A*4, *B*4, *C*4 show the comparison between the values of momentum gained during the three phases of jump: the surface tension phase, the transition phase (yellow), and the drag phase. The vertical yellow band across the panels indicates the transition phase when surface is in the process of breaking.

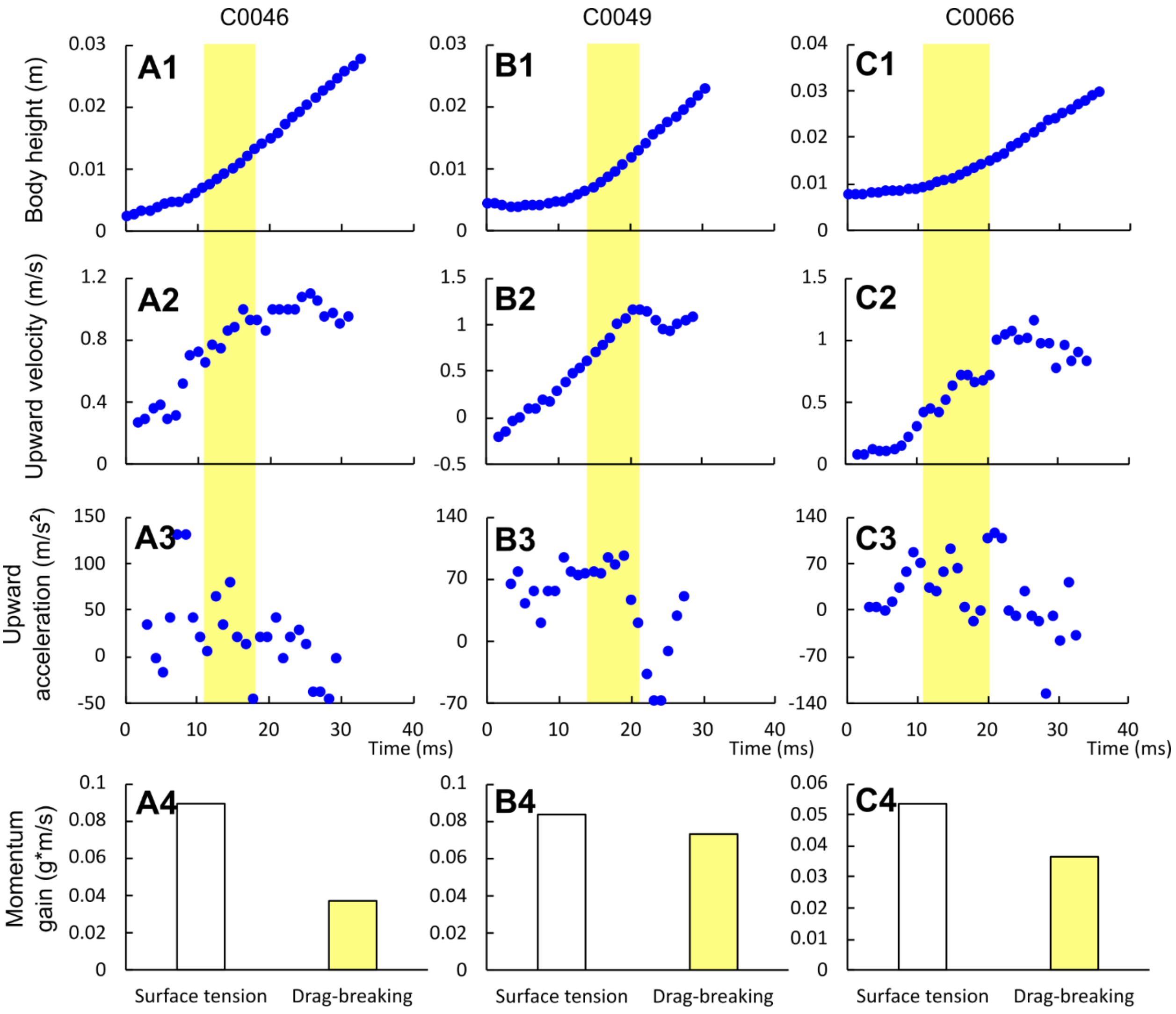

Fig. S18. Kinematics of the jumping on water by *Ptilomera tigrina*. Results of analysis of jumps in movies "C0046", "C0049", "C0066". *A*1, *B*1, *C*1 show body height; *A*2, *B*2, *C*2 show body velocity; *A*3, *B*3, *C*3 show body acceleration during the jump. *A*4, *B*4, *C*4 show the comparison between the values of momentum gained during the two phases of jump: the surface tension phase, and the drag-breaking phase (corresponding to the transitional phase in *G. gigas)*. Pure drag phase is not observed because *P. tigrina* legs go up before full breaking of the middle leg dimple. Yellow vertical bands across the panels indicates the "drag-breaking" phase (i.e., the transition phase) when surface is in the process of breaking under the midlegs (see also Supplementary Movie 3).

Supplementary Materials PART 13: Model validation - comparison with empirical observations

The supplementary results of model validation are similar to those shown in Fig. 3 in the main text. The results illustrate a reasonable match between empirical (Data from Supplementary Materials PART 12) and theoretical trajectories of body center for specific jumps of three females of *G. gigas* (Fig. S19*A*-*C*) and three individuals of *P. tigrina* (Fig. S19*D*-*F*).

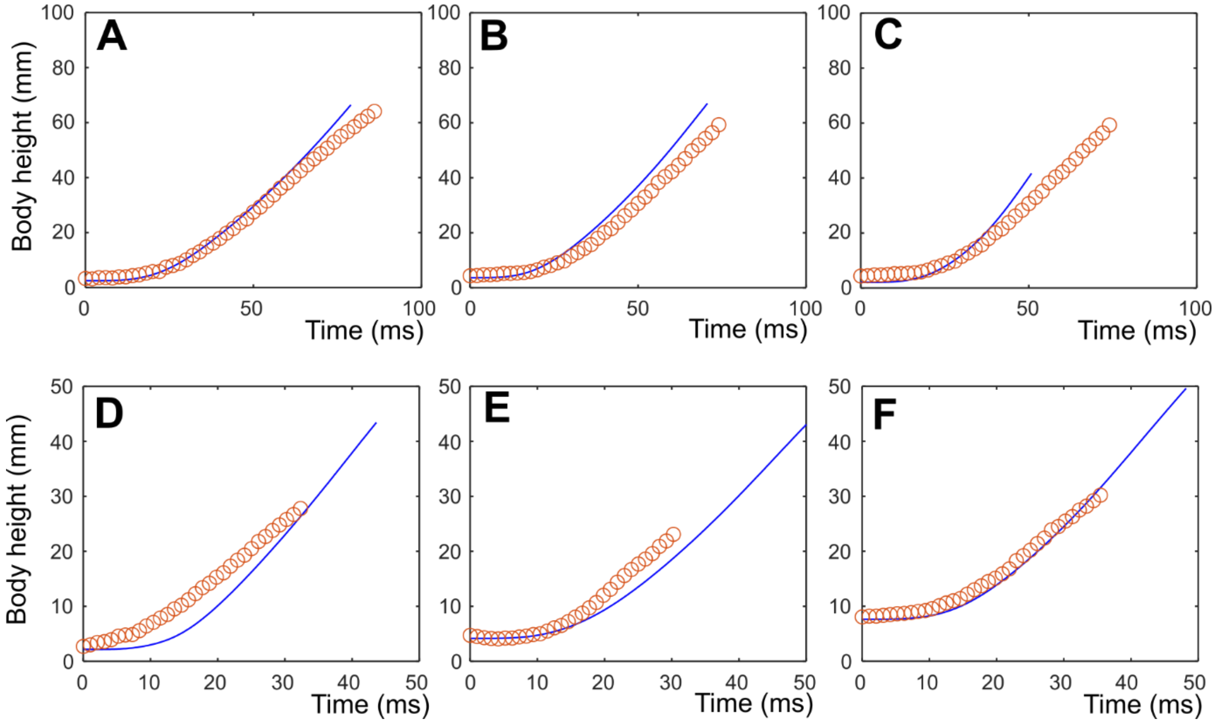

Fig. S19. The model results (simulations of separate jumps based on empirically measured input into the model) compared to the empirically observed trajectories for individual jumps of *G. gigas* females and *P. tigrina*. (*A*, *B*, *C*) - The theoretical model results and empirical results for the jumps of *G. gigas* females in movies EVT28, EVT33, and EVT35. (*D*, *E*, *F*) - The theoretical model results and empirical results for the jump videos of *P. tigrina*: C0046, C0049, and C0066. Horizontal axis represents time (ms); Vertical axis represents height of body center above water surface (mm). Blue line indicates model results and orange circles represent empirically measured values.

Supplementary Materials PART 14: Validation of the use of empirical constant angular velocity of the middle leg rotation, ω_e.

One of the model's simplifying assumptions is that the angular downward midleg movement occurs at a constant angular velocity that can be determined from empirical data on leg and body coordinates from the videos under certain assumptions described in Yang et al. (1), where it was shown to be acceptably close to the empirical average of angular velocity and resulted in valid conclusions regarding the surface tension jumps in small and medium size water striders. This angular velocity of middle leg rotation, ω_e, is empirically derived from basic observations of jump kinematics under the assumption that the empirically measured linear downward velocity of wetted midleg relative to water surface, v_l, can be approximated by the simple formula: $v_l = \omega(l_l - y_i)sin(2\omega t) - \dot{y}$, while the vertical distance from the tip of the legs to the body center, l_s, can be approximated by another formula: $l_s = \Delta l[1 - \frac{1}{2}\cos(2\omega t) + y_i]$ (see Yang et al. (1) and Supplementary Materials PART 19 for more details). To validate our use of the same procedure for determining the simple value of constant angular velocity of middle leg rotation, ω_e, we compared the empirically observed leg movements in specific jumps where in reality the leg angular velocity of rotation vary during a jump with the theoretical leg movements calculated using the constant value of ω_e extracted from each video separately. We did this for 3 jumps from each size class: *G. gigas* male, *G. gigas* female, and *P. tigrina* (data from Fig. 3, S13, S14, S15). The results suggest an approximate reasonable agreement between observed and theoretically predicted profiles of leg tip distance to body and leg tip relative velocity during jumps performed by relatively synchronized symmetrical movements by the left and right midleg.

In order to determine the theoretical performance for "virtual" water strider jumps that do not occur in nature we calculated the three hypothetical angular midleg velocities, ω_t, that are theoretically expected in the hypothetical situations of the three large classes using surface tension jumps and in the hypothetical situation of *A. paludum* using drag-involving jump. For the former, we first extracted ω_e values from the clips of jumps of *A. paludum,* which resulted in the range of ω_e between 23-41 rad/s (n = 7, Table S9). This corresponds to 56-99% [calculated as (ω_e/ω_c)·100%] of the theoretical critical midleg angular velocity, ω_c, for *A. paludum* (marked as ω_c in Fig. 5) at which the water surface breaks. Then, we decided that the best feasible estimates of the hypothetical surface tension jumps' performance by the three large water strider classes are represented by the performance for the range the midleg angular velocity corresponding to 56-99% of ω_c for each of the three classes of large water striders (i.e., the range of ω_t is from $0.56\omega_c$ to $0.99\omega_c$). Similarly, we calculated theoretical performance in the hypothetical drag-involving jumps by *A. paludum* assuming the range of angular midleg velocities calculated as corresponding to the hypothetical midleg angular velocity (ω_t) range from $1.13\omega_c$ to $1.69\omega_c$ (1.13 is the average of the three lowest ω_e/ω_c ratio, and 1.69 is the average of the three highest ω_e/ω_c ratio for the three classes of large water striders calculated from the empirically based ω_e and from the theoretically calculated ω_c for each of the three classes separately; n = 6 for each class, Table S9).

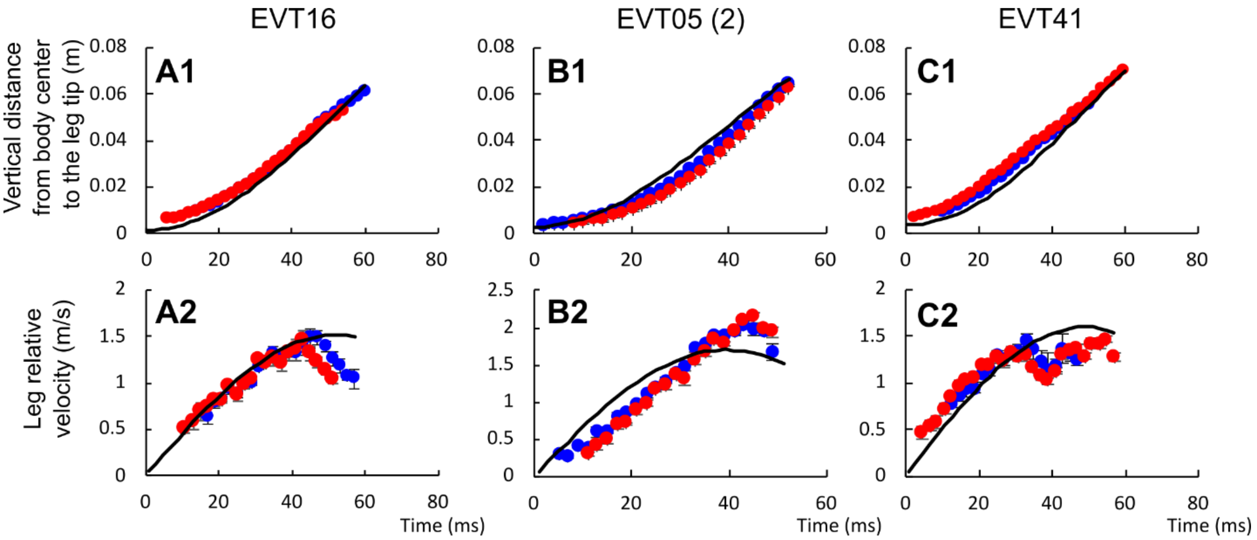

Fig. S20. Three examples of the comparison of theoretical predictions based on constant midleg angular velocity, ω_e, extracted from the video analysis according to (1) with empirically derived variables directly measured from the video of water striders (*G. gigas* male), who naturally use midleg angular velocity that varies during the course of a jump. (*A*1, *B*1, *C*1) – The profile of the vertical distance from body center to the leg tip in jumps by *G. gigas* males; (*A*2, *B*2, *C*2) - The profile of the velocity of the leg relative to the body center in jumps of *G. gigas* males. Data from videos EVT16 (*A*1, *A*2), EVT05 (2) (*B*1, *B*2), and EVT41 (*C*1, *C*2). Red dots indicate left leg and blue dots indicate right leg.

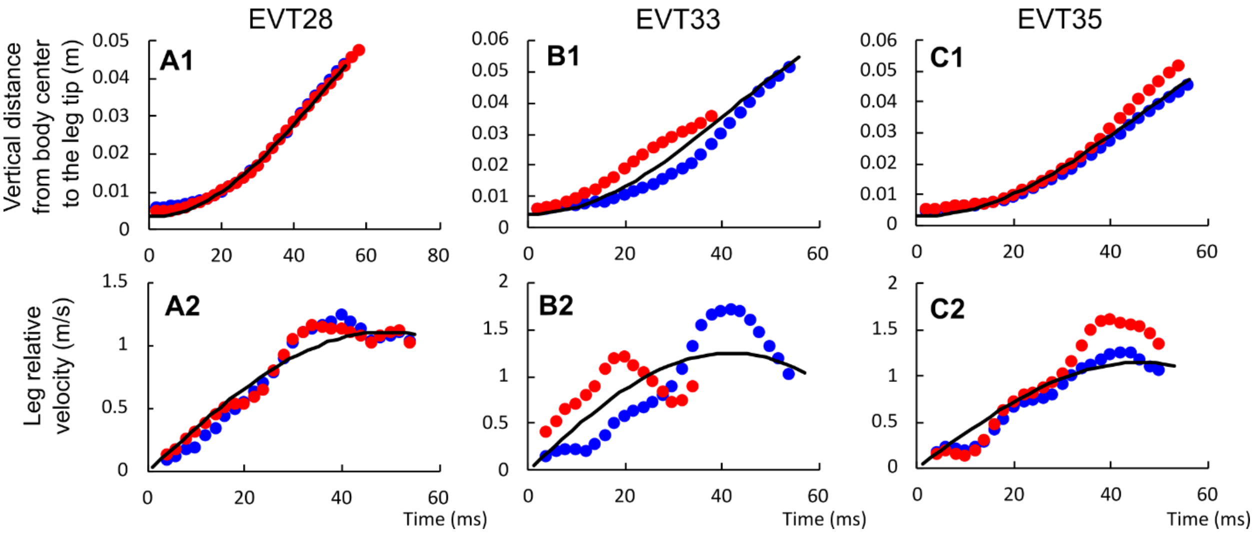

Fig. S21. Three examples of the comparison of theoretical predictions based on constant midleg angular velocity, ω_e, extracted from the video analysis according to (1) with empirically derived variables directly measured from the video of water striders (*G. gigas* female), who naturally use midleg angular velocity that varies during the course of a jump. (*A*1, *B*1, *C*1) – The profile of the vertical distance from body center to the leg tip in jumps by *G. gigas* females; (*A*2, *B*2, *C*2) - The profile of the velocity of the leg relative to the body center in jumps of *G. gigas* females. Data from videos EVT28 (*A*1, *A*2), EVT33 (*B*1, *B*2), and EVT35 (*C*1, *C*2). Red dots indicate left leg and blue dots indicate right leg.

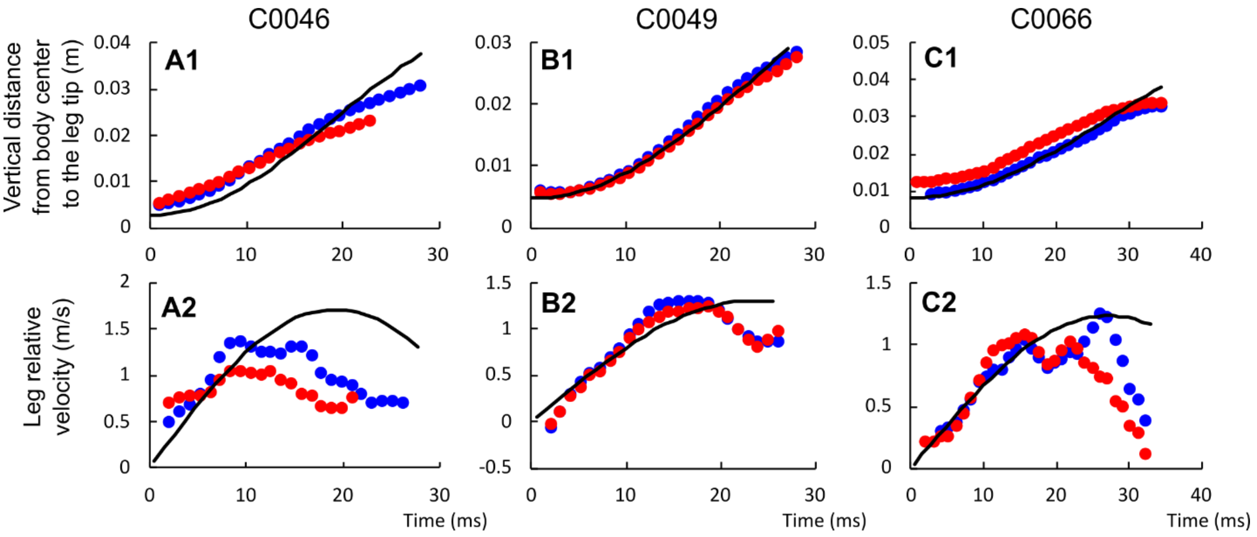

Fig. S22. Three examples of the comparison of theoretical predictions based on constant midleg angular velocity, ω_e, extracted from the video analysis according to (1) with empirically derived variables directly measured from the video of water striders (*P. tigrina*), who naturally use midleg angular velocity that varies during the course of a jump. (*A*1, *B*1, *C*1) – The profile of the vertical distance from body center to the leg tip in jumps by *G. gigas* males; (*A*2, *B*2, *C*2) - The profile of the velocity of the leg relative to the body center in jumps of *G. gigas* males. Data from videos C0046 (*A*1, *A*2), C0049 (*B*1, *B*2), and C0066 (*C*1, *C*2). Red dots indicate left leg and blue dots indicate right leg.

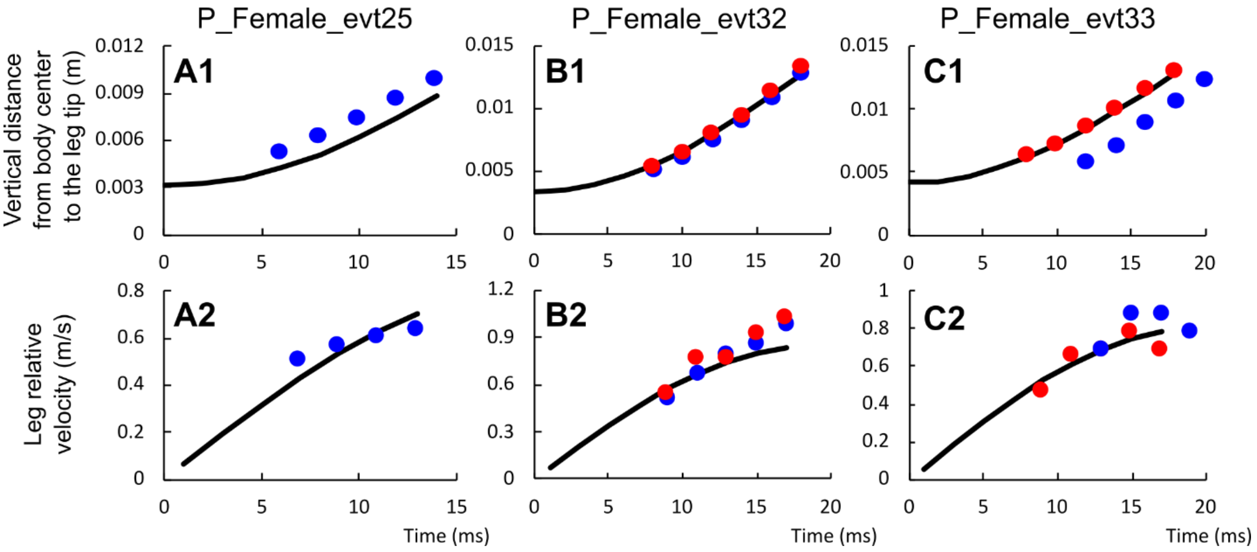

Fig. S23. Three examples of the comparison of theoretical predictions based on constant midleg angular velocity, ω_e, extracted from the video analysis according to (1) with empirically derived variables directly measured from the video of water striders (*A. paludum* female), who naturally use midleg angular velocity that varies during the course of a jump. (*A*1, *B*1, *C*1) – The profile of the vertical distance from body center to the leg tip in jumps by *G. gigas* males; (*A*2, *B*2, *C*2) - The profile of the velocity of the leg relative to the body center in jumps of *G. gigas* males. Data from videos P_Female_evt25 (*A*1, *A*2), P_Female_evt32 (*B*1, *B*2), and P_Female_evt33 (*C*1, *C*2). Red dots indicate left leg and blue dots indicate right leg. We also extracted the values of constant empirical angular velocity of leg rotation, ω_e (1), in these jumps in order to use the values in Fig. 5, 6 as the indicators of the range of values of midleg angular velocity by this species. Only one leg was digitized in P_Female_evt25 since the other was not visible.

Table S9. Calculated empirical angular velocity of leg rotation, $\boldsymbol{\omega_e}$, for 6-7 jumps analyzed in details for each size class.

Species/sex	Individual	Mass (mg)	Video	Empirical angular velocity of leg rotation, $\boldsymbol{\omega_e}$
G. gigas male	19	374.76	EVT05 (2)	20
G. gigas male	20	483.23	EVT16	15
G. gigas male	12	315.64	EVT39	19
G. gigas male	13	325.41	EVT41	16
G. gigas male	3	424.01	EVT70 (2)	15
G. gigas male	4	404.27	EVT75	16
G. gigas female	8	315.64	EVT03	19
G. gigas female	8	315.64	EVT05	27
G. gigas female	21	305.67	EVT28	16
G. gigas female	11	226.81	EVT31	25
G. gigas female	11	226.81	EVT33	19
G. gigas female	11	226.81	EVT35	17
P. tigrina	1	134	C0044	31
P. tigrina	1	134	C0045	29
P. tigrina	1	134	C0046	41
P. tigrina	1	134	C0049	33
P. tigrina	2	106	C0061	27
P. tigrina	3	123	C0066	29
A. paludum female	1113	45.2	P_Female_evt1,2	33
A. paludum female	1113	45.2	P_Female_evt3,4	27
A. paludum female	1114	48.5	P_Female_evt7,8	27
A. paludum female	1114	48.5	P_Female_evt25,26	39
A. paludum female	2111	42.6	P_Female_evt31,32	40
A. paludum female	2111	42.6	P_Female_evt33,34	41
A. paludum female	2113	54.2	P_Female_evt35,36	23

Supplementary Materials PART 15: Additional simulation results for different Young's modulus of insect cuticle.

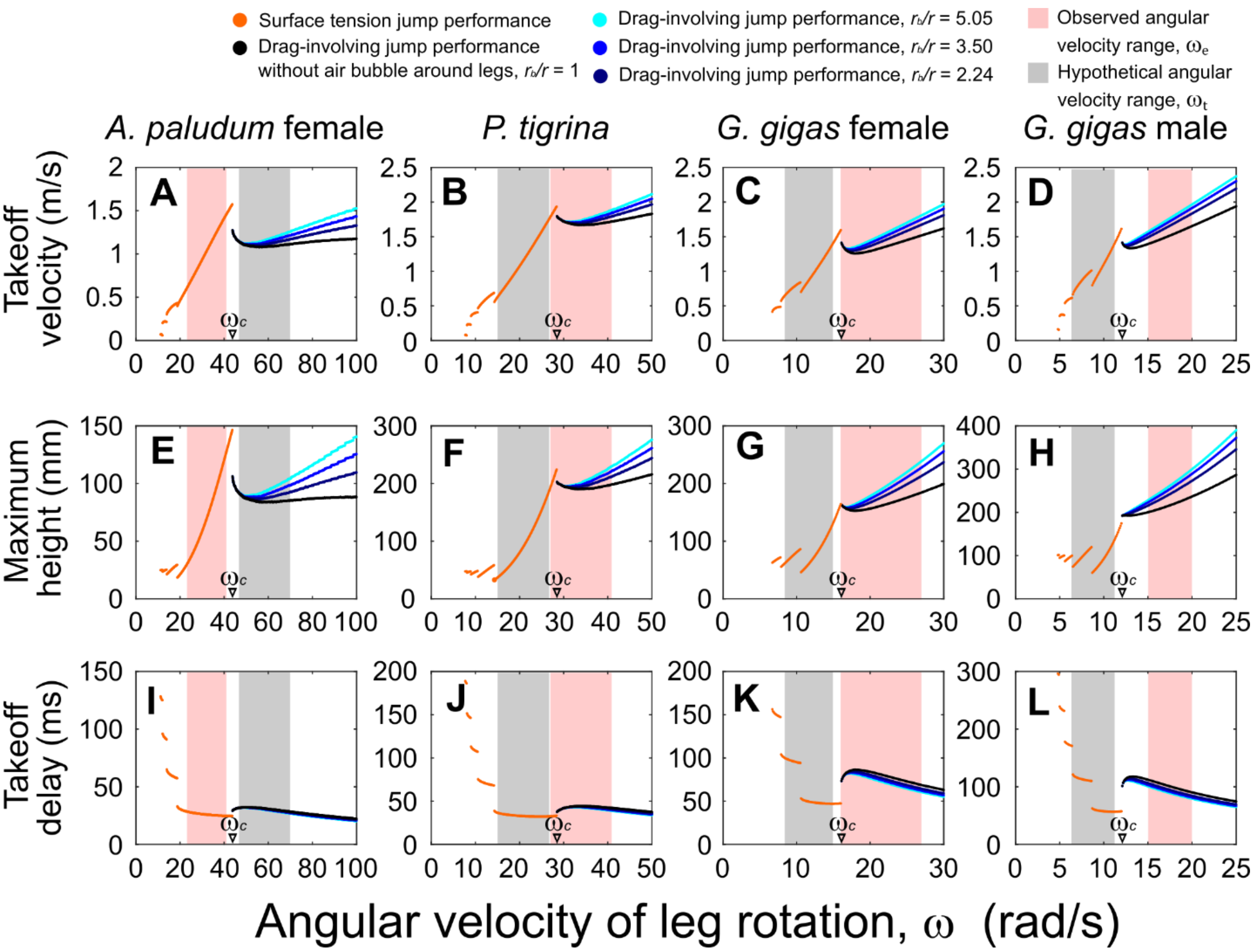

Fig. S24. Theoretically predicted jump performance as a function of midleg angular velocity for four classes of water striders' body size based on *A. paludum* females, *P. tigrina*, *G. gigas* females, and *G. gigas* males when E = 15 GPa. Jump performance measured by three variables calculated by the model: takeoff velocity (*A-D*), maximum jump height (*E-H*), takeoff delay (*I-L*). Average empirical values (mass, leg length for each leg section, leg radius, initial height of the body; average values are shown in Tables S1, S3) for each body size class were used to simulate the jumps for each body size class across a wide range of angular velocity of leg rotation (*x*-axis). Orange dots represent surface tension jumps, and the other colors of dots represent drag-involving jumps. The performances of drag-involving jumps were calculated for various size of air bubble surrounding the leg. The radius ratio of 5.05, 3.5, 2.24, and 1 (i.e., no bubble situation) are represented as light blue, blue, dark blue, and black dots, respectively. The red vertical shades represent the ranges of the observed leg angular velocity (ω_e). The gray vertical shades represent the range of the hypothetical leg angular velocity (ω_t) for *A. paludum* using drag in their jumps, and for the other large species using surface tension jumps. The angular velocity of leg rotation, ω_e, values were determined from slow motion jumping videos as explained in the Supplementary Materials PART 14 and the values are listed in Table S9.

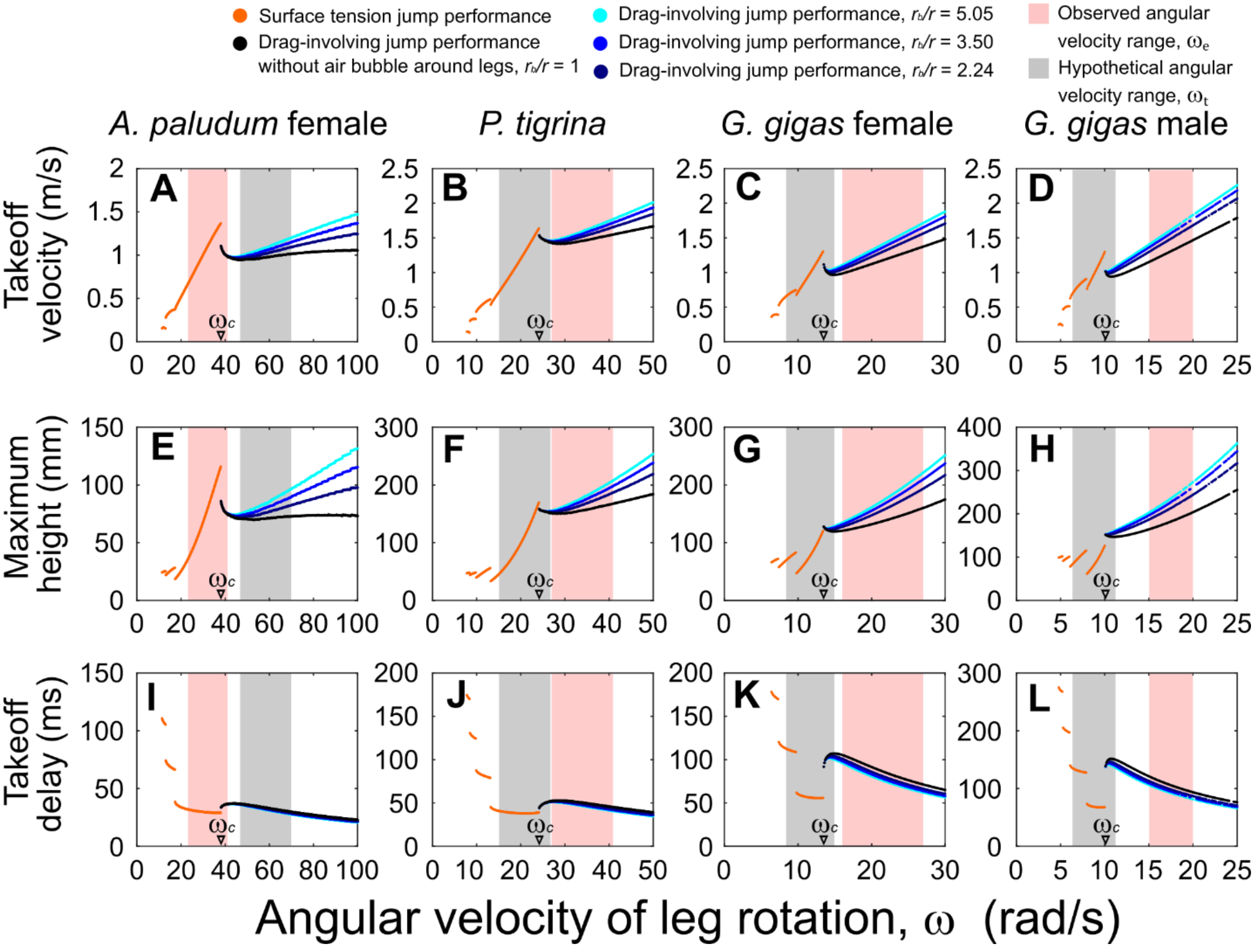

Fig. S25. Theoretically predicted jump performance as a function of midleg angular velocity for four classes of water striders' body size based on *A. paludum* females, *P. tigrina*, *G. gigas* females, and *G. gigas* males when E = 5 GPa. Jump performance measured by three variables calculated by the model: takeoff velocity (*A-D*), maximum jump height (*E-H*), takeoff delay (*I-L*). Average empirical values (mass, leg length for each leg section, leg radius, initial height of the body; average values are shown in Tables S1, S3) for each body size class were used to simulate the jumps for each body size class across a wide range of angular velocity of leg rotation (*x*-axis). Orange dots represent surface tension jumps, and the other colors of dots represent drag-involving jumps. The performances of drag-involving jumps were calculated for various size of air bubble surrounding the leg. The radius ratio of 5.05, 3.5, 2.24, and 1 (i.e., no bubble situation) are represented as light blue, blue, dark blue, and black dots, respectively. The red vertical shades represent the ranges of the observed leg angular velocity (ω_e). The gray vertical shades represent the range of the hypothetical leg angular velocity (ω_t) for *A. paludum* using drag in their jumps, and for the other large species using surface tension jumps. The angular velocity of leg rotation, ω_e, values were determined from slow motion jumping videos as explained in the Supplementary Materials PART 14 and the values are listed in Table S9.

Supplementary Materials PART 16: Examples from the model simulations of the repeated “cycle” of dimple depth and body velocity fluctuations.

The simulation results show that the performance of surface tension jumps (takeoff velocity, maximum height, and takeoff delay) for the lower range of the angular velocity of leg movement has discontinuities as the angular velocity of leg rotation changes (Fig. 5). We propose the following explanation of this phenomenon (see details in Fig. S26). During a jump, the dimple depth under insect's leg is initially getting deeper, leading to larger upward force which causes faster upward movement of the body. As the body ascends, the dimple depth becomes shallower because the leg is pulled upward from the water surface at a faster speed than the downward leg rotation. This leads to the weaker upward force causing decrease of body upwards acceleration to the point when gravitation slows the upward speed of the body, allowing the legs to “catch up” and to start pushing against the water surface increasing the dimple and the force. This repeated “cycle” of dimple depth and body velocity fluctuations can happen several times depending on the angular velocity of leg rotation. The takeoff velocity, maximum height, and takeoff delay change in an abrupt manner between jumps with different numbers of those cycles. These discontinuities do not happen if the downward leg rotation is sufficiently fast to always counteract the upward body velocity until the near end of the jump.

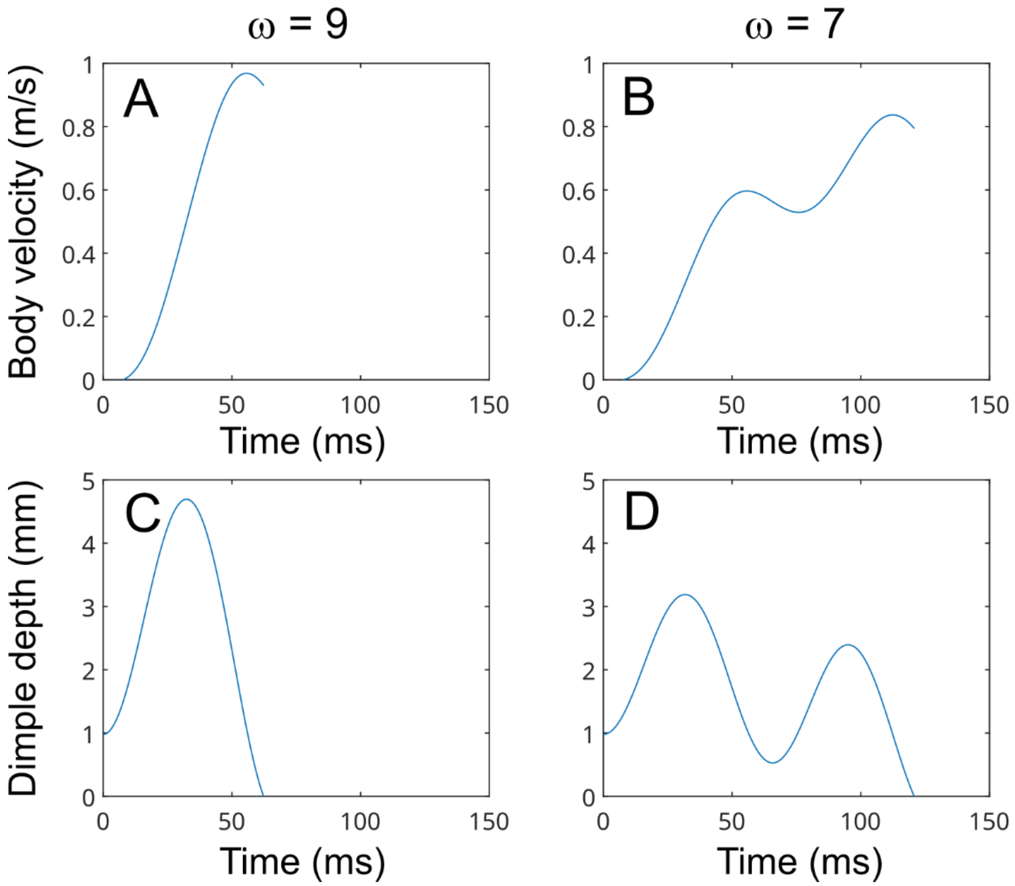

Fig. S26. Examples of model simulation illustrating a repeated “cycle” of dimple depth and body velocity fluctuations during relatively short angular midleg velocities (b, d) compared to the changes of dimple depth and body velocity for larger angular leg velocity for water striders' body size based on *G. gigas* males. The figure illustrates body velocity (*A*, *B*) and dimple depth (*C*, *D*) of jumps when the angular velocities of leg rotation are 9 (*A*, *C*) and 7 (*B*, *D*). When the angular velocity of leg rotation is not high enough (*B*, *D*), one “cycle” cannot generate sufficient force for takeoff, causing the body to decelerate before takeoff (*B*) and resulting in the deepening of the dimple again (*D*). This phenomenon causes performance discontinuities of surface tension jump in Fig. 5, S24, and S25. These discontinuities do not happen if the downward leg rotation is sufficiently fast to always counteract the upward body velocity until the near end of the jump (*A*, *C*) The final performance of these jumps in (*A*, *C*/*B*, *D*) is illustrated in Fig. 5*D*.

Supplementary Materials PART 17: Maximum jumping performance of fish.

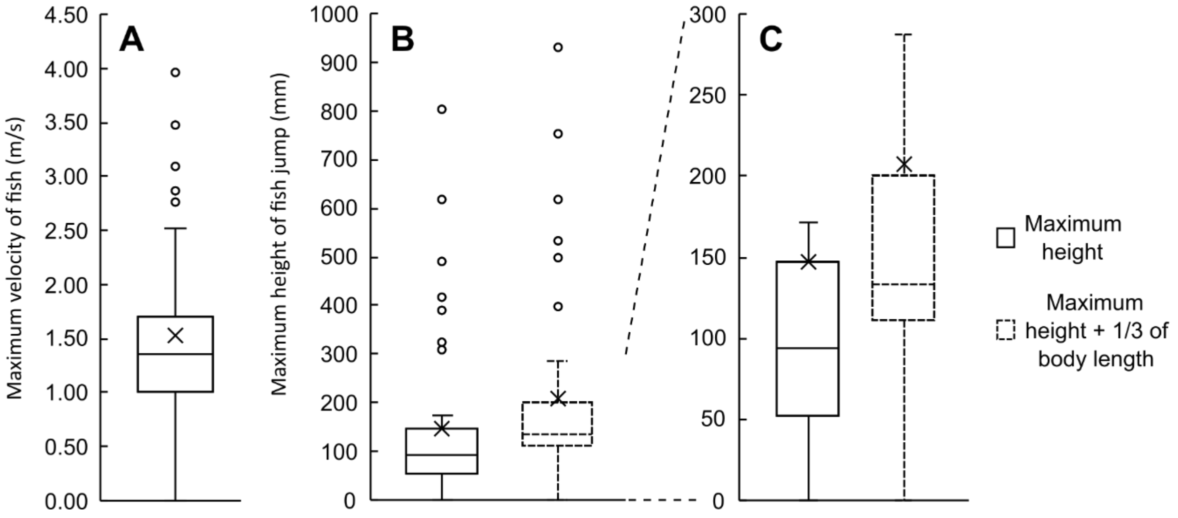

Fig. S27. The calculated hypothetical maximum performance of fish based on the literature on fish movement speeds (4). (*A*) Maximum velocity achieved by fish in the water; (*B*, *C*) the estimated hypothetical height of jumping fish assuming that a fish of a given body length moves vertically upward with the body velocity recorded in the literature (shown in *A*) and calculated according to the formula explained in Supplementary Materials PART 10. The height from the water surface is represented by solid lines. The height including 1/3 of body length is represented by dashed lines by assuming fish lose their thrust when 1/3 of their body came out from the water.

Supplementary Materials PART 18: Weber number of the study species: *Gigantometra gigas*, *Ptilomera tigrina*, and *Aquarius paludum.*

Table S10. Weber number of jumps respective to each size classes (same analyzed jumps from Fig. 6*A*). Calculation method for the Weber number was implemented from (5).

Size class	Video	ρ (kg/m^3)	σ (N/m)	w, Basal tibia thickness (m)	U (m/s)	$We = \rho U^2 w/\sigma$ (5)
G. gigas male	EVT05 (2)	998	0.072	0.00039	0.60	1.95
G. gigas male	EVT16	998	0.072	0.000455	0.49	1.51
G. gigas male	EVT41	998	0.072	0.000475	0.52	1.78
G. gigas female	EVT28	998	0.072	0.000405	0.39	0.85
G. gigas female	EVT33	998	0.072	0.00036	1.17	6.83
G. gigas female	EVT35	998	0.072	0.00036	0.46	1.06
P. tigrina	C0046	998	0.072	0.000261	0.73	1.93
P. tigrina	C0049	998	0.072	0.000261	0.65	1.53
P. tigrina	C0066	998	0.072	0.000327	0.51	1.18
A. paludum female	P_Female_evt25	998	0.072	0.000176	0.09	0.02
A. paludum female	P_Female_evt32	998	0.072	0.000194	0.28	0.21
A. paludum female	P_Female_evt33	998	0.072	0.000194	0.32	0.28

Supplementary Materials PART 19: Detailed description of the mathematical model of jumping.

p. 34-44.

I. Introduction

The mechanics of jumping of mid-sized Palearctic water striders, such as *Gerris latiabdominis*, *G. gracilicornis*, *Aquarius remigis* and *A. paludum* (Fig. S28*A*) on water has been previously studied (6). It has been shown that their leg stroke speeds are optimized to maximize their jumping speed and minimize time to take off given their mass and leg length (1), and that individual water strider are able to adjust their angular velocity of midlegs based on previous jumping experience (7). By pressing the water surface until just before it breaks under water strider legs, these typically studied water striders make a full use of capillary forces that the water surface provides.

Water strider legs may be approximated as long thin cylinders (see also Supplementary Materials Part 7: Fig. S10, 11 for link to empirically measured leg diameter and length**)**. The surface is pierced when a very thin cylinder of a radius $r \ll l_c$ is pressed downward against the water surface in a quasi-static manner to a distance of the order of the capillary length $l_c = [\sigma/(\rho g)]^{1/2}$ with σ and ρ respectively being the surface tension coefficient and density of water, and g being the gravitational acceleration. When the legs sink into the water surface, the drag forces act on the legs, which are significantly smaller than the capillary forces for the mid-sized striders.

While the mid-sized water striders do not break water surfaces for efficient jumps, the larger species such as *Gigantometra gigas* (Fig. S28*B*) do not follow the aforementioned rule of motion in jumping. *G. gigas* is up to ten times heavier than mid-sized water striders in leg length. Typical mass and middle leg length of *G. gracilicornis* (Fig. S28*A*) are respectively 30 mg and 20 mm, whereas the giant water striders are up to 500 mg and 100 mm for *Gigantometra gigas* (Fig. S28*B*). Figure S29 shows a sequence of the jump of a *G. gigas* on water taken by a high-speed camera in a field experiment. We see that the middle legs pierce the water surface to a significant degree, which is not observed for mid-sized striders. Here, we describe the kinematic models of the two pairs of legs separately, and combine the models to predict the jump dynamics of the *G. gigas* and other water striders with similar jumping behavior.

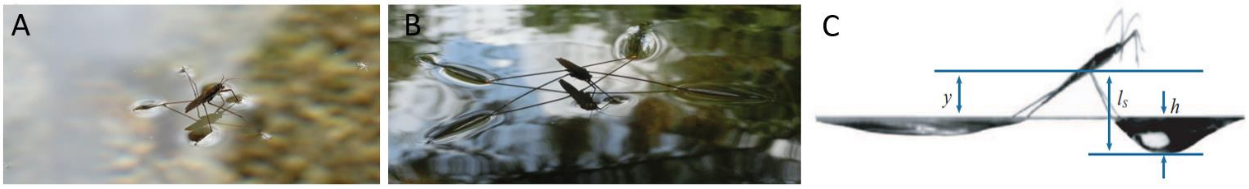

Fig. S28. Two different-sized water striders and basic parameters. (*A*) A mid-sized water strider, *Gerris gracilicornis*. (*B*) A gigantic water strider, *Gigantometra gigas*. (*C*) A side view of a *G. gigas* during its jump with parameters used in the theoretical model. Symbols and variables used in the model are explained in Table S11 and Fig. S32.

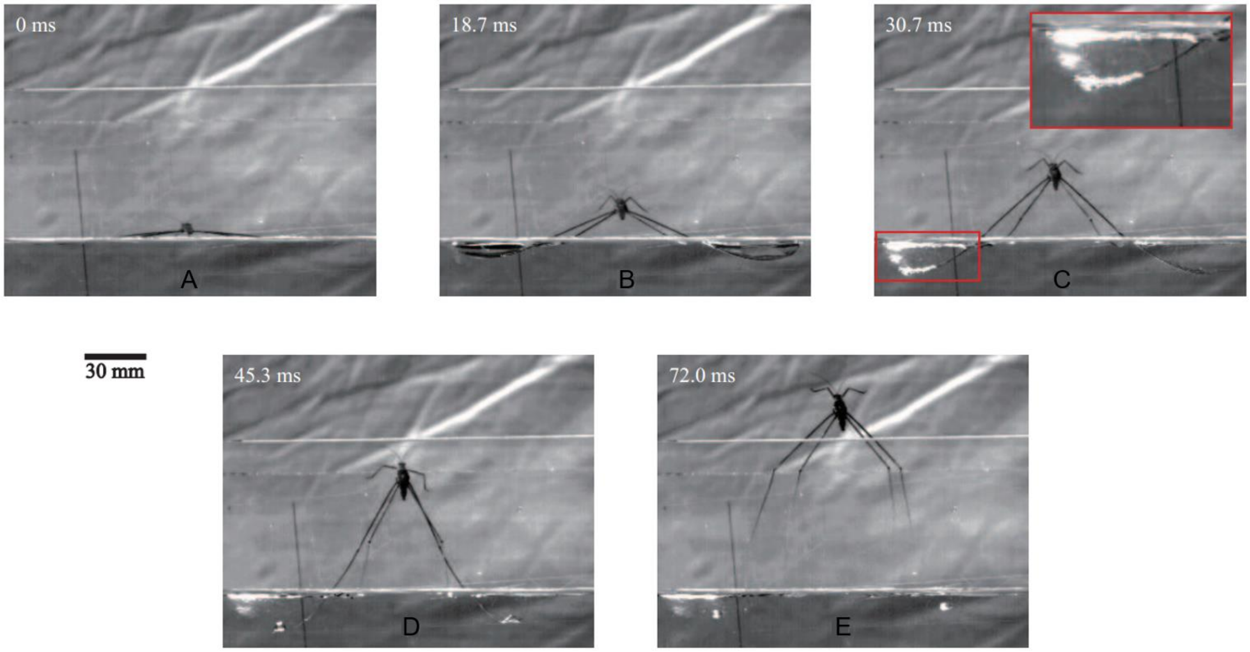

Fig. S29. A sequence of the jump of *G. gigas* on water. (*A*) The initial posture of the *G. gigas* before jumping. (*B*) Surface tension phase. The middle and hind legs create dimples on the water surface. (*C*) The early stage in the drag phase. The middle legs break the water surface with air bubbles covering the legs (magnified image) until they reach the deepest location. (*D*) The late stage in the drag phase. The air bubbles surrounding the middle legs are absent. (*E*) Gravity driven phase. All the legs are completely disengaged from the water surface.

II. Kinematics of middle and hind legs

First, we consider the kinematics of middle legs. We assume that their movements comprise three phases: the surface tension phase, the transition phase, and the drag phase. In the surface tension phase, the middle legs push the water surface down with a constant wetted length, l_m (the length of tibia plus tarsus of the middle leg). Symbols and variables used in the model are explained in Table S11 and Fig. S32 in part IV. The dimple depth generated by the middle leg, h, grows, leading to the increase of the body center height, y, with time, t (Fig. S28*C*). As the angular velocity of midleg's downward rotation can be approximated to be a constant, ω (1), the downward linear velocity of the middle leg relative to the body center, v_m, can be written as:

$$v_m = \dot{l_s} = \omega(l_l - y_i)\sin(2\omega t), \quad (1)$$

where $l_s = y + h$ is the vertical distance from the body center to the tip of the leg. l_l is the entire length of the leg consisting of femur, tibia, and tarsus, and y_i is the initial height of the body centre from the undisturbed free surface. Integrating v_m over time, t, gives:

$$l_s = \frac{1}{2}(l_l - y_i)[1 - \cos(2\omega t)] + y_i \text{ for } l_s = y_i \text{ at } t = 0. \quad (2)$$

Based on empirical leg measurements, we model the wetted middle legs as cylinders of diameter, d, and length, l_m, according to the details described in the Supplementary Materials PART 7. The water surface cannot withstand the depression of cylindrical legs when the dimple reaches a critical depth, h_c, which was determined by empirical measurement for different wetted leg length (Fig. S13*A*, Supplementary

Materials PART 8). When the dimple depth, $h = l_s - y$, exceeds h_c at time t_c, in the model, then the wetted part of the middle leg starts to pierce the water surface, entering the transition phase. In this phase, there are both sunk and unsunk part of middle leg. The unsunk part is supported by surface tension, while sunk part experiences drag. We assumed that the proportion of wetted leg for surface tension, p_s, and for drag, p_d, gradually changes from only surface tension to only drag, i.e., from the start of sinking to completely sunk leg. The duration of this changing proportion was determined by empirical measurement (Fig. S14, Supplementary Materials PART 8). After the transition phase, the drag phase begins at time t_d. During this third phase, the middle legs can only provide drag.

To calculate drag in both transition and drag phase, the middle legs are considered almost straight with the wetted length decreasing according to formula:

$$l_1 = l_l - \frac{y}{\cos\left(\frac{\pi}{2} - \omega t\right)}, \qquad (3)$$

that takes into account the ascent of the insect body. The downward linear velocity of a middle leg relative to the water surface is then given by:

$$v_l = \dot{l_s} - \dot{y} = v_m - \dot{y} = \omega(l_l - y_i)\sin(2\omega t) - \dot{y}. \qquad (4)$$

Because the legs penetrate the water with a high velocity, an air bubble forms around the leg, as shown in Fig. S29*C*. We assume in the model that the air bubble detaches after the moment when the middle legs reach the deepest point in the water. Thus, the effective frontal area, the projected area of the leg with its diameter, d, along its moving velocity, is $A_f = d_b l_1$, thanks to the presence of an air bubble that increases the cylindrical leg diameter, to d_b ("*b*" stands for bubble of air) by the factor of 2.24-5.05 times (d_b=3.5*d in average value) as determined in empirical measurements (see Supplementary Materials PART 7: Table. S7).

We turn to the kinematics of hind legs which do not pierce the water surface during the jump. The stroke can be decomposed into two phases. In the first (pushing) phase, the hindlegs push the water surface down with a fully contacted constant wetted length, l_h (the length of tibia plus tarsus of the hind leg), with a growing dimple depth. We assume in the model that the depth of dimple created by a hind leg from the undisturbed free surface, h_h, grows at the same rate as dimple of the midleg until it reaches constant depth, h_{hm}. Constant depth of hindlegs is calculated using observed empirical maximum depth of hindlegs, h_{hE}, and wetted length of hindlegs assuming leg as half of an arc (see details in Supplementary Materials PART 9).

In the second phase, which starts when the dimple depth reaches its constant, h_{hm}, the legs slide on the water surface towards the body while detaching themselves from the surface. Thus, the wetted length eventually decreases by ascending the body while the dimple depth is constant. We calculate the wetted length of a hind leg, l_2, based on body heights, y, constant wetted length of a hind leg, l_h, and femur length of a hind leg, l_{Hfemur}. We use a simplifying assumption that the hindlegs that are out of the water align with femur along the direction of jump and are being dragged out from the water surface vertically (Fig. S30, Supplementary Movie 1), while the hindleg section on the water surface is bent creating a dimple without surface breaking. Therefore, the wetted length of a hind leg approximately follows:

$$l_2 = l_h - (y - l_{Hfemur}) \text{ when } y > l_{Hfemur} \; and \; l_h > (y(t) - l_{Hfemur}). \qquad (5)$$

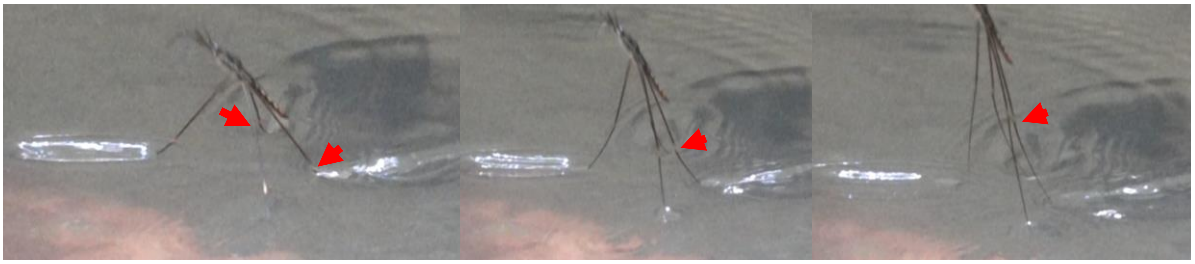

Fig. S30. Field jump of *G. gigas*. Field jump of *G. gigas* shows its hindlegs are almost vertical when the wetted length is pulled out from the water surface. Red arrows mark the femur-tibia joint.

Summarizing the simplified kinematics of both middle and hind legs in the model, we schematically plot the timeline of different phases of the jump for the four legs as shown in Fig. S32. In the stationary phase, $t = 0$, both middle and hindlegs are in stationary situation with initial dimple depth, h_o. In the surface tension phase, $0 < t < t_c$, both the middle and hind legs moving with angular velocity, ω, are pushing the water surface with growing of dimple, and only dimple depth of hind leg, h_h, stops growing when it reaches specific depth, h_{hm}. In the transition phase, $t_c < t < t_d$, where t_d is the moment when the breaking ends, the unsunk part of the middle leg is supported by surface tension, while the sunk part experiences drag. The proportion of the sunk and unsunk length of the middle legs changes gradually during this phase. In the drag phase, $t_d < t < t_f$, where t_f is the moment of take-off, the middle legs continue to move in water with an angular velocity, ω, and are surrounded by air bubbles. The hind legs are being closed on the water surface with their wetted length being decreased with constant dimple depth, h_{hm}.

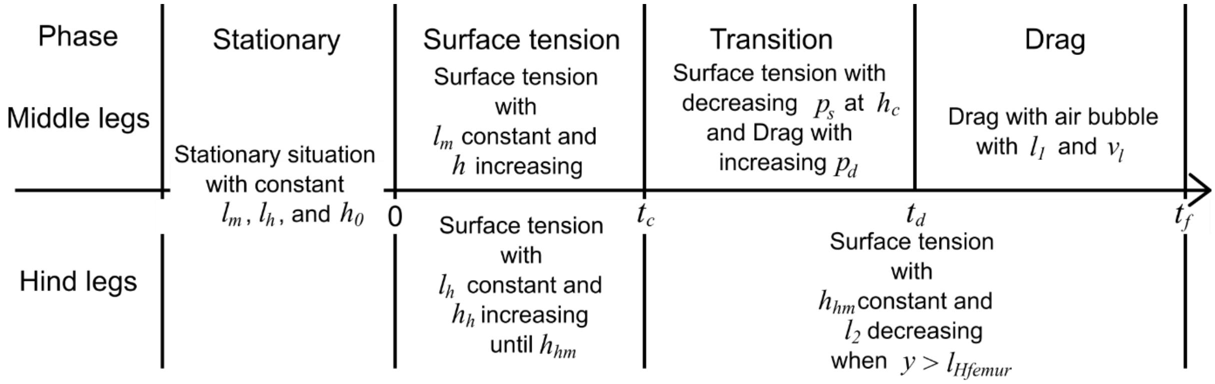

Fig. S31. Timeline of the simulated phases of the jump for the middle and hind legs. Middle and hind legs contribute force resulting from surface tension to the jump until the critical moment of time when water surface is broken, t_c. After this moment of time, middle legs of diameter, d_b, resulting from the presence of the air bubble, create drag force until the moment, t_f. Between the moment of t_c and t_d, middle legs utilize surface tension and drag with unsunk and sunk parts, respectively. After t_d, the dimple under the middle legs is completely broken, and the middle legs exploit drag only. Hindlegs create force resulting from surface tension during the whole period from t_c to t_f. This force gradually decreases as the wetted leg length, l_2, decreases, while dimple depth, h_{hm}, is assumed constant.

III. Jump dynamics of *Gigantometra gigas*

SURFACE TENSION PHASE

Symbols and variables used in the model, including geometric schematics for some of the variables, are explained in Table S11 and Fig. S32 in part IV. The water strider ascends from the water surface because the interaction of its legs and water produces upward thrust. Newton's second law of motion dictates $F = m\ddot{y}$, where F is the total force acting on the water strider legs and m is the water strider mass. We find the temporal evolution of the body center height and the take-off velocity by analyzing the forces produced by the movement of legs of angular velocity, ω.

During the "Surface tension phase" (Fig. S32) of the simplified jump, various forces are exerted on the legs including the capillary force $F_c \sim \sigma l_w$, pressure force $F_p \sim \rho U^2 d l_w$, buoyancy $F_b \sim \rho g d h l_w$, added inertia $F_a \sim \rho d^2 l_w U^2 / h$, viscous force $F_v \sim \mu r l_w U / l_c$, and the weight of the water strider (for the large *G. gigas* males it is ~ 5 mN). Here, l_w is the wetted leg length, and U is the rate of the vertical growth of dimple, which is a direct consequence of downward linear velocity of the middle leg, v_l that according to formula (4) depends on, among others, on the leg angular velocity, ω.

Using the typical values for middle legs $d = 260$ µm, $l_w = 53.5$ mm, $h = 5$ mm, and $U = 0.4$ ms^{-1}, we found that the capillary force dominates the other forces, and we decided to ignore the other forces in the simplified model.

The capillary force acting on a pair of floating flexible cylinders is given by formula 6 below, which is a modified formula from Yang et al. (1) based on model for a cylinder by Vella et al. (3).

$$F_c = 4C\rho g l_c l_w h \left[1 - \left(\frac{h}{i l_c}\right)^2\right]^{1/2}, \qquad (6)$$

where C is the flexibility factor depending on the scaled leg length $L_f = l_w / l_e$. Here, $l_e = (B l_c / \sigma)^{1/4}$ is the modified elastocapillary length of the leg with the bending rigidity $B = \pi E d^4 / 64$ and E being Young's modulus of insect cuticle. We approximate $C \approx (1 + 0.082 L^{3.3})^{-1}$ for $L_f < 2$ and $C \approx (0.88L)^{-1}$ for $L_f > 2$. In comparison to the original model of Yang et al. (1), we modified the denominator in the formula 6 from $2l_c$ to il_c, by introducing the index of maximum dimple depth, $i = h_M / l_c$. The maximum dimple depth at surface breaking moment, h_M, was empirically derived for water striders of different sizes using the linear regression of h_M on the constant wetted length of midleg ($h_M = 0.1227 l_m + 0.004$; Fig. S13*B*, Supplementary Materials PART 8). This index allowed us to extend the range of the dimple depths beyond the mathematical limitation of $h \leq 2l_c$ from the original model (1).

We first model the stationary situation, "Stationary phase" (Fig. S31). We assume the stationary dimple depth of each individual by calculating force balance between gravity and surface tension. When the water strider is on the water surface using their two middle legs and two hind legs, the stationary dimple depth, h_0, satisfies the following formula by assuming the same dimple depth for middle and hind legs:

$$mg = 4\rho g l_c \left\{ C_{m0} l_m h_0 \left[1 - \left(\frac{h_0}{i l_c}\right)^2\right]^{1/2} + C_{h0} l_h h_0 \left[1 - \left(\frac{h_0}{i l_c}\right)^2\right]^{1/2} \right\}. \qquad (7)$$

In the surface tension phase, the dimple depth is given by $h = l_s - y$, leading us to write $\ddot{h} = \ddot{l_s} - (F - g)/m$. Here, F is the sum of the capillary forces acting on the middle and hind legs:

$$F = 4\rho g l_c \left\{ C_m l_1 h \left[1 - \left(\frac{h}{il_c}\right)^2\right]^{1/2} + C_h l_2 h_h \left[1 - \left(\frac{h_h}{il_c}\right)^2\right]^{1/2} \right\}. \qquad (8)$$

This gives a second-order nonlinear differential equation for h with the initial conditions of $h(t = 0) = h_0$ *and* $\dot{h}(t = 0) = 0$, which we solve using Matlab. Then we get the body centre height $y = l_s - h$ as a function of time for $0 < t < t_c$ (i.e., $h < h_c$).

TRANSITION PHASE

Once the middle legs start to pierce the water surface, $t > t_c$ (i.e., $h > h_c$; where h_c is empirically established for each water strider size; Fig. S13*A*), the middle legs experience the drag force F_d of water in addition to the capillary force. The drag force acting on a pair of middle legs moving with the velocity $v_l = \omega(l_l - y_i)\sin(2\omega t) - \dot{y}$ as obtained above is given by

$$F_d = \rho C_D A_f v_l^2, \qquad (9)$$

where $C_D = 0.8$ is the drag coefficient on the flexible cylinder (8), taken to be about 30% lower than the value for a rigid cylinder at a Reynolds number, $Re = \frac{\rho v_l d}{\mu} \approx 100$. We simply assumed that the drag coefficient of the middle legs is the same as that of a solid cylinder. This is because calculating the exact drag coefficient of the middle legs would require detailed analysis of the movement of air inside the bubble around the leg and between hairs, which is beyond the scope of our study. The frontal area is $A_f = d_b l_1$ in transition and drag phase, $t_c < t < t_f$.

During the transition phase ($t_c < t < t_d$), the middle legs utilize both capillary and drag forces as the legs progressively sink. We assumed that the leg sinks continuously during a certain breaking duration, D_b, which was calculated from the wetted length using linear regression of empirical measurements (Fig. S14, Supplementary Materials PART 8). Thus, the functional wetted leg length for each force is linearly changed by introducing the proportion of wetted leg length for utilizing surface tension, p_s, and for drag, p_d ($p_s + p_d = 1$). During the breaking duration, D_b, the proportion of wetted leg length for utilizing surface tension, p_s, linearly decreases from 1 to 0, while the proportion of wetted leg length for utilizing drag, p_d, linearly increases from 0 to 1. For a given moment, we write $p_s = (t_c + D_b - t)/D_b$ and $p_d = 1 - p_s$. In this phase, the dimple depth for capillary force is fixed at h_c since we observed that the breaking of the dimple expands laterally (as shown in Fig. S12 in Supplementary Materials PART 8).

Then the total force acting on the middle and hind legs becomes

$$F = 4\rho g l_c C_m p_s l_1 h_c \left[1 - \left(\frac{h_c}{il_c}\right)^2\right]^{1/2} + \rho C_D d_b p_d l_1 v_l^2 + 4C\rho g l_c l_2 h_h \left[1 - \left(\frac{h_h}{il_c}\right)^2\right]^{1/2}. \qquad (10)$$

DRAG PHASE

In the drag phase, after the dimple is completely broken, $t > t_d$, the proportion of wetted leg for utilizing surface tension, p_s, becomes 0 and middle leg utilize drag only.

Then the total force acting on the middle and hind legs naturally becomes

$$F = \rho C_D A_f v_l^2 + 4C\rho g l_c l_2 h_h \left[1 - \left(\frac{h_h}{il_c}\right)^2\right]^{1/2}. \qquad (11)$$

In the transition and drag phase, solving $\ddot{y} = (F - g)/m$, a second-order differential equation with A_f, v_l, l_2, h_h being functions of y and t, gives the body center height versus time. The initial conditions are provided from the results of the surface tension phase. From the relationship between the body center height versus time we predict time of take-off, t_f, and body speed at v_f. From the body speed and body mass, we predict that maximum jump height above the water surface as $H_m = y_i + \frac{v_f^2}{2g}$ (Supplementary Materials PART 11**)**. These model predictions can be calculated for various vales of angular leg velocities, and for water striders of various body mass and leg lengths.

IV. Explanations of the symbols used in the paper

The symbols and variable names used in the model are listed here in Table S11, and additionally some of them are shown in a graphical schematic in Fig. S32. The font colors in the Table S11 correspond to the colors used in the Fig. S32.

Table S11. Explanations of the symbols in the model

r	Radius of legs as cylinder
σ	Surface tension coefficient of water
ρ	Density of water
g	Gravitational acceleration
$l_c = [\sigma/(\rho g)]^{1/2}$	Capillary length
l_w	Wetted length of the leg
l_l	Entire length of the middle leg consisting of femur, tibia, and tarsus
l_m	Constant wetted length of middle leg (the length of tibia plus tarsus of the middle leg)
l_h	Constant wetted length of hind leg (the length of tibia plus tarsus of the hind leg)
l_{Hfemur}	Femur length of hind leg
l_2	Dynamic wetted length of a hind leg
$l_s = y + h$	Vertical distance from the body center to the tip of the leg
$l_1 = l_l - y/cos(\pi/2 - \omega t)$	Decreased wetted length of middle leg for ascent of the body
$l_e = (Bl_c/\sigma)^{1/4}$	Modified elastocapillary length of the leg
h	Dynamic dimple depth generated by the middle leg
h_c	Critical dimple depth
h_o	Constant dimple depth at stationary situation
h_M	Maximum dimple depth at surface breaking moment
h_B	The breaking point depth
h_h	Dynamic dimple depth created by a hind leg
h_{hE}	Maximum dimple depth of hind leg by empirical observations
h_{hm}	Constant dimple depth of hind leg derived from h_{hE}
i	Index of maximum dimple depth; $il_c = h_M$
t	Time
t_c	Critical moment of the start of water surface breaking
t_d	Last moment of complete water surface breaking
t_f	Moment of take-off
D_b	Duration of dimple breaking
p_s	The proportion of wetted leg for utilizing surface tension (i.e., proportion of the length of unsunk part from total wetted leg length); $p_s = (t_c + D_b - t)/D_b$
p_d	The proportion of wetted leg for utilizing drag (i.e., proportion of the length of sunk part from total wetted leg length); $p_d = 1 - p_s$
y	Body center location on vertical coordinate axis
$\dot{y}$	Time derivative of y in Newtonian calculus notation; vertical speed of body center
y_i	Initial height of the body center from the undisturbed free surface
m	Mass of the water strider
r	Radius of the wetted middle leg as a cylinder
r_b	Radius of the wetted middle leg as a cylinder surrounded by the air bubble
A_f	Projected area of the leg

ω	Angular velocity of middle leg rotation of a jump
ω_e	Derived angular velocity of middle leg rotation in a jump under the assumption that empirically measured linear downward velocity of wetted midleg relative to water surface, and the vertical distance from the body center can be approximated using a constant value of ω, by two formulae: $v_l = \omega(l_l - y_i)sin(2\omega t) - \dot{y}$, $l_s = \Delta l[1 - \frac{1}{2}\cos(2\omega t) + y_i]$.
ω_t	Hypothetical velocity of midleg rotation of the hypothetical jumps (i.e., surface tension jumps of *G. gigas* and *P. tigrina*; drag-involving jump of *A. paludum*)
v_m	Downward linear velocity of the middle leg relative to the body center
v_f	Take-off velocity
$v_l = \omega(l_l - y_i)sin(2\omega t) - \dot{y}$	Downward linear velocity of a middle leg relative to the water surface
U	Rate of the vertical growth of dimple
μ	Dynamic viscosity
F_c	Capillary force
F_p	Pressure force
F_b	Buoyancy
F_a	Added inertia
F_v	Viscous force
$L_f = l_w/l_e$	Scaled leg length
$B = \pi E d^4/64$	Bending rigidity
E	Young's modulus of insect cuticle
C	Flexibility factor; function of wetted length of a leg, l_w, and its bending rigidity, B
C_{m0}	Middle leg flexibility factor; function of wetted length of a middle leg, l_m, and its bending rigidity, B
C_{h0}	Hind leg flexibility factor; function of wetted length of a hind leg, l_h, and its bending rigidity, B
C_m	Middle leg flexibility factor; function of wetted length of a middle leg, l_1, and its bending rigidity, B
C_h	Hind leg flexibility factor; function of wetted length of a hind leg, l_2, and its bending rigidity, B
C_D	Drag coefficient
Re	Reynolds number
H_m	Maximum height of the jump
$\Delta l_l = l_l - y_i$	Maximal downward reach of the middle leg
L	Downward stroke; dimensionless maximal reach of the average of four legs
$\Omega = \omega(l_c/g)^{1/2}$	Dimensionless angular velocity of the average four legs' rotation of a jump
$M = m/(\rho l_c^2 C l_w)$	Dimensionless index of insect body mass with respect to the leg; body mass with respect to maximal water mass can be displaced by the average of four legs
$L_m = \Delta l_l/l_c$	Midleg downward stroke; dimensionless maximal reach of the middle leg
$\Omega_\mathrm{m} = \omega_e(l_c/g)^{1/2}$	Dimensionless angular velocity of middle leg rotation of a jump
$M_m = m/(\rho l_c^2 C_{m0} l_m)$	Dimensionless index of insect body mass with respect to the middle leg; body mass with respect to maximal water mass can be displaced by the middle leg

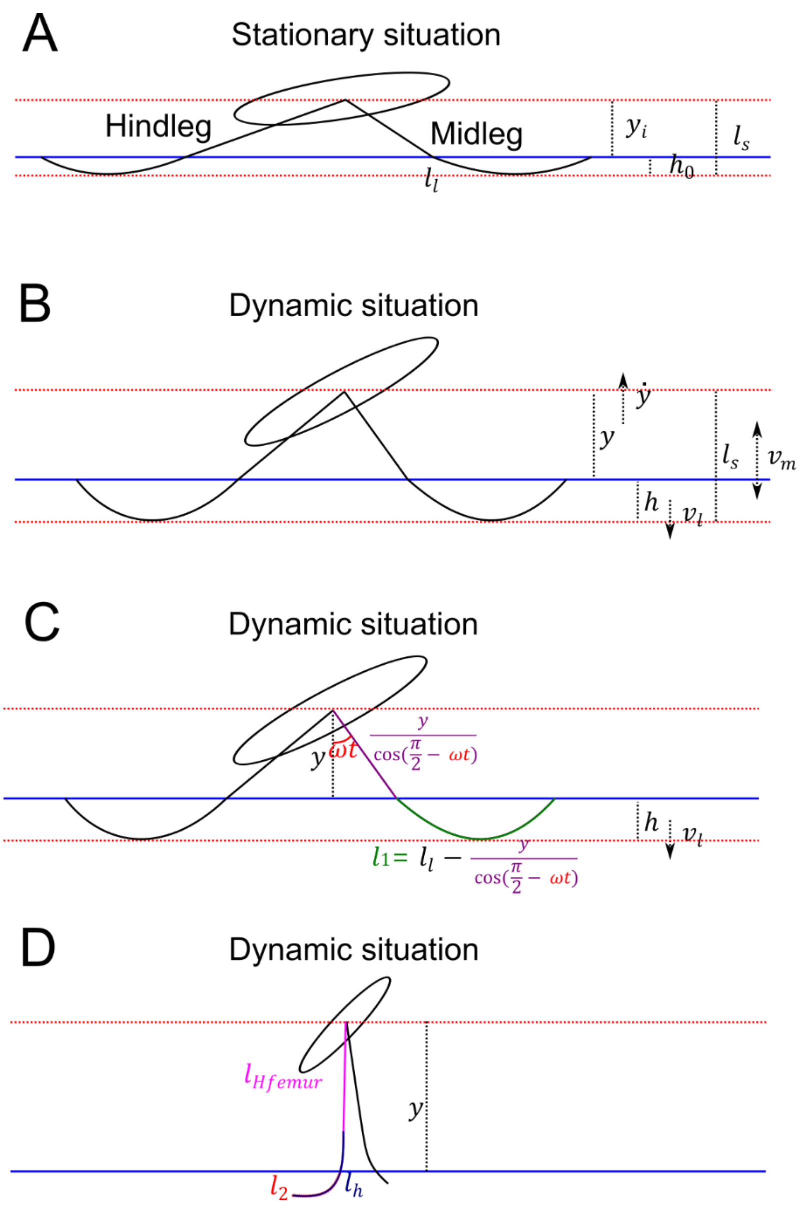

Fig. S32. Schematics of geometric parameters. Geometric parameters used in the mathematical model in stationary situation (*A*) and dynamic situations (*B*, *C*, *D*). (*A*) illustrates stationary phase with initial height, $\boldsymbol{y_i}$, initial dimple depth, $\boldsymbol{h_0}$. (*B*) illustrates dynamic variables during jumping situations: surface tension, transition, and drag phase. (*C*) illustrates the functional leg length for drag calculation in formula 3. Please note that while the drawing (*C*) shows a bent leg for dimple depth, $\boldsymbol{h}$, and downward velocity, $\boldsymbol{v_l}$, the leg length for drag, $\boldsymbol{l_1}$, was assumed to be a solid cylinder. (*D*) illustrates hindleg's dynamic wetted length, $\boldsymbol{l_2}$, in formula 5.

V. Model diagram

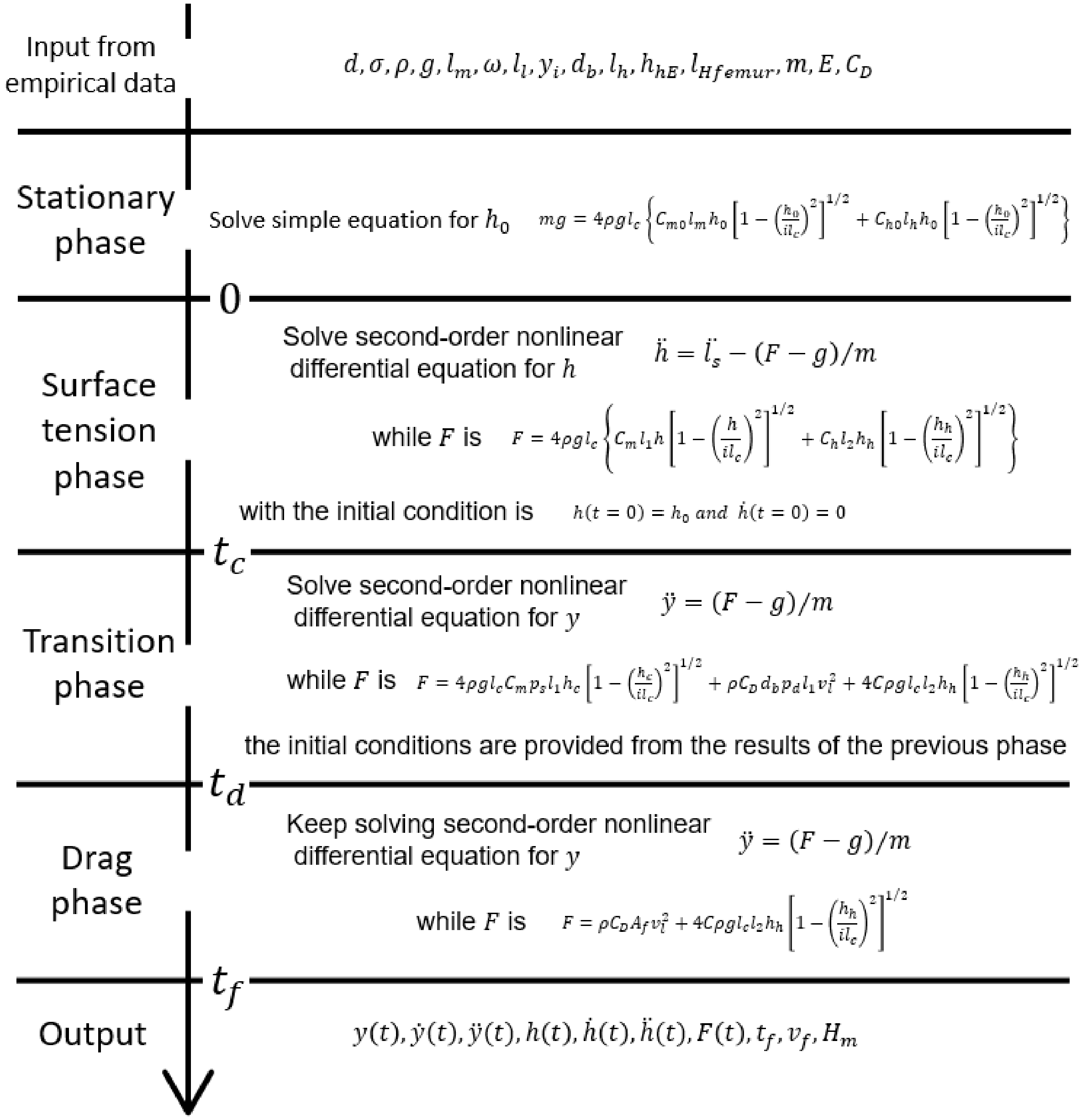

Fig. S33. Simplified diagram of model workflow. After input of the empirical data, in the stationary phase, initial dimple depth, $\boldsymbol{h_o}$, is calculated by solving simple equation for providing initial condition for the surface tension phase. In the surface tension phase, $\boldsymbol{h}$, $\boldsymbol{y}$, and $\boldsymbol{F}$ are calculated in the range of $\boldsymbol{0 < t < t_c}$, by solving second-order nonlinear differential equation for $\boldsymbol{h}$. In the transition and drag phase, $\boldsymbol{h}$, $\boldsymbol{y}$, and $\boldsymbol{F}$ are calculated in the range of $\boldsymbol{t_c < t < t_f}$, by solving second-order nonlinear differential equation for $\boldsymbol{y}$. The initial condition of the transition phase is fixed by calculation of the surface tension phase. Model provides distribution of body height, $\boldsymbol{y}$, dimple depth, $\boldsymbol{h}$, force, $\boldsymbol{F}$, by time and take-off time, $\boldsymbol{t_f}$, take-off velocity, $\boldsymbol{v_f}$, and maximum height, $\boldsymbol{H_m}$. The model simulations were conducted in Matlab. The Matlab code is available at https://doi.org/10.5281/zenodo.7847879.

VI. Values of empirical parameters used in model simulations

Table S12. Empirical parameters used to model jump in each video that has been analyzed in detail.

Parameter/ variable (units)	*G. gigas* male			*G. gigas* female			*P. tigrina*		
	EVT05 (2)	EVT16	EVT41	EVT28	EVT33	EVT35	C0046	C0049	C0066
σ (N/m)	0.072								
ρ (kg/m^3)	998								
g (m/s^2)	9.8								
E (N/m^2)	1e10								
C_D	0.8								
r (m)	11.3e-5	13.1e-5	13.7e-5	11.7e-5	10.4e-5	10.4e-5	7.5e-5	7.5e-5	9.4e-5
ω_e (rad/s)	20	15	16	16	19	17	41	33	29
m (kg)	374.76e-6	483.23e-6	325.41e-6	305.67e-6	226.81e-6	226.81e-6	134e-6	134e-6	123e-6
y_i (m)	0.00017	0.00165	0.00088	0.00333	0.00435	0.00274	0.00271	0.00473	0.00806
l_l (m)	88.64e-3	102.69e-3	103.17e-3	72.59e-3	70.13e-3	70.13e-3	44.72e-3	44.72e-3	50.63e-3
l_m (m)	45.78e-3	54.60e-3	54.05e-3	39.80e-3	38.87e-3	38.87e-3	22.70e-3	22.70e-3	25.56e-3
l_h (m)	63.36e-3	79.48e-3	77.17e-3	44.21e-3	36.98e-3	36.98e-3	16.34e-3	16.34e-3	14.46e-3
l_{Hfemur} (m)	42.74e-3	47.42e-3	48.24e-3	32.25e-3	31.11e-3	31.11e-3	24.30e-3	24.30e-3	28.88e-3
h_{hE} (m)	0.0039	0.0032	0.0062	0.0062			0.0065		
$r_b = d_b/2$	28.5e-5	52.0e-5	48.2e-5	3.5r					

Table S13. Empirical parameters used in size-specific simulations.

Parameter/variable (units)	*G. gigas* male	*G. gigas* female	*P. tigrina*	*A. paludum* female
σ (N/m)	0.072			
ρ (kg/m^3)	998			
g (m/s^2)	9.8			
E (N/m^2)	0.5e10, 1e10, 1.5e10			
C_D	0.8			
r (m)	13.14e-5	11.21e-5	8.934e-5	5.128e-5
m (kg)	413.7e-6	265.2e-6	115.4e-6	47.6e-6
y_i (m)	0.900e-3	0.900e-3	5.17e-3	3.00e-3
l_l (m)	101.9e-3	71.7e-3	47.9e-3	25e-3
l_m (m)	53.5e-3	38.5e-3	23.9e-3	13.4e-3
l_h (m)	73.5e-3	40.6e-3	19.1e-3	9.5e-3
l_{Hfemur} (m)	49.5e-3	32.1e-3	27.6e-3	12.1e-3
h_{hE} (m)	0.0041		0.0065	0.0041
$r_b = d_b/2$	2.24r, 3.5r, 5.05r			

References

1. E. Yang, J. H. Son, S. Lee, P. G. Jablonski, H.-Y. Kim, Water striders adjust leg movement speed to optimize takeoff velocity for their morphology. *Nat. Commun.* **7**, 1–9 (2016).
2. R. Matsuda, Morphology, evolution and a classification of the Gerridae (Hemiptera-Heteroptera). *Univ. Kansas Sci. Bull.* **41**, 25–632 (1960).
3. D. Vella, Floating objects with finite resistance to bending. *Langmuir* **24**, 8701-8706 (2008).
4. P. Domenici, R. W. Blake, The kinematics and performance of fish fast-start swimming. *J. Exp. Biol.* **200**, 1165–1178 (1997).
5. J. W. M. Bush, D. L. Hu, Walking on water: Biolocomotion at the interface. *Annu. Rev. Fluid Mech.* **38**, 339–369 (2006).
6. H.-Y. Kim, *et al.*, Mechanics of jumping on water. *Phys. Rev. Fluids* **2**, 1–10 (2017).
7. M. Baek, *et al.*, Water strider females use individual experience to adjust jumping behaviour to their weight within physical constraints of water surface tension. *Sci. Rep.* **10**, 1–12 (2020).
8. C. S. Subramanian, H. Gurram, P. L. Kanherkar, A CFD and Experimental Study of Thin Flexible Wire in a Cross Flow (2016).
